# Development and validation of a machine learning-based return to work predictive model for young and middle-aged maintenance hemodialysis patients

**DOI:** 10.3389/fpubh.2026.1846282

**Published:** 2026-09-24

**Authors:** Qingxin Mai, Xiaohan Ma, Ziying Yang, Chao Wang, Weisheng Gu, Hanjiao Liu, Yang Song

**Affiliations:** 1 Shenzhen Hospital of Integrated Traditional Chinese and Western Medicine, Shenzhen, China; 2 Shenzhen Clinical College of Integrated Chinese and Western Medicine, Guangzhou University of Chinese Medicine, Shenzhen, China; 3 School of Nursing, Guangzhou University of Chinese Medicine, Guangzhou, China; 4 Department of Nephrology, The First Affiliated Hospital of Guangzhou University of Chinese Medicine, Guangzhou, China

**Keywords:** machine learning, maintenance hemodialysis, predictive model, return to work, young and middle-aged

## Abstract

**Objectives:**

Return to work (RTW) is a key indicator of recovery for young and middle-aged maintenance hemodialysis (MHD) patients. Timely identification of patients with a low likelihood of RTW is crucial for precision rehabilitation assessment and risk-stratified management. This study aimed to develop and validate a machine learning-based predictive model for identifying influencing factors of current RTW status and enabling risk stratification among young and middle-aged MHD patients.

**Methods:**

From March 2023 to May 2024, 793 young and middle-aged MHD patients were recruited from eight tertiary hospitals in China, with 768 ultimately included (565 in the modeling set and 203 in the external validation set). Demographic characteristics and psychosocial factors were collected via self-administered questionnaires. Five algorithms, including logistic regression, support vector machine, random forest (RF), extreme gradient boosting, and adaptive boosting, were implemented. Hyperparameter tuning was performed using grid search combined with 10-fold nested cross-validation. The optimal algorithm was selected based on comprehensive evaluation metrics, including area under curve (AUC), calibration curves, and net benefit from decision curve analysis. Least absolute shrinkage and selection operator (LASSO) regression was subsequently applied for feature selection. After determining the final feature set, the optimal model was retrained on the modeling set. Internal validation was conducted using nested cross-validation, and external validation was performed on an independent validation set. The final model was explained using SHapley Additive exPlanations and visualized using a web-based calculator.

**Results:**

The RF model was identified as the optimal predictive model. LASSO regression further selected 10 feature variables, including RTW self-efficacy, social functioning, chronic comorbidities, social support, social isolation, family functioning, age, dialysis time period, residence and gender. The RF model was retrained using the selected features. The internal validation yielded an AUC of 0.946 (95% CI 0.892–0.997), while the external validation achieved an AUC of 0.918 (0.873–0.963), indicating good predictive performance and generalizability of the model.

**Conclusion:**

The RF model performs well in predicting current RTW probability among young and middle-aged MHD patients, enabling identification of high-risk non-RTW individuals and key contributing factors. It may serve as a practical screening tool for precision rehabilitation and stratified clinical management.

## 1 Introduction

Maintenance hemodialysis (MHD) is the primary alternative treatment for extending the life of patients with end-stage renal disease (ESRD). By 2030, the MHD patients worldwide is projected to exceed 6 million (1). As of the end of 2017, more than 60% of MHD patients were young and middle-aged adults (2). MHD is a long-term, continuous process that inevitably imposes varying degrees of physical burden and psychological stress on young and middle-aged patients, hindering meaningful participation in personal, social, and economic roles, including return to work (RTW) (3, 4).

There is a global consensus on the importance of RTW for patients with chronic diseases. However, the employment situation for young and middle-aged MHD patients remains concerning, characterized by poor employment rates and limited willingness to RTW. Research indicated that the RTW rate for MHD patients in China is 15.7% (26/166), with only 33.6% (44/140) of patients expressing a willingness to RTW (3). Accurately assessing RTW's influencing factors is essential for improving employment rate. According to the Meleis' transition theory, RTW is essentially a role transfer of the patient, affected by a combination of personal factors (cultural beliefs, attitudes, knowledge, preparation, etc.), community factors (family, friends and health care staff support, etc.) and social factors (social culture, policy, etc.) (5, 6). Previous studies have identified various predictive factors for RTW in MHD patients, including male gender (7), younger age, absence of diabetes (8), and higher education levels (9). However, these studies typically focused on assessing the relationship between specific predictive factors and RTW, rather than conducting a comprehensive multi-factor analysis. As a result, they are unable to accurately predict the likelihood of a patient's RTW, which in turn affects the development and implementation of rehabilitation and RTW plans for patients.

Clinical predictive models can be broadly classified into two categories: diagnostic prediction models and prognostic prediction models. Diagnostic prediction models are based on cross-sectional data and employ multivariable analytic approaches to identify factors associated with the current status and estimate the probability that an individual is currently in a certain state, essentially serving as classification and screening tools for the present condition. In contrast, prognostic prediction models rely on longitudinal data to predict the probability of a future outcome event occurring in an individual (10).

Research has shown that effective risk assessment tools can be used to timely and accurately evaluate the likelihood of RTW for patients, thereby reducing unemployment rates (11). However, current research on RTW predictive models primarily focuses on patients with cancer (12), occupational injuries (13), and back pain (14), while studies on MHD patients remain scarce. Existing RTW predictive models are primarily based on linear regression, which has difficulty capturing nonlinear relationships between variables, limiting the predictive accuracy. Additionally, some of these models lack external validation, which may reduce their generalizability and applicability. Machine Learning (ML) is a technique that uses statistical models and algorithms to automatically extract features from labeled training data and build classifiers. Through learning and analyzing data, ML enables accurate classification and prediction of new, unseen data (15). Specifically, in handling nonlinear relationships, high-dimensional data, and complex interactions, ML offers superior predictive performance and greater flexibility than traditional logistic regression models (11). Yuan et al. found that in predicting the RTW of patients after cardiac rehabilitation, ML outperformed traditional logistic regression, with the Area Under the Curve (AUC) of ML being 0.787, significantly higher than the 0.744 of logistic regression (19). Therefore, predicting and assessing patients' RTW using ML holds significant application value due to its superior predictive performance. However, interpreting their internal decision-making processes remains challenging. Currently, ML-based RTW predictive models lack interpretability analysis and visual representation, which results in low clinical utility and limits their application in clinical practice (16, 17). Therefore, there is an urgent need to develop a machine learning-based tool for assessing RTW status and enabling risk stratification among young and middle-aged MHD patients.

In summary, this study aims to develop a simple and interpretable machine learning–based predictive model for young and middle-aged MHD patients to identify factors influencing current RTW status and enable risk stratification, thereby providing a practical tool for clinical screening and individualized rehabilitation decision-making.

## 2 Methods

### 2.1 Definition of return to work

According to Franche's definition, RTW is the process in which a patient exits the workforce due to illness and subsequently reintegrates into the labor market, by returning to their original position or commencing a new one (18). In this study, RTW was operationally defined as engagement in paid employment at the time of the survey, including both full-time and part-time work, regardless of whether individuals had returned to their original position or taken a new job. The outcome was treated as a binary variable (yes = employed at the time of the survey; no = not employed at the time of the survey). The positive class was defined as RTW. All model predictions represent the probability of having returned to work at the time of the survey, and all performance metrics were calculated and reported based on this positive class definition.

### 2.2 Participants and procedures

This multicenter cross-sectional study used convenience sampling to recruit MHD patients from hemodialysis centers of 8 tertiary hospitals in Guangdong, China during March 2023 to May 2024. The participants were allocated into two groups based on their RTW status: “RTW group” and “no RTW group”. The inclusion criteria: (i) Clinically diagnosed with ESRD (19); (ii) Undergoing hemodialysis treatment for ≥ 3 months; (iii) Males aged 18–60 years and females aged 18–55 years, based on China's current retirement age regulations; (iv) Employed prior to illness, with the job serving as the primary source of income; (v) Informed consent and voluntary participation. Exclusion criteria: (i) Patients undergoing other concurrent alternative treatments; (ii) Patients with severe dialysis complications, serious mental disorders, sensory impairments, or communication difficulties; (iii) Patients completely dependent on others for daily activities.

### 2.3 Sample size

This study included 31 baseline characteristic variables as research variables. The sample size for the modeling set was estimated using a commonly applied rule of thumb for machine learning–based predictive model development, namely that the sample size should be 10–20 times the number of candidate variables (20). After accounting for an anticipated 10% attrition rate, the required sample size for the modeling set was estimated to be 344–689 participants. The actual modeling set included 565 patients, which met this requirement. The sample size for the external validation set was estimated in advance using a precision-based approach for the concordance statistic (C-statistic), implemented via the powerROC tool (21). Based on previously reported validation results of RTW prediction models, the expected AUC was assumed to range from 0.82–0.90 (22–24), with an anticipated event rate of 50% and a target 95% confidence interval width of ≤ 0.10 (21). Under these assumptions, the required sample size was estimated to be approximately 158–275 participants. The final external validation cohort included 203 patients, which met this requirement.

### 2.4 Variables and instruments

According to Meleis' transition theory, influencing factors of RTW were identified from three levels: individual, family, and society. Through expert consultations and literature review, representative factors from each dimension were summarized. The final influencing factors included in each level are shown in Table 1.

**Table 1:** Included influencing factors.

Influencing factor category	Content
Individual level	
General demographic information	Gender, Age, Education level, Residence, Per capita monthly income, Medical insurance, Marital status
Disease-related information	Dialysis duration, Dialysis frequency, Dialysis time period, Chronic comorbidities, Long-term medication, Primary disease cause, Symptom burden
Work-related information	Job nature, Job type, Work mode
Laboratory indicators	Hemoglobin, Albumin, Creatinine, Urea, Phosphorus, Potassium, Calcium, Parathyroid hormone
Cultural beliefs and attitudes	Self-efficacy in RTW
Preparation and knowledge	Self-management, Social functioning
Family level	
Family function	Adaptation, Co-operation, Adulthood, Emotionality, Closeness of the family
Social level	
Social support	Objective support, Subjective support, Utilization of social support
Social isolation	Friend networks, Family networks

#### 2.4.1 General information survey

A self-designed general information survey was used, primarily covering demographic data, work-related information, disease-related data, and laboratory indicators for MHD patients.

#### 2.4.2 Return-to-work self-efficacy questionnaire (RTW-SE)

This questionnaire was developed by Lagerveld et al. (25). It aims to assess an individual's self-efficacy in RTW. The questionnaire has 11 items, with a 6-point Likert scale. A mean score higher than 4.5 indicates a higher self-efficacy for returning to work. In this study, the scale's Cronbach's α coefficient was 0.944. The mean score of this questionnaire was entered into the model as the RTW self-efficacy variable.

#### 2.4.3 Social support rating scale (SSRS)

This scale was developed by Shuiyuan and has 10 items (26). It aims to comprehensively assess social support from subjective support, objective support, and social support utilization. A higher score indicates a greater level of support an individual receives in their social life, while a total score of ≤ 31 is considered insufficient social support. The Cronbach's α coefficient of the SSRS was 0.755. The total score of this questionnaire was entered into the model as the social support variable.

#### 2.4.4 Social dysfunction screening scale (SDSS)

Developed by the World Health Organization (WHO), this scale assesses the social functioning of chronic disease patients (27). A total score of ≥2 indicates social dysfunction, with higher scores reflecting greater social dysfunction. Among young and middle-aged MHD patients, the test-retest reliability coefficient is 0.781 (27). The Cronbach's α coefficient of the SDSS was 0.722. The total score of this questionnaire was entered into the model as the social functioning variable.

#### 2.4.5 Lubben social network scale 6 (LSNS-6)

The LSNS-6, a simplified version of Lubben's social network scale, assesses social isolation according to the frequency and closeness of contact with family and friends (28). A total score of < 12 indicates social isolation. The Chinese version, validated by Chang et al. in 2018, shows high internal consistency, stable factor structure, and strong correlation with standard variables (29). The Cronbach's α coefficient was 0.740. The total score of this questionnaire was entered into the model as the social isolation variable.

#### 2.4.6 The Family Adaptation, Partnership, Growth, Affection, and Resolve Index (Family APGAR Index)

The original scale, developed by Smilkstein, is primarily used to evaluate family functioning in patients (30). The scale contains 5 items, with higher scores indicating better family support. The Chinese version of the Family APGAR Index has exhibited good internal consistency (α = 0.894) in hemodialysis patients (31). The Cronbach's α coefficient for this scale was 0.727. The total score of this questionnaire was entered into the model as the family functioning variable.

#### 2.4.7 Hemodialysis self-management instrument (HD-SMI)

This scale, developed by Yijun (32) and translated by Li et al. (33), was designed to measure the self-management level of MHD patients. It consists of 20 items, and the score is directly proportional to the self-management ability. The Cronbach's α coefficient for this scale was 0.797. The total score of this questionnaire was entered into the model as the self-management variable.

#### 2.4.8 Dialysis symptom index (DSI)

The DSI was first proposed by Weisbord et al. (34) and was revised by Yu et al. (35) to evaluate the frequency, severity and distress of symptoms in MHD patients. The scale contains 30 items, with higher scores indicating a greater symptom burden. The DSI had a Cronbach's α coefficient of 0.870. The total score of this questionnaire was entered into the model as the symptom burden variable.

The external validation dataset was collected using the same standardized questionnaire as the modeling set. All scale scoring procedures were kept consistent with those applied during the model development phase. The resulting dataset was subsequently used as an independent external validation set for evaluating the optimal model.

### 2.5 Ethical consideration

This study was approved by the ethics board of the associated hospitals (K-2022-089).

### 2.6 Data analysis

(i) Data sources and preprocessing: Data were obtained from MHD patients at eight tertiary hospitals in Guangdong Province between January 2023 and May 2024. Data from six centers in Guangzhou and Foshan served as the modeling set (*n* = 565), while data from two centers in Shenzhen served as the external validation set (*n* = 203). The two datasets did not overlap, and the external validation set was kept completely isolated throughout the model development process, being used only for final performance evaluation. In this study, only continuous laboratory variables had missing values, all with missing rates below 20%. These missing values were imputed using the median of the corresponding variable.

(ii) Variable description and analysis: Frequency and percentage were used to describe categorical data. Utilizing normal Q-Q plots, the normality of continuous data was evaluated. For normally distributed data, the mean ± standard deviation was used, while non-normally distributed data were described by the median and interquartile range. For the model building set, *t*-test was applied for normally distributed continuous data, Mann-Whitney *U*-test for non-normally distributed data, and chi-square test for categorical data. Standardized mean differences (SMD) were calculated for all variables to quantify between-cohort differences between the modeling and external validation set, with an SMD ≥ 0.20 considered to indicate a meaningful difference.

(iii) Classified multi-model comprehensive analysis: All candidate variables identified through univariate analysis were subsequently entered into a model comparison framework. Given that the predictive variables in this study encompassed psychosocial factors, demographic characteristics, and clinical indicators, complex nonlinear interactions and multicollinearity were likely to exist among variables. To adequately capture these data characteristics, five representative algorithms spanning major learning paradigms—including linear models, kernel-based methods, bagging, and boosting—were selected for systematic evaluation. These methods comprised logistic regression (LR), support vector machine (SVM), random forest (RF), extreme gradient boosting (XGBoost), and adaptive boosting (AdaBoost), aiming to assess the performance and applicability of models with varying levels of complexity in this dataset.

To prevent data leakage and obtain unbiased estimates of generalization performance, we employed 10-fold nested cross-validation for hyperparameter tuning and model evaluation. The outer loop consisted of 10-fold cross-validation, in which the original dataset was partitioned into 10 subsets; each subset in turn served as the outer validation set (i.e., outer test fold), with the remaining 9 subsets combined as the outer training set, and this procedure was repeated 10 times. The inner loop adopted 5-fold cross-validation, performed on each outer training set: the data were further divided into 5 subsets, with one subset serving as the inner validation set and the other 4 subsets as the inner training set in each iteration (36). To determine the optimal hyperparameters for each model, we performed grid search using the inner validation set, with the criterion of maximizing the area under the receiver operating characteristic curve (AUC). After the optimal hyperparameters were identified, the model was refitted on the outer training set using these parameters and subsequently applied to predict the outer validation set. This entire procedure was repeated 10 times, and the performance metrics obtained from the 10 outer validation sets were averaged, with 95% confidence intervals calculated, to serve as unbiased estimates of the models' generalization performance.

During this process, no additional feature selection was performed, and all models were trained and evaluated using the same set of candidate variables. The optimal model was determined comprehensively by comparing the models in terms of discriminative ability, calibration, and clinical utility. The receiver operating characteristic (ROC) curve was plotted to calculate the AUC value for each model, which was used to determine the model's predictive efficacy. Calibration curves were drawn to evaluate the consistency between predicted outcomes and the actual results. Additionally, decision curve analysis (DCA) was performed to assess the potential benefit of intervention treatment for patients. Precision–recall (PR) curves were also plotted to evaluate model performance with respect to positive-class prediction.

(iv) Feature selection: After identifying the optimal model through nested cross-validation, least absolute shrinkage and selection operator (LASSO) regression was further applied to perform dimensionality reduction on variables that were statistically significant in the univariate analysis. 10-fold cross-validation was used to select the optimal regularization parameter (λ). In this study, two commonly used criteria were compared. The lambda.min criterion selects the value of λ that minimizes the cross-validated error, but may retain a relatively larger number of variables. In contrast, the lambda 0.1se criterion selects the largest value of λ within one standard error of the minimum error, resulting in a more parsimonious model with improved simplicity and interpretability. To balance predictive performance and clinical utility, the optimal regularization parameter was ultimately determined according to the lambda 0.1se criterion, and variables with nonzero coefficients were selected as the core feature set for the final model.

(v) Optimal model training and validation: After determining the optimal algorithm via nested cross-validation and establishing the core feature set through LASSO regression, the final optimal model was retrained using the entire modeling dataset. Model performance was evaluated at two levels: internal and external validation. For internal validation, the performance metrics from the outer validation folds of the 10-fold nested cross-validation were averaged, providing an unbiased internal estimate of model performance on the modeling dataset. Learning curves were additionally plotted to assess model fit and stability, and to detect potential overfitting or underfitting. For external validation, the final trained optimal model was applied to a completely independent external validation set, with performance reported in terms of AUC, accuracy, sensitivity, specificity, positive predictive value, calibration curves, and DCA, serving as the ultimate assessment of true generalizability.

(vi) Model interpretation: SHapley Additive exPlanations (SHAP) was used to calculate the contribution of each feature to the prediction for each sample, represented by Shapley values. SHAP summary plots were constructed to rank the importance of risk factors, while individual SHAP explanation plots were generated to analyze the prediction results for each sample.

(vii) Model visualization: A web-based predictive model calculator was built using the Django framework, providing a platform for users to interact with the model.

(viii) Reproducibility: To ensure reproducibility, a fixed random seed of 42 was used for all analyses. Categorical variables were processed using one-hot encoding, and hyperparameters for all models were optimized via grid search. Detailed information regarding the software environment versions, final hyperparameter settings for each model, and main function code is provided in the Supplementary materials S1, S2.

## 3 Results

### 3.1 Patient characteristics

Seven hundred ninety-three patients from 8 tertiary hospitals were recruited from Mar 2023 to May 2024. Among them, 781 patients completed the survey, with 13 invalid questionnaires, resulting in a 98.3% valid response rate. Ultimately, 768 eligible patients were included. The patients' basic characteristics are demonstrated in Table 2. The study design and patient inclusion flowchart are shown in Figure 1.

**Table 2:** Basic characteristics of the included patients [*n* (%)].

Variable	Total (*n* = 768)	Modeling set (*n* = 565)	External validation set (*n* = 203)	Standardized mean difference
Gender				0.25
Male	514	396	118	
Female	254	169	85	
Age				0.16
18-44	252	179	73	
45-60	416	386	130	
Education level				0.41
Primary school or below	116 (15.10)	105 (18.58)	11 (5.42)	
Junior high school	279 (36.33)	224 (39.65)	55 (27.09)	
High school or vocational school	205 (26.69)	137 (24.25)	68 (33.50)	
Associate degree	120 (15.63)	72 (12.74)	48 (23.65)	
Bachelor's degree or above	48 (6.25)	27 (4.78)	21 (10.34)	
Monthly household income per capita (RMB)				0.18
≤ 2000	40 (5.21)	23 (4.07)	17 (8.37)	
2001–4000	268 (34.90)	220 (38.94)	48 (23.65)	
4001–6000	314 (40.89)	212 (37.52)	102 (50.25)	
>6000	146 (19.01)	110 (19.47)	36 (17.73)	
Marital status				0.008
Unmarried	118 (15.36)	91 (16.11)	27 (13.30)	
Married	612 (79.69)	446 (78.94)	166 (81.77)	
Divorced/Widowed	38 (4.95)	28 (4.96)	10 (4.93)	
Residence				0.57
Town	610 (79.43)	419 (74.16)	191 (94.09)	
Countryside	158 (20.57)	146 (25.84)	12 (5.91)	
Medical insurance				0.16
Self-paid or other	47 (6.12)	40 (7.08)	7 (3.45)	
Resident health insurance	372 (48.44)	226 (40.00)	146 (71.92)	
Employee medical insurance	349 (45.44)	299 (52.92)	50 (24.63)	
Dialysis duration (months)				0.15
3–6	133 (17.32)	106 (18.76)	27 (13.30)	
7–12	115 (14.97)	87 (15.40)	28 (13.79)	
13–60	328 (42.71)	246 (43.54)	82 (40.39)	
≥61	192 (25.00)	126 (22.30)	66 (32.51)	
Dialysis frequency (times/week)				0.17
< 3	77 (10.03)	64 (11.33)	13 (6.40)	
≥3	691 (89.97)	501 (88.67)	190 (93.60)	
Dialysis time period				0.20
Daytime	645 (83.98)	464 (82.12)	181 (89.16)	
Nighttime	123 (16.02)	101 (17.88)	22 (10.84)	
Number of comorbidities				0.08
1–2	247 (32.16)	187 (33.10)	60 (29.56)	
3–4	401 (52.21)	274 (48.50)	127 (62.56)	
≥5	120 (15.63)	104 (18.41)	16 (7.88)	
Number of long-term medications				0.44
1–2	200 (26.04)	120 (21.24)	80 (39.41)	
3–4	336 (43.75)	260 (46.02)	76 (37.44)	
≥5	232 (30.21)	185 (32.74)	47 (23.15)	
Primary cause of disease				0.53
Glomerulonephritis	116 (15.10)	55 (9.73)	61 (30.05)	
Diabetic nephropathy	170 (22.14)	131 (23.19)	39 (19.21)	
Hypertensive nephropathy	189 (24.61)	149 (26.37)	40 (19.70)	
Polycystic kidney disease	31 (4.04)	20 (3.54)	11 (5.42)	
Obstructive nephropathy	19 (2.47)	14 (2.48)	5 (2.46)	
Unknown cause	105 (13.67)	87 (15.40)	18 (8.87)	
Other	138 (17.97)	109 (19.29)	29 (14.29)	
Pre-disease job nature				0.09
Primarily intellectual work	189 (24.61)	133 (23.54)	56 (27.59)	
Primarily physical work	270 (35.16)	220 (38.94)	50 (24.63)	
Mixed intellectual and physical work	309 (40.23)	212 (37.52)	97 (47.78)	
Pre-disease job type				0.21
Government/enterprise staff	39 (5.08)	33 (5.84)	6 (2.96)	
Service industry worker	328 (42.71)	230 (40.71)	98 (48.28)	
Self-employed	207 (26.95)	162 (28.67)	45 (22.17)	
Worker	154 (20.05)	106 (18.76)	48 (23.65)	
Farmer or other	40 (5.21)	34 (6.02)	6 (2.96)	
Pre-disease work mode				0.55
Full-time	545 (70.96)	367 (64.96)	178 (87.68)	
Part-time/flexible work	223 (29.04)	198 (35.04)	25 (12.32)	
Pre-disease work nature				0.09
Intellectual work	189 (24.61)	133 (23.54)	56 (27.59)	
Physical work	270 (35.16)	220 (38.94)	50 (24.63)	
Mixed intellectual and physical work	309 (40.23)	212 (37.52)	97 (47.78)	
Return to work				/
No	459 (59.77)	353 (62.48)	106 (52.22)	
Yes	309 (40.23)	212(37.52)	97(47.78)	

**Figure 1:**
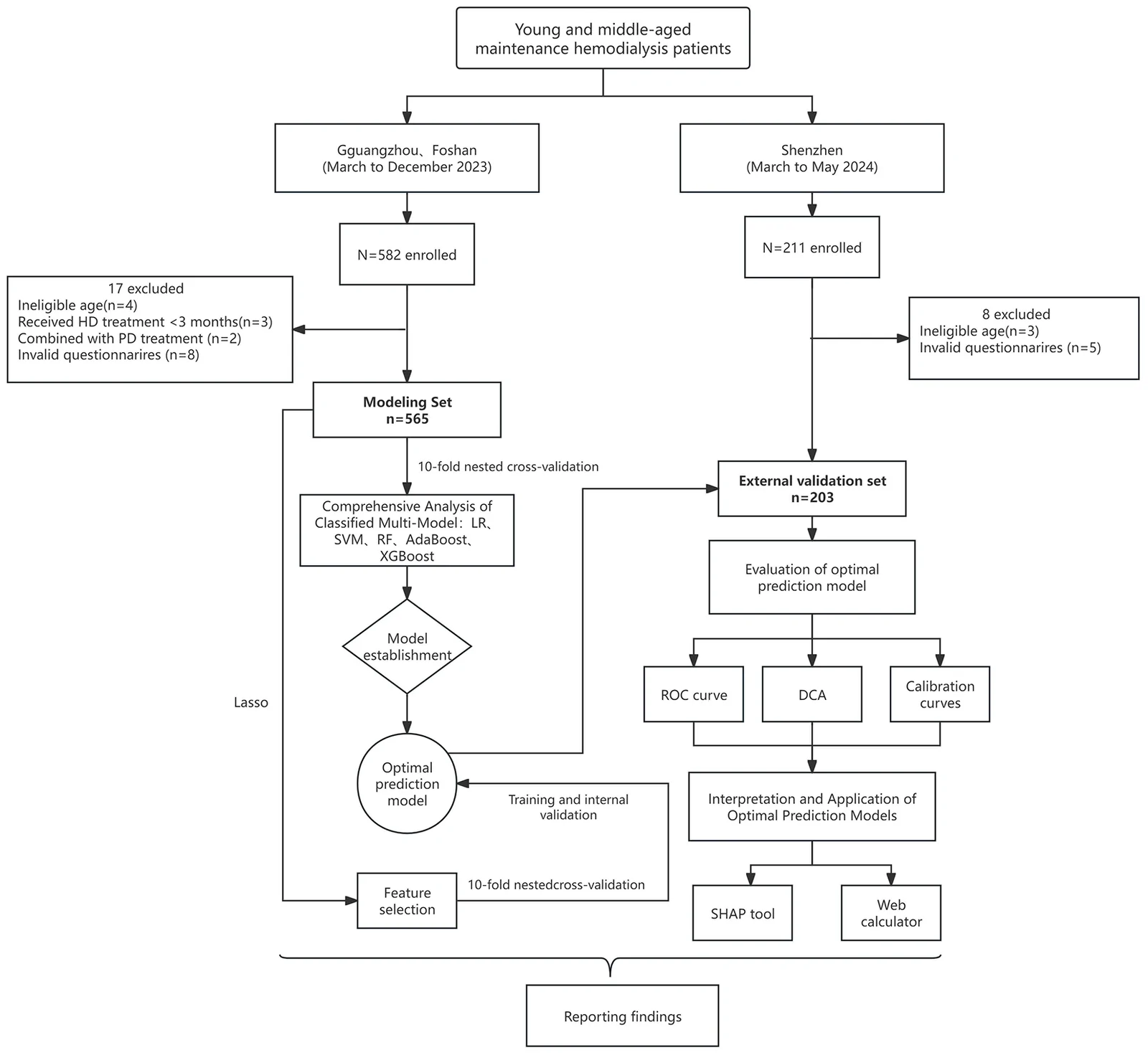
Flow diagram of patient selection and study design.

### 3.2 Univariate analysis results of RTW in the modeling set

Univariate analysis showed that gender, age, education level, household income, residence, medical insurance, dialysis frequency, dialysis time period, number of chronic comorbidities, medication count, primary disease cause, pre-disease job nature, pre-disease job type, RTW-SE, symptom burden, self-management, social functioning, family functioning, social support, social isolation, albumin, and creatinine were statistically significant (*P* < 0.05) for RTW.

### 3.3 Comprehensive analysis of classified multi-model

Based on 10-fold nested cross-validation, the five models achieved validation-set AUCs of 0.934 (95% CI 0.871–0.996) for LR, 0.941 (95% CI 0.881–0.998) for XGBoost, 0.946 (95% CI 0.892–0.997) for RF, 0.941 (95% CI 0.883–0.998) for AdaBoost, and 0.944 (95% CI 0.886–0.998) for SVM. Details are provided in Table 3 and Figures 2a, b. RF achieved the highest AUC among all models. The calibration curve showed good agreement between predicted probabilities and observed outcomes (Figure 2c), while DCA demonstrated that RF yielded a net clinical benefit across threshold probabilities ranging from 1 to 99%, with a maximum net benefit of 0.382 (Figure 2d). In the precision–recall analysis of the validation set, RF achieved the highest AP on both the training and validation sets, with an AP of 0.992 (95% CI 0.987–0.996) and 0.906 (95% CI 0.884–0.928), respectively (Figures 2e, f). Considering its discriminative ability, calibration performance, and clinical utility, the RF model was identified as the optimal predictive model.

**Table 3:** Performance comparison of the machine learning models using 10-fold nested cross-validation.

Model	Dataset	AUC (95% CI)	Accuracy	Sensitivity	Specificity	Precision	F1 score
LR	Training	0.950 (0.932–0.968)	0.890	0.912	0.875	0.815	0.862
	Validation	0.934 (0.871–0.996)	0.849	0.859	0.844	0.785	0.813
SVM	Training	0.985 (0.977–0.994)	0.948	0.932	0.958	0.931	0.931
	Validation	0.944 (0.886–0.998)	0.88	0.845	0.901	0.85	0.842
RF	Training	0.996 (0.991–1.000)	0.985	0.977	0.991	0.984	0.981
	Validation	0.946 (0.892–0.997)	0.865	0.802	0.904	0.843	0.817
XGBoost	Training	0.996 (0.990–1.000)	0.985	0.97	0.994	0.99	0.980
	Validation	0.941 (0.881–0.998)	0.842	0.666	0.949	0.901	0.758
AdaBoost	Training	0.970 (0.959–0.982)	0.907	0.92	0.899	0.847	0.881
	Validation	0.941 (0.883–0.998)	0.841	0.82	0.853	0.79	0.795

LR, logistic regression; SVM, support vector machine; RF, random forest; XGBoost, extreme gradient boosting; AdaBoost, adaptive boosting; AUC, the area under curve; CI, confidence interval.

**Figure 2:**
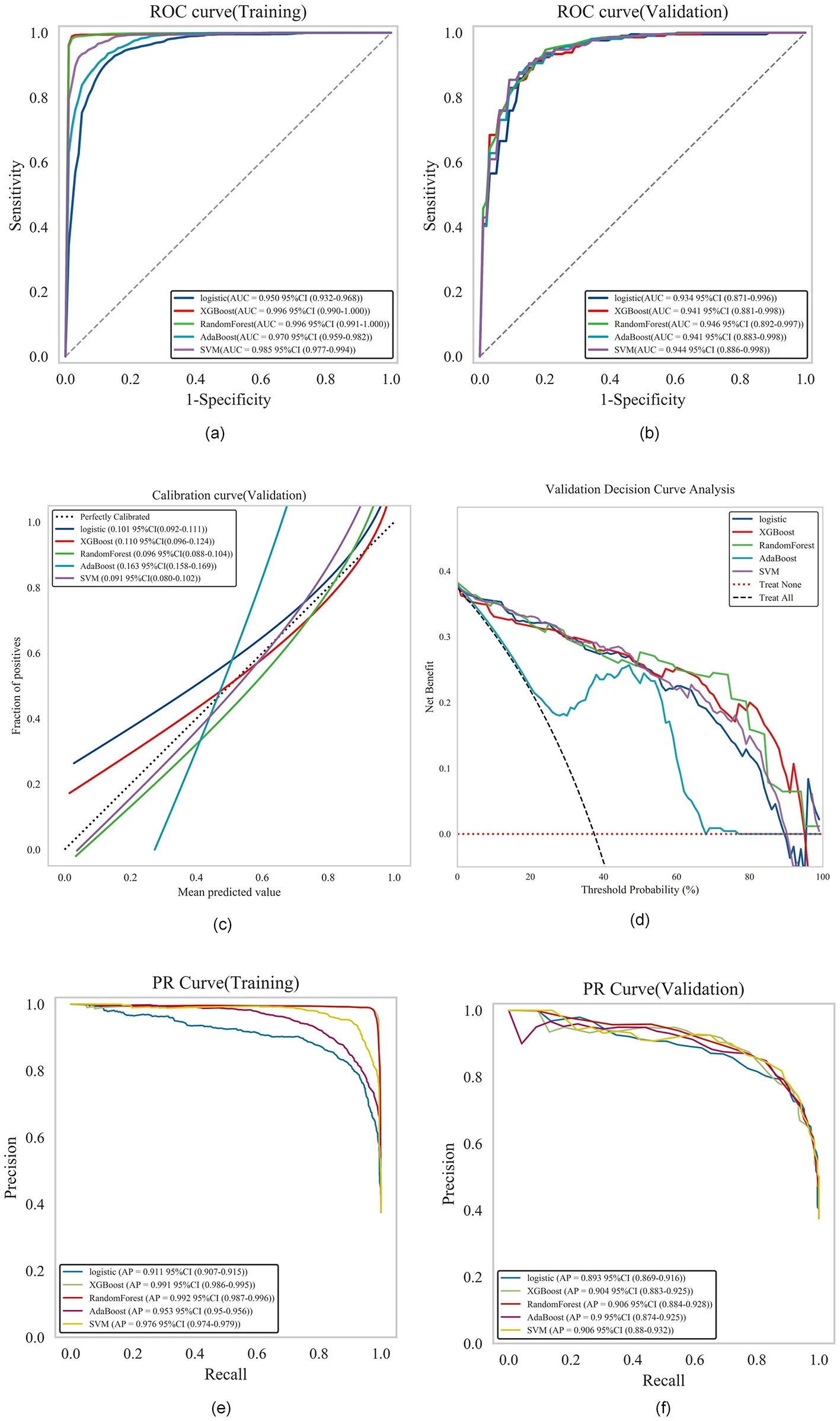
**(a)** ROC curves of the five models in the training set. **(b)** ROC curves of thefive models in the validation set. **(c)** Calibration plots of the five models. The 45° dottedline on each graph represents the perfect match between the observed (y-axis) andpredicted (x-axis) RTW probabilities. A closer distance between two curves indicatesgreater accuracy. **(d)** DCA curves of the five models. The red dottedline represents “no intervention for all patients”, and the black dotted line represents “intervention for all patients”. The colorful solid lines represent different models, andthe range of their intersections with the red and black dotted lines indicates the rangeof patients who could benefit. **(e)** Precision-Recall (PR) curves of the five models in the training set. **(f)** Precision-Recall (PR) curves of the five models in the validation set. If the PR curve of one model is completely covered by the PR curve of another model, it can be concluded that the latter is better than the former, and the higher the AP value, the better the model perform.

### 3.4 Screening results for characteristic variables

After RF was identified as the optimal model, we further applied LASSO regression to reduce the dimensionality of the 22 candidate variables that were statistically significant in the univariate analysis. 10-fold cross-validation was performed, the optimal regularization parameter was determined according to the lambda 0.1se criterion (λ = 0.036). At this penalty level, LASSO regression selected 10 variables with non-zero coefficients from the 22 candidate variables, including gender, age, residence, chronic comorbidities, dialysis time period, RTW-SE, family functioning, social support, social isolation, and social functioning (Figure 3).

**Figure 3:**
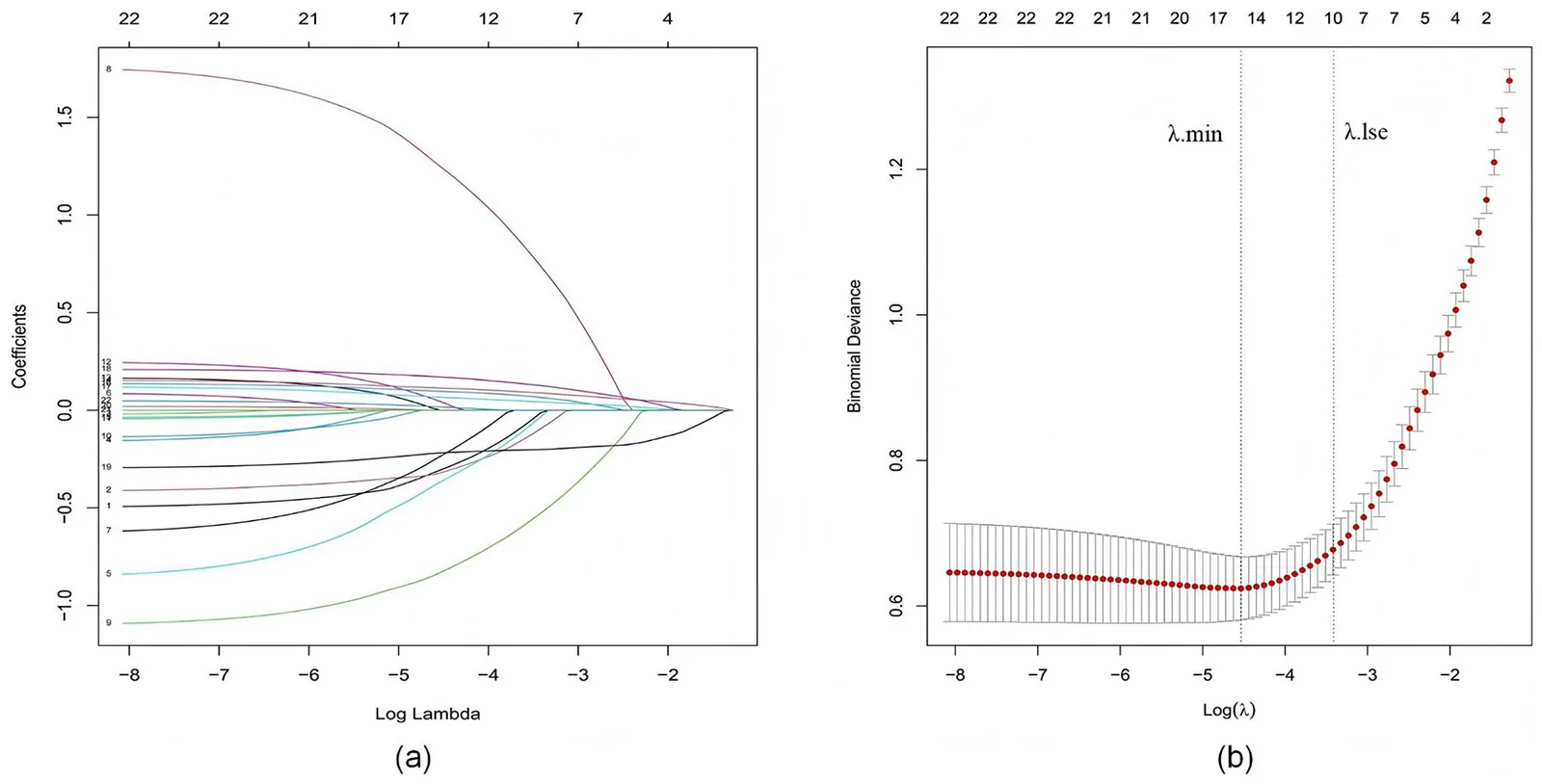
Feature selection based on LASSO regression. **(a)** LASSO coefficient plot. **(b)** LASSO cross-validation results showing the process of selecting the optimal number of variables. λ.min represents the λ value corresponding to the minimum mean squared error, while λ.lse represents the λ value corresponding to the point within one standard error of the minimum MSE. The optimal λ value is selected as λ.lse = 0.036, with 10 non-zero coefficient features selected.

### 3.5 The optimal model building and evaluation

The RF model was retrained on the modeling set (*n* = 565) using the 10 selected predictors, with hyperparameters optimized via 10-fold nested cross-validation.

#### 3.5.1 Internal validation

Based on 10-fold nested cross-validation, the RF model achieved an internal validation AUC of 0.939 (0.916–0.962) in the modeling set, with an accuracy of 0.874, sensitivity of 0.829, and specificity of 0.901. Details are provided in Table 4 and Figure 4a. The learning curve showed that both the training and validation scores gradually plateaued as the training sample size increased, with a final gap of approximately 0.064. These results indicate good model fit, with no apparent evidence of overfitting or underfitting (Figure 4b).

**Table 4:** Performance of the random forest model in internal and external validation.

Performance metrics	Internal validation	External validation
AUC (95% CI)	0.939 (0.916–0.962)	0.918 (0.873–0.963)
Cut off	0.530	0.500
Accuracy	0.874	0.901
Sensitivity	0.829	0.928
Specificity	0.901	0.877
Precision	0.847	0.874
F1 score	0.833	0.900

**Figure 4:**
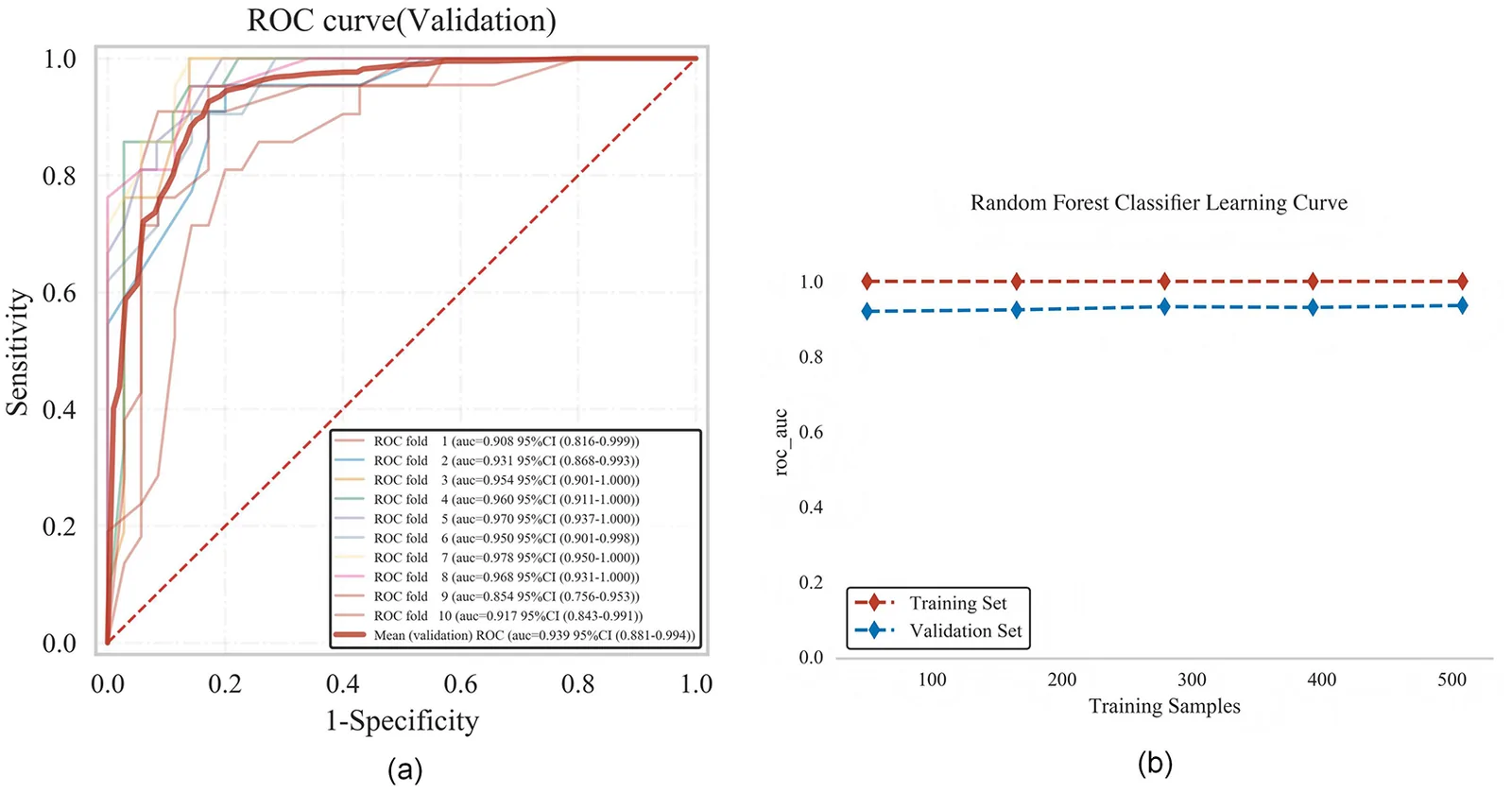
**(a)** The ROC curve of the random forest model in internal validation. **(b)** Learning curve of the random forest model.

#### 3.5.2 External validation

The RF model was further evaluated in an independent external validation set (*n* = 203). The model achieved an AUC of 0.918 (95% CI 0.87–0.963), an accuracy of 0.901, a sensitivity of 0.928, and a specificity of 0.877. Details are provided in Table 4 and Figure 5a. The calibration curve demonstrated good agreement between predicted and observed outcomes, with a Brier score of 0.088 (95% CI 0.061–0.121) (Figure 5b). Decision curve analysis indicated that the model yielded a positive net clinical benefit across threshold probabilities ranging from 10 to 80% (Figure 5c). Collectively, these results demonstrate that the RF model exhibited good discrimination, calibration, and generalizability in an independent external population.

**Figure 5:**
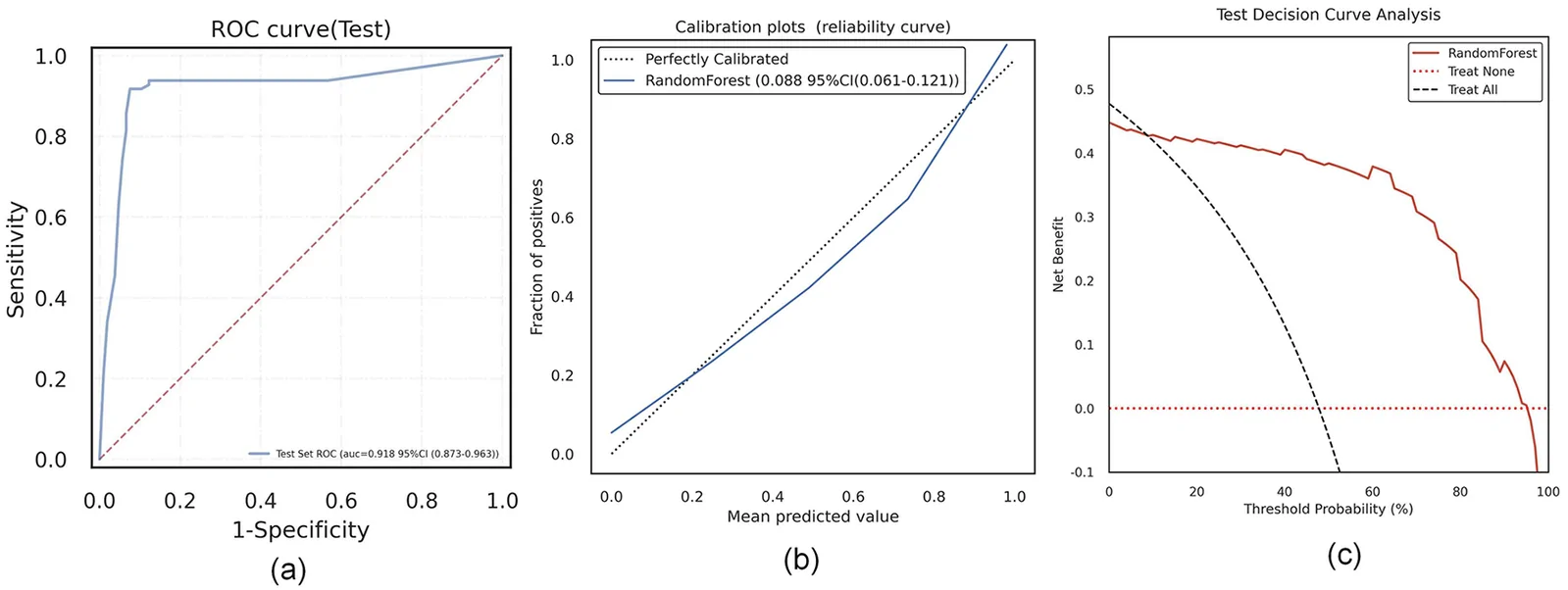
**(a)** ROC curve of the random forest model for the external validation set. **(b)** Calibration curve of the random forest model for the external validation set. **(c)** DCA curve of the random forest model for the external validation set.

### 3.6 Model explanation

As shown in Figure 6a, the factors affecting RTW are ranked from largest to smallest as follows: RTW-SE, social functioning, chronic comorbidities, social support, social isolation, family functioning, age, dialysis time period, residence and gender. The larger the eigenvalue of RTW-SE, the larger the SHAP value, indicating that it has a positive impact on patients‘ RTW. The effects of social isolation, social support, family functioning, and nocturnal dialysis on the prediction results followed the same pattern. The larger the eigenvalue of social function impairment, the smaller the SHAP value, indicating a negative impact on patients' RTW. Additionally, as the eigenvalues of chronic comorbidities, age, residence, and gender increase, the SHAP value decreases. This indicates that having more chronic comorbidities, being over 45 years old, living in rural areas, and being female have a negative impact on RTW.

**Figure 6:**
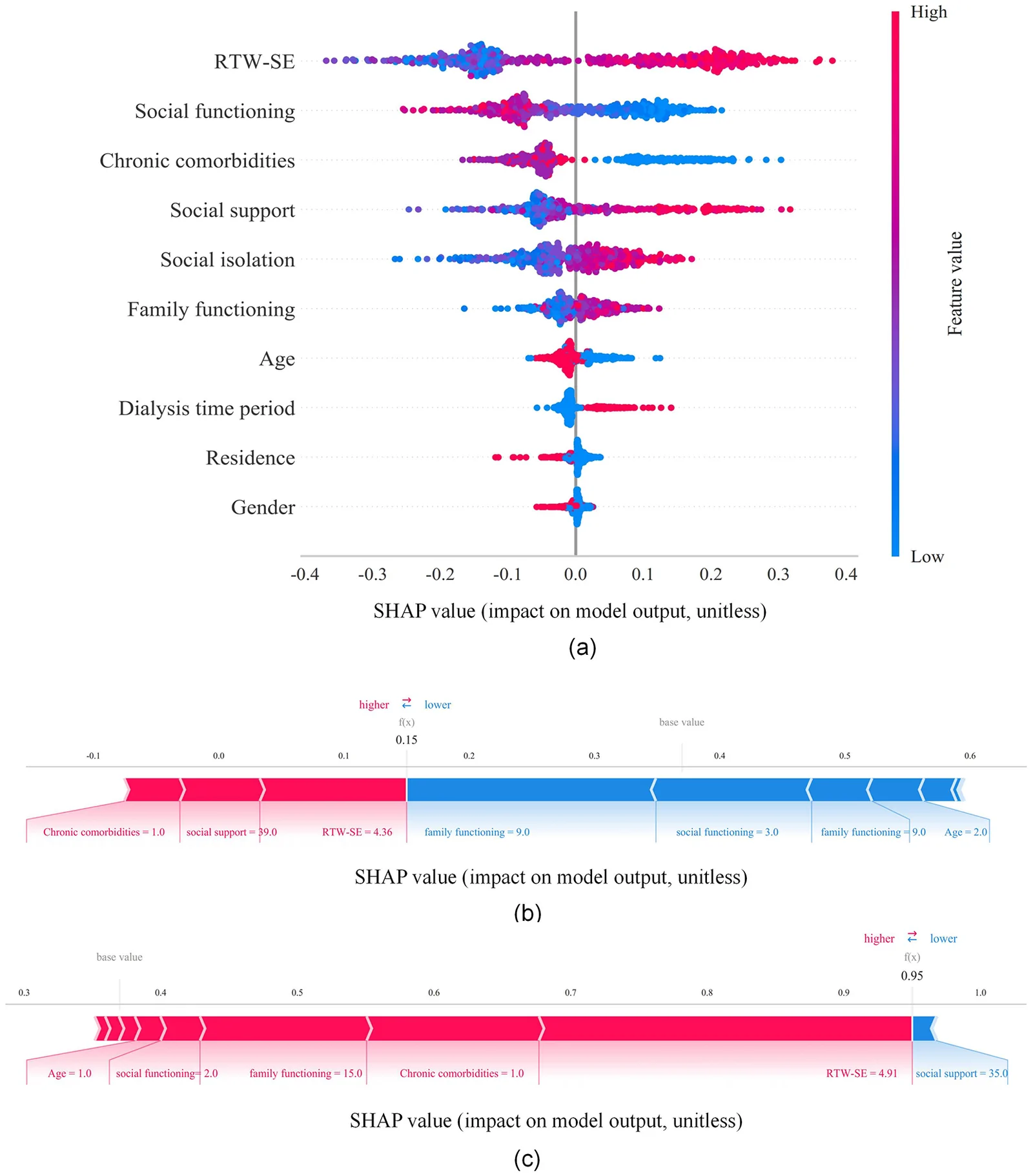
**(a)** SHAP summary plot. The vertical axis represents the importance ranking of each feature variable on RTW impact, while the horizontal axis shows the SHAP values, indicating the extent of each feature's influence on RTW. A positive SHAP value indicates a positive impact, while a negative SHAP value indicates a negative impact. Each point in the plot represents a feature of a sample, with redder points indicating higher feature values and bluer points indicating lower feature values. **(b)** SHAP force plot for predicting patient I. **(c)** SHAP force plot for predicting patient II. Blue variables represent a decrease in the probability of RTW, with a reduction in SHAP values. Red variables represent an increase in the probability of RTW, with an increase in SHAP values.

To illustrate the interpretability of the model, we provided SHAP summary plots for two representative patients: one patient had a low predicted probability (0.15), suggesting that the patient was more likely to be in a non-RTW state (Figure 6b); the other had a high predicted probability (0.95), suggesting that the patient was more likely to be in an RTW state (Figure 6c).

### 3.7 Application of the model

As shown in Figure 7, the final prediction model has been integrated into a web-based application for clinical use. After entering the required predictor variables, the application outputs the predicted probability that the patient has currently RTW. The web application is accessible online at: https://www.xsmartanalysis.com/predictive/preview/140.

**Figure 7:**
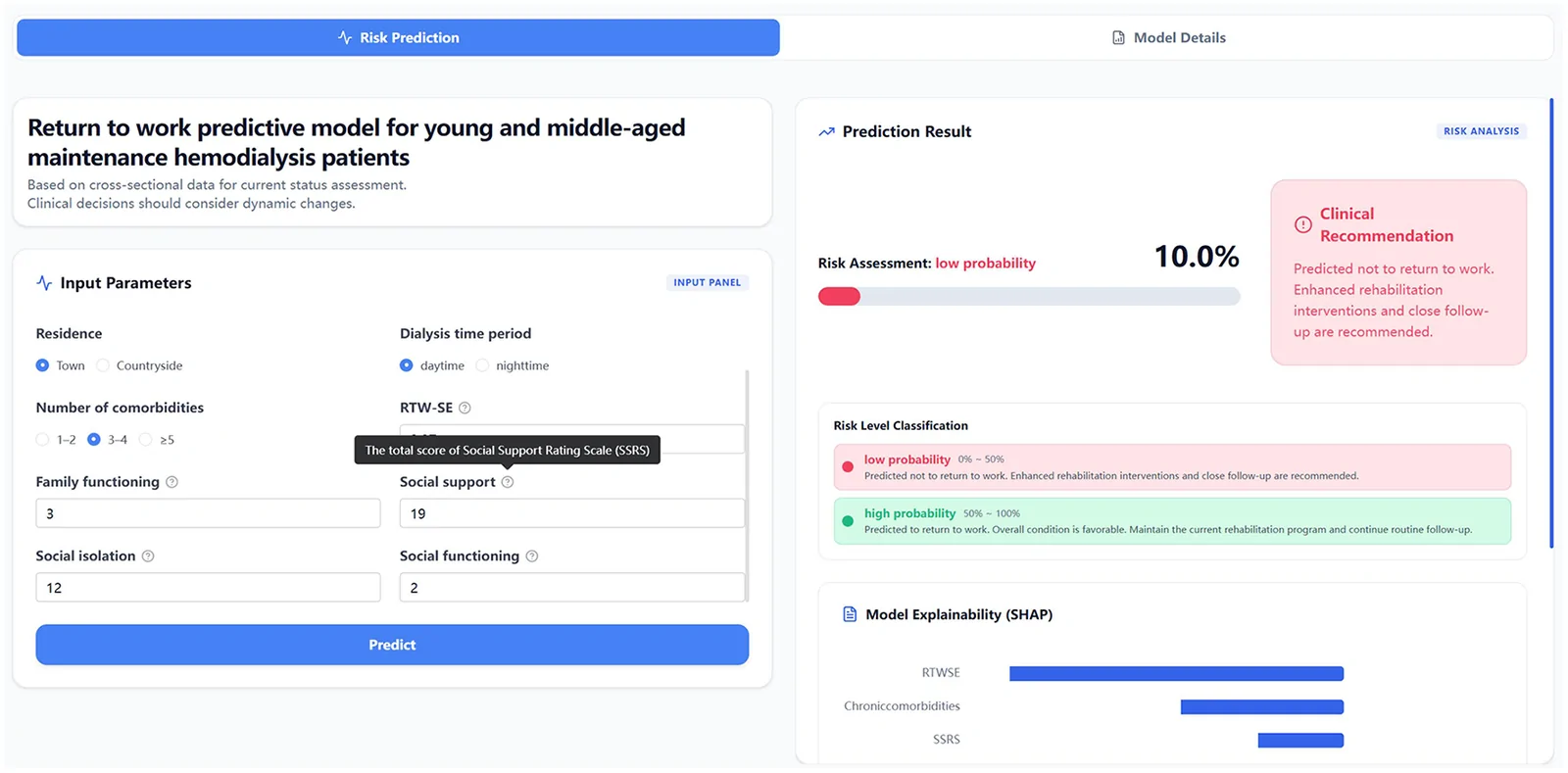
Web-based application interface of the return-to-work prediction model for young and middle-aged maintenance hemodialysis patients. The interface consists of three sections: input parameters **(left panel)**, prediction results **(middle panel)**, and model explanation **(bottom panel)**. After users input the predictor variables, the model outputs the predicted probability of return to work along with clinical recommendations.

Based on the Youden Index, the optimal classification threshold for the RF model was determined to be 0.5. When the predicted probability is below 0.5, the patient is classified into the “non-RTW” group, indicating a low likelihood of current RTW, which may alert clinicians to provide timely attention and develop individualized rehabilitation support plans. When the predicted probability is above 0.5, the patient is classified into the “RTW” group, indicating a high likelihood of current RTW.

## 4 Discussion

A core goal of MHD treatment is to maintain the patient's socialization and socioeconomic status (37). RTW is crucial for patients' livelihood, representing an important step in improving their quality of life and subjective wellbeing, and achieving and maintaining socialization and socioeconomic status. The RTW rate for young and middle-aged MHD patients in this study was 40.23%, suggesting that the post-treatment employment status of this population is not optimistic. However, there is currently no dedicated screening instrument for assessing RTW status among young and middle-aged MHD patients. Therefore, this study aimed to develop and validate a machine learning-based predictive model for identifying influencing factors of current RTW status and enabling risk stratification among young and middle-aged MHD patients, thereby providing a practical screening tool for clinical rehabilitation assessment.

Previous studies have shown that machine learning methods achieve high accuracy in predicting return-to-work outcomes (13, 38), and that ensemble learning methods generally outperform single linear models in structured data settings and in scenarios involving complex interactions (39, 40). Since RTW involves multiple overlapping factors, including the patient's perspective and the social cost perspective, ML approaches are well suited to capturing these multidimensional relationships and may facilitate the development of individualized rehabilitation management strategies. To assess the applicability of various machine learning algorithms to the present dataset, we performed a comprehensive comparative analysis of five algorithmic models. In the comparison of the five models, the RF model achieved the highest AUC on the validation set (0.946), slightly outperforming SVM (0.944), XGBoost (0.941), AdaBoost (0.941), and LR (0.934). The AUC values of all models were generally similar, indicating that multiple algorithms demonstrated good discriminative ability for this prediction task. In the precision-recall analysis, RF and SVM achieved the highest AP values, both at 0.906, suggesting that the two models performed comparably in identifying positive-class samples.

Considering the data characteristics of this study, RF demonstrated superior algorithmic applicability. The predictors in this study encompassed multiple types of variables, including psychosocial factors, demographic characteristics, and clinical indicators, which may involve complex non-linear interactions. LR assumes a linear relationship between predictors and the outcome, as well as mutual independence among predictors, which limits its ability to effectively capture the aforementioned interaction effects (41). Although SVM can accommodate certain nonlinear relationships, its performance in settings with highly heterogeneous variable types depends heavily on kernel selection and parameter tuning, and their interpretability remains limited (42). In contrast, as an ensemble learning method based on decision trees, RF is inherently capable of handling nonlinear relationships and higher-order interactions among variables without requiring any predefined functional form. It is also robust to outliers and insensitive to feature scaling, making it particularly well-suited for heterogeneous data types (43, 44). By integrating the advantages of ensemble learning and decision tree models, RF can effectively capture complex data structures and uncertainty commonly observed in medical datasets. These properties may partly explain its superior performance compared with other models in this study. Similarly, a study by Jongin Lee et al. involving patients with occupational injuries also found that the RF model achieved the best performance (AUC = 0.811, accuracy = 0.786) (38). Van Deynse et al. further recommended RF as the optimal predictive tool for RTW among patients with traumatic brain injury (45), which is consistent with the findings of this study. Taken together, based on model performance comparisons, algorithmic characteristics, and evidence from previous literature, RF was identified as the optimal predictive model.

Following the identification of the RF model as the optimal predictive model, LASSO regression was employed to perform dimensionality reduction on the 22 candidate variables. Two selection criteria, lambda.min and lambda.0.1se, were evaluated during the feature selection process. The lambda.min criterion (λ = 0.011) retained 14 predictors with nonzero coefficients, whereas the lambda.1se criterion (λ = 0.027) resulted in a more parsimonious model with 10 predictors, offering improved simplicity and potential clinical applicability.

Transitioning from lambda.min to lambda.1se excluded four variables: dialysis frequency, Pre-disease job nature, serum creatinine, and serum albumin. Of these, serum creatinine and serum albumin require laboratory testing, which may increase the burden of data collection, while dialysis frequency and type of occupation are less well-established as predictors of RTW outcomes. Notably, all major psychosocial predictors, such as RTW-SE, family functioning, and social support, were retained in the reduced model. As concise screening tools are more likely to be adopted in routine clinical settings, the lambda 0.1se model may provide superior clinical utility while maintaining adequate predictive capability. Considering that a more concise tool is more conducive to clinical implementation, the model based on the lambda 0.1se criterion was ultimately selected as the final set of predictors. The ten variables retained in the final model were as follows: RTW-SE, social isolation, family functioning, social functioning, social support, age, gender, residence, chronic comorbidities and dialysis time period. The associations between each of the above 10 variables and RTW status in young and middle-aged MHD patients are discussed below.

This study found that RTW-SE is the most significant predictor of patients' RTW, showing that the higher the patients' RTW-SE, the greater the probability of their RTW. This is consistent with previous studies (46–48). When patients have higher RTW-SE, they are more confident in performing work tasks. This confidence encourages them to actively seek RTW opportunities and develop clear work plans, making it more likely for them to achieve successful RTW. Conversely, when patients have lower RTW-SE, they often experience self-doubt, anxiety, and fear about RTW, which can lead to work withdrawal behaviors (49). Healthcare professionals should focus on patients‘ RTW-SE levels, provide more information on disease prognosis and RTW guidance, and share successful RTW cases to inspire patients' confidence and enthusiasm, ensuring a smooth transition into RTW role.

Greater social isolation in patients is associated with a lower likelihood of RTW. Patients are more likely to experience social anxiety, social avoidance, and other issues during interpersonal interactions, which hinder the formation and maintenance of social connections. This leads to their gradual marginalization from social circles and the onset of social isolation, causing the shrinkage of their social networks (50). This may limit the patient's ability to access social support, information, and resources, leaving them feeling helpless and frustrated when facing the challenges and pressures of RTW, further undermining their confidence and motivation, thereby affecting the progress of RTW (51). Therefore, healthcare professionals should focus on addressing and alleviating the patient's sense of social isolation. This can be done by helping them rebuild their social networks through establishing support networks and encouraging participation in social activities, thereby creating better conditions for the patient's RTW.

Better family functioning is associated with a higher likelihood of successful RTW, aligning with the findings of Huang et al. (3). Improved family functioning enhances patients' sense of familial support, facilitating early RTW and fostering a positive work-family relationship (52). Therefore, healthcare professionals can strengthen the support and role of family function by conducting home visits, providing family care guidance, and organizing family support groups to create a better rehabilitation environment for the patient.

Greater social functional impairment is associated with a lower likelihood of RTW. Due to changes in physiological function and partial loss of physical capabilities, patients may exhibit negative attitudes in social interactions, suppressing their behaviors and expressions. This can lead to abnormal social roles and social functional impairments (53), which not only hinder the patient's smooth reintegration into family and work but also result in the development of negative values and social withdrawal behaviors (54). Therefore, healthcare professionals should develop dynamic care strategies for patients with social functional impairments. For example, designing specific social skills training and role reconstruction programs, using methods such as role-playing and scenario simulations, these measure can help patients gradually regain their social abilities and redefine their roles within the family and society.

Good social support positively promotes the patient's RTW, aligning with previous findings (55, 56). Effective RTW after sick leave is associated with a broad support system (57), such as workplace support and healthcare professionals' support (58). Therefore, healthcare professionals should increase their support for patients and assist them in establishing support systems.

The age range of 45–60 is a negative predictor for RTW, consistent with most studies (2, 59). With increasing age, physical decline and the high demands of dialysis make middle-aged and older adults more prone to reductions in physical strength, endurance, and cognitive abilities, affecting their RTW capacity. Male patients have a higher likelihood of RTW compared to females, as men are generally more rational and receive more social support, whereas women tend to be more psychologically sensitive, with heightened awareness of dialysis side effects, making them more cautious about RTW (2, 7). Patients in urban areas have a higher likelihood of RTW, benefiting from better social support systems and abundant employment opportunities (60), while rural patients face greater challenges due to limited medical resources, fewer job opportunities, and weaker social support (61). Additionally, the more chronic comorbidities a patient has, the lower their chances of RTW, as these conditions not only impair health and work ability but also cause symptoms like pain and fatigue, diminishing RTW confidence and motivation (62). This study also found that nighttime dialysis had a positive impact on RTW among young and middle-aged MHD patients. Compared with daytime dialysis, nighttime dialysis offers patients greater flexibility in scheduling, avoids conflicts with work hours, and helps them better balance treatment and employment (63). Therefore, healthcare professionals should develop personalized rehabilitation strategies based on age, gender, living environment, and comorbidities. They can promote nocturnal dialysis, as its flexible scheduling helps balance work and treatment, supporting patients in achieving a harmonious work-life balance.

Based on the above ten predictor variables, the RF model was retrained, and both internal and external validations were performed to evaluate its predictive performance and generalizability. The results of internal validation demonstrated good predictive performance in the modeling set. Furthermore, external validation in an independent dataset further confirmed its generalizability to unseen samples.

The AUC of the RF model decreased from 0.939 in the internal validation to 0.918 in the external validation. To explore potential reasons for this decline, we further compared the characteristics of the derivation and external validation datasets. The SMD analysis revealed substantial differences between the modeling set and the external validation set in place of residence (SMD = 0.57), pre-disease work mode (SMD = 0.55) and primary cause of disease (SMD = 0.53), as well as small-to-moderate differences in educational level (SMD = 0.41), number of long-term medications (SMD = 0.40), and gender (SMD = 0.25). Such population heterogeneity may partly account for the observed decrease in AUC. In addition, as the external validation data were derived from two other medical institutions, differences in patients' socioeconomic status, availability of rehabilitation resources, and dialysis management practices may have also contributed to the observed performance differences. Nevertheless, the AUC in the external validation remained at 0.918, indicating that the model has good generalizability across populations. Future studies may further evaluate its generalizability by incorporating more diverse external datasets.

From a clinical applicability perspective, this model requires only 10 readily available predictors to achieve accurate assessment of RTW status among young and middle-aged MHD patients. This feature suggests that the model also has potential for implementation in primary healthcare settings. Taken together with its robust internal validation performance, stable external validation results, and straightforward clinical implementation process, the model may serve as an effective screening tool to assist clinicians in identifying MHD patients at high risk of not returning to work, thereby providing decision support for the development of individualized rehabilitation strategies. Although the present study employed a cross-sectional design and the model therefore reflects associations between variables and current RTW status rather than causal relationships or prospective prediction, it still retains clear clinical value as a bedside screening tool. By enabling rapid identification of patients who are currently unemployed, it may facilitate timely psychosocial interventions and risk stratification, as well as support the rational allocation of clinical resources.

To improve the clinical interpretability of the model, we applied SHAP to visualize the predictions, showing the direction and magnitude of each feature's contribution to individual risk estimates. This visualization supports clinicians in understanding the rationale behind the model's decisions. Furthermore, we developed an online risk assessment tool based on the RF model, which enables convenient estimation of the predicted probability of RTW and offers good accessibility and usability. All predictors in this tool can be readily obtained through patient self-report or standardized questionnaires, without requiring laboratory measurements, thus further reducing the technical barriers to clinical adoption.

Several limitations exist in this study. First, as the sample was limited to Chinese patients, the generalizability of the findings requires further validation across different cultural settings. In addition, we did not assess the predictive fairness of the model across demographic subgroups, and future research should address this aspect. Second, the use of self-report to assess RTW may introduce recall bias. Future research should strengthen interdisciplinary collaboration and consider using multiple assessment methods. Third, the external validation data were obtained exclusively from two tertiary hospitals in Shenzhen, with limited sample sizes at each center, making it difficult to perform stable site-specific performance evaluation for each center separately. Future studies should include larger multicenter external validation datasets to enable more robust center-stratified performance assessment. Fourth, as a cross-sectional study, it cannot establish causal relationships between RTW and risk factors, limiting its predictive value. Further research should enlarge sample size and conduct cohort research to establish causal links between risk factors and RTW, enhancing the evidence strength of the findings.

## 5 Concluding remarks

In conclusion, the RF predictive model developed in this study demonstrated good performance in identifying young and middle-aged MHD patients with a current non-RTW status and their key influencing factors, while enabling risk stratification. As a practical screening tool, the model may assist clinicians in identifying of patients requiring targeted psychosocial support and rehabilitation resources, thereby facilitating precision rehabilitation assessment and stratified management. Future prospective studies are needed to further validate the generalizability of the model and to investigate whether rehabilitation support programs developed based on the identified factors can effectively improve RTW outcomes.

## Data Availability

The raw data supporting the conclusions of this article will be made available by the authors, without undue reservation.
