# Supplementary material for: Development and validation of a machine learning-based return to work predictive model for young and middle-aged maintenance hemodialysis patients
Source: Front Public Health. 2026 Sep 24;14:1846282. doi: 10.3389/fpubh.2026.1846282 (PMC13645766; doi:10.3389/fpubh.2026.1846282)
Supplement: Supplementary file 1 [file Supplementary_file_1.docx]

**Supplementary Material S1**

**Software Environment, Hyperparameter Configurations, and Replication Details**

This supplementary material provides detailed information on the software environment, hyperparameter configurations, and replication details for the machine learning models developed in this study.

### **1. Software Environment**

All analyses were conducted in the following software environments:

**Python environment (version 3.11.9):**

| **Package** | **Version** |
| --- | --- |
| pandas | 2.3.3 |
| numpy | 1.26.4 |
| scikit-learn | 1.8.0 |
| matplotlib | 3.8.0 |
| scipy | 1.11.4 |
| seaborn | 0.13.2 |
| xgboost | 3.0.2 |
| lightgbm | 3.2.1 |
| joblib | 1.5.3 |
| catboost | 1.2.10 |
| ngboost | 0.5.8 |
| yellowbrick | 1.5 |
| shap | 0.43.0 |
| statsmodels | 0.14.6 |

**R environment (version 4.3.3):**

| **Package** | **Version** |
| --- | --- |
| glmnet | 5.0 |
| survival | 3.8.6 |

**SPSS: Version 25.0**

2. **Hyperparameter Configurations**

| **Model** | **Hyperparameters** |
| --- | --- |
| Logistic Regression | C = 1.0; solver = lbfgs; max_iter = 100; tol = 0.0001 |
| XGBoost | objective = binary:logistic |
| Random Forest | n_estimators = 20; max_features = 'sqrt'; min_samples_split = 2; min_samples_leaf = 1; criterion = 'gini'; random_state = 42 |
| AdaBoost | n_estimators = 50; learning_rate = 1.0 |
| SVM | C = 1.0; kernel = 'rbf'; gamma = 'scale'; probability = True; tol = 0.001 |

**3. Replication Details**

3.1 Random seed: All stochastic processes were fixed with random seed = 42.

3.2 Categorical variable encoding: All categorical variables were encoded using one-hot encoding prior to model training.

3.3 Missing data imputation: Continuous laboratory variables with missing rates below 20% were imputed using the median of the derivation cohort; imputation parameters were calculated based on the derivation cohort and applied to the external validation cohort.

3.4 Hyperparameter tuning: Grid search was employed within the inner loop of nested cross-validation, with inner-loop validation AUC maximization as the selection criterion.

3.5 LASSO feature selection: Performed using the glmnet package in R with 10-fold cross-validation; the lambda.1se criterion was applied to determine the optimal regularization parameter.
