# Supplementary material for: Development and validation of a machine learning-based return to work predictive model for young and middle-aged maintenance hemodialysis patients
Source: Front Public Health. 2026 Sep 24;14:1846282. doi: 10.3389/fpubh.2026.1846282 (PMC13645766; doi:10.3389/fpubh.2026.1846282)
Supplement: Supplementary file 3 [file Supplementary_file_3.docx]

**Supplementary Material S3**

**Main function code**

\# 1. 缺失值处理：中位数填补

import datetime

import os

from core.utils.data_filling_plot import distribution_curve

from core.utils.feature_classification import feature_classification

from core.utils.get_environment import get_env_info

\# 解耦导入

from core.utils.summarize_features import summarize_features as describe

current_time = datetime.datetime.now().strftime("%Y-%m-%d_%H-%M-%S")

import matplotlib

import numpy as np

import pandas as pd

matplotlib.use("AGG")

import warnings

import matplotlib as mpl

import matplotlib.pyplot as plt

from sklearn.impute import SimpleImputer

mpl.rcParams["axes.grid"] = False

plt.rcParams["font.family"] = "sans-serif"

warnings.filterwarnings("ignore")

\# 新增：获取环境信息

env_info = get_env_info(

["sklearn", "pandas", "matplotlib", "numpy"]

) # 检索对应import模块的版本信息

imputation_method = {

"mean": "均值",

"median": "中位数",

"most_frequent": "众数",

"nan": "空值",

"interpolate": "插值法",

"mul_interpolate": "多重插补",

}

dict_method = dict(imputation_method)

def data_filling(

df_input,

output_dir,

features,

method="median",

constant=None,

plot_feature=None,

dpi=600,

image_format="jpeg",

):

"""

一般数据填补

df_input:Dataframe 处理数据

features:list 填补特征(列)

method:str 填补方式(一般数据填补：'mean': '均值', 'median': '中位数', 'most_frequent': '众数',constant:‘常数填补',

'nan': '空值'，'interpolate': '插值法','mul_interpolate': '多重插补')

constant：object(int) 常数填补参数,表示将所有缺失填补成该值

plot_feature: str 用户选择需要生成分布曲线的单个特征，若不传则默认生成第一个填补特征

output_dir：图片保存路径

dpi：图片保存分辨率

image_format：图片另存格式

"""

try:

if features is None:

continuous_features, categorical_features, time_features = (

feature_classification(df_input)

)

features = continuous_features

df_temp = df_input[features]

else:

df_temp = df_input[features]

\# 对于均值、中位数、众数填补

if method in ["mean", "median", "most_frequent"]:

imp_ = SimpleImputer(missing_values=np.nan, strategy=method)

df_temp = pd.DataFrame(

data=imp_.fit_transform(df_temp), columns=features, index=df_temp.index

)

method_str = dict_method[method]

elif method == "constant":

imp_ = SimpleImputer(

missing_values=np.nan, strategy="constant", fill_value=constant

)

df_temp = pd.DataFrame(

data=imp_.fit_transform(df_temp), columns=features, index=df_temp.index

)

method_str = "常数" + str(constant)

elif method == "interpolate":

df_temp = df_temp.interpolate().bfill()

method_str = dict_method[method]

elif method == "nan":

\# 空值处理：不做处理，或者确保它是空值

method_str = dict_method.get(method, "空值")

elif method == "mul_interpolate":

from sklearn.experimental import enable_iterative_imputer

from sklearn.impute import IterativeImputer

imp_ = IterativeImputer(max_iter=10, random_state=0)

df_temp = pd.DataFrame(

data=imp_.fit_transform(df_temp), columns=features, index=df_temp.index

)

method_str = dict_method.get(method, "多重插补")

else:

raise ValueError("请输入正确的方法")

df_result = df_input.copy()

df_result[features] = df_temp[features]

\# 检查是否有意外的 NaN 产生

if (

df_result[features].isna().sum().sum() > 0

and not df_temp[features].isna().sum().sum() > 0

):

raise ValueError(

"数据填补合并时发生索引对齐错误，请检查输入数据的索引唯一性。"

)

if plot_feature is not None and isinstance(plot_feature, str):

features_to_plot = [plot_feature] if plot_feature in features else ([features[0]] if len(features) > 0 else [])

else:

features_to_plot = [features[0]] if len(features) > 0 else []

figures_list = distribution_curve(

df_input,

df_result,

features_to_plot,

output_dir,

method_name=method,

dpi=dpi,

image_format=image_format,

)

str_result = (

"采用" + method_str + "填补的方式对" + "、".join(features) + "进行数据填补"

)

input_o_desc, input_n_desc = describe(df_input[features])

result_o_desc, result_n_desc = describe(df_result[features])

input_o_desc.fillna("", inplace=True)

input_n_desc.fillna("", inplace=True)

result_o_desc.fillna("", inplace=True)

result_n_desc.fillna("", inplace=True)

tables_list = [

{

"name": "结果保存表",

"file": {"dataframe": df_result},

"description": "包含填补后特征的完整数据集。",

},

{

"name": "原分类变量描述表",

"file": {"dataframe": input_o_desc},

"description": "填补前分类变量的描述性统计。",

},

{

"name": "原连续变量描述表",

"file": {"dataframe": input_n_desc},

"description": "填补前连续变量的描述性统计。",

},

{

"name": "分析之后分类变量描述表",

"file": {"dataframe": result_o_desc},

"description": "填补后分类变量的描述性统计。",

},

{

"name": "分析之后连续变量描述表",

"file": {"dataframe": result_n_desc},

"description": "填补后连续变量的描述性统计。",

},

]

return {

"status": "success",

"description": str_result,

"tables": tables_list,

"figures": figures_list,

"environment": env_info,

}

except Exception:

raise

\# 2. 变量描述与单因素分析

import pandas as pd

import matplotlib

from core.utils.baseline_py.baseline_analysisutils import (

build_baseline_tables,

dataframe_to_categorical,

multi_groups_smart_analysis,

two_groups_smart_analysis,

)

from core.utils.feature_classification import feature_type_classification

from core.utils.get_environment import get_env_info

matplotlib.use('AGG')

import matplotlib.pyplot as plt

import matplotlib as mpl

mpl.rcParams['axes.grid'] = False

plt.rcParams['font.family'] = 'sans-serif'

plt.rcParams['font.sans-serif'] = ['Times New Roman + SimSun'] # 用来正常显示中文标签

plt.rcParams['axes.unicode_minus'] = False # 用来正常显示负号)

env_info = get_env_info(["pandas","matplotlib"]) # 检索对应import模块的版本信息

def comprehensive_smart_analysis(

df_input,

dependent_variable,

categorical_features=None,

continuous_features=None,

comparison=None,

show_method=True,

match=False,

pair_variable=None,

decimal_places=3,

table_mode="combined",

):

"""

综合智能统计分析

df_input:需处理的Dataframe

dependent_variable：分组变量str

categorical_features：分类变量名称list （可以放入定类或定量数据）

continuous_features：连续变量名称list （只能放定量数据）

comparison：分组标签list None,

show_method：bool 是否展示检验方法

match：是否为配对/匹配样本 bool False

pair_variable：配对或匹配样本ID列，match=True 时必须提供

decimal_places：保留小数位数

table_mode：结果表组织方式，'combined' 为合并表，'split' 为分类变量和连续变量分表

"""

try:

def miss_count(count,total):

try:

return total-int(count)

except:

return count

df_input=df_input.dropna(subset=[dependent_variable])

if categorical_features is not None:

df_input=dataframe_to_categorical(df_input, categorical_features)

if comparison is None:

comparison = df_input[dependent_variable].unique()

#数据类型不一致

try:

comparison.sort()

except Exception as e:

raise ValueError(f"因变量 '{dependent_variable}'中存在不一致的数据类型:{e}")

if (categorical_features is None) and (continuous_features is None):

df_temp = df_input.drop(columns=[dependent_variable])

continuous_features, categorical_features, time_features = feature_type_classification(df_temp)

if len(comparison)==2:

df_result, str_result=two_groups_smart_analysis(df_input=df_input, group=dependent_variable, categorical_features=categorical_features, continuous_features=continuous_features, group_labels=comparison, show_method=show_method, relate=match, decimal_num=decimal_places, pair_variable=pair_variable)

elif len(comparison)>2:

df_result, str_result=multi_groups_smart_analysis(df_input=df_input, group=dependent_variable, categorical_features=categorical_features, continuous_features=continuous_features, group_labels=comparison, show_method=show_method, relate=match, decimal_num=decimal_places, pair_variable=pair_variable)

else:

raise ValueError("分组变量中分类项少于2")

if df_result.empty:

return {

"status": "success",

"description": str_result, # 描述信息

"tables": [],

"figures": [],

"environment": env_info

}

str_result_="利用综合智能基线分析研究不同" + str(dependent_variable) + "分组中各个指标的差异是否具有统计学意义，总的有效样本为" + str(df_input.shape[0]) + '例，其中'

for la in comparison:

str_result_ += str(dependent_variable) + '=' + str(la) + '：病例数为' + str(list(df_input[dependent_variable]).count(la)) + '；'

str_result_+='\n（综合智能基线分析将根据样本的分布情况、方差齐性、样本量智能选择分析方法）：\n'

str_result=str_result_+str_result

df_result['总数']=df_result['总数'].apply(lambda x:miss_count(x,df_input.shape[0]))

df_result.rename(columns={'总览': '总览' + '\n' + ' (n=' + str(df_input.shape[0]) + ')','总数':'缺失'}, inplace=True)

tables_list = build_baseline_tables(

df_result=df_result,

categorical_features=categorical_features,

continuous_features=continuous_features,

table_mode=table_mode,

)

figures_list = [] # 做图片冗余

return {

"status": "success",

"description": str_result,

"tables": tables_list,

"figures": figures_list,

"environment": env_info

}

except Exception :

raise

\# 3. 分类多模型比较：LR、RF、SVM、AdaBoost、XGBoost，嵌套交叉验证

import datetime

import inspect

import math

import os

import random

import warnings

\# np.seterr(all='raise')

import matplotlib as mpl

import numpy as np

import pandas as pd

mpl.use("AGG")

from catboost import CatBoostClassifier

from ngboost import NGBClassifier

from core.utils.get_environment import get_env_info

from core.utils.ml.assistant import (bootstrap_test_metric_ci,

bootstrap_test_metric_sd, ci,

classification_metric_evaluate, dic2str,

get_numeric_auc, get_val,

make_class_metrics_dict,

normalize_comparison_plot_type,

plot_precision_recall_curve, round_dec,

save_fig)

from core.utils.ml.auc_delong_test import delong_roc_test

from core.utils.ml.GeneticSearch import GeneticSearchCV

from core.utils.ml.Hyperparameters import (BayesDefaultRange,

GeneticDefaultRange,

GridDefaultRange, HyperDefaultRange,

RandDefaultRange)

from core.utils.ml.HyperSearch import HyperoptSearchCV

from core.utils.ml.plotdraw import (

CB91_Grad_BP,

calculate_net_benefit_from_predictions,

forest_plot,

multimodel_auc_boxplot,

plot_decision_curves,

plot_multimodel_calibration_curves,

)

from core.utils.legend_utils import apply_roc_style_legend

current_time = datetime.datetime.now().strftime("%Y-%m-%d_%H-%M-%S")

from functools import reduce

import matplotlib.pyplot as plt

from lightgbm import LGBMClassifier

from sklearn.base import clone

from sklearn.ensemble import (AdaBoostClassifier, BaggingClassifier,

GradientBoostingClassifier,

RandomForestClassifier)

from sklearn.discriminant_analysis import LinearDiscriminantAnalysis

from sklearn.linear_model import LogisticRegression, SGDClassifier

from sklearn.metrics import auc

from sklearn.experimental import enable_halving_search_cv # noqa

from sklearn.model_selection import (

GridSearchCV,

HalvingGridSearchCV,

HalvingRandomSearchCV,

RandomizedSearchCV,

StratifiedKFold,

)

from sklearn.model_selection import train_test_split as TTS

from sklearn.naive_bayes import BernoulliNB, ComplementNB, GaussianNB

from sklearn.neighbors import KNeighborsClassifier

from sklearn.neural_network import MLPClassifier

from sklearn.preprocessing import StandardScaler, label_binarize

from sklearn.svm import SVC

from sklearn.tree import DecisionTreeClassifier

from skopt import BayesSearchCV

from xgboost import XGBClassifier

if not getattr(NGBClassifier.get_params, "_medstat_safe_patch", False):

def _ngboost_safe_get_params(self, deep=True):

"""

获取与 sklearn 克隆机制兼容的 NGBoost 构造参数

self:NGBClassifier NGBoost 分类器实例

deep:bool 是否递归获取参数

"""

params = {}

for name, parameter in inspect.signature(type(self).__init__).parameters.items():

if name == "self":

continue

if hasattr(self, name):

params[name] = getattr(self, name)

elif parameter.default is not inspect._empty:

params[name] = parameter.default

return params

_ngboost_safe_get_params._medstat_safe_patch = True

NGBClassifier.get_params = _ngboost_safe_get_params

if not getattr(NGBClassifier.fit, "_medstat_safe_patch", False):

_ngboost_original_fit = NGBClassifier.fit

def _ngboost_safe_fit(self, X, y, *args, **kwargs):

"""

拟合 NGBoost 并补充 sklearn 分类器所需的类别属性

self:NGBClassifier NGBoost 分类器实例

X:array-like 特征矩阵

y:array-like 二分类标签

"""

result = _ngboost_original_fit(self, X, y, *args, **kwargs)

self.classes_ = np.unique(np.asarray(y))

return result

_ngboost_safe_fit._medstat_safe_patch = True

NGBClassifier.fit = _ngboost_safe_fit

if hasattr(NGBClassifier, "__sklearn_tags__") and not getattr(

NGBClassifier.__sklearn_tags__, "_medstat_safe_patch", False

):

_ngboost_original_sklearn_tags = NGBClassifier.__sklearn_tags__

def _ngboost_safe_sklearn_tags(self):

"""

补充 NGBoost 的 sklearn 分类器标签

self:NGBClassifier NGBoost 分类器实例

"""

tags = _ngboost_original_sklearn_tags(self)

tags.estimator_type = "classifier"

return tags

_ngboost_safe_sklearn_tags._medstat_safe_patch = True

NGBClassifier.__sklearn_tags__ = _ngboost_safe_sklearn_tags

NGBClassifier._estimator_type = "classifier"

mpl.rcParams["axes.grid"] = False

plt.rcParams["font.sans-serif"] = ["Times New Roman + SimSun"] # 用来正常显示中文标签

plt.rcParams["axes.unicode_minus"] = False # 用来正常显示负号)

plt.rcParams["ps.useafm"] = True

plt.rcParams["font.family"] = "sans-serif"

plt.rcParams["pdf.fonttype"] = 42

warnings.filterwarnings("ignore")

random_model = [

"CatBoostClassifier",

"LGBMClassifier",

"NGBClassifier",

"XGBClassifier",

"XGBRegressor",

"RandomForestClassifier",

"AdaBoostClassifier",

"DecisionTreeClassifier",

"MLPClassifier",

"SVC",

"LogisticRegression",

"SGDClassifier",

"LogisticRegressionCV",

"RandomForestRegressor",

"GradientBoostingClassifier",

"AdaBoostRegressor",

"LinearSVR",

"LassoCV",

"PCA",

"GaussianMixture",

"KMeans",

"SpectralClustering",

]

\# 获取环境信息

env_info = get_env_info(

[

"pandas",

"numpy",

"sklearn",

"matplotlib",

"scipy",

"xgboost",

"lightgbm",

"catboost",

"ngboost",

]

)

def _set_custom_legend(ax_obj, loc_opt, **kwargs):

"""

根据 location 参数控制图例位置

:param ax_obj: matplotlib 的 ax 对象 或 plt

:param loc_opt: location 参数 (1, 2, 3, 4) location:图例位置,1-4对应不同位置，1：图像内部自适应放置 2：固定右下角 3：放置在图像外部, 4.放置图像下方

:param kwargs: 传递给 legend 的其他参数 (如 fontsize)

"""

return apply_roc_style_legend(ax_obj, legend_location=loc_opt, **kwargs)

def two_groups_classfication_multimodels(

df_input,

dependent_variable,

independent_variable,

output_dir,

methods=None,

decimal_places=3,

testsize=0.2,

boostrap=5,

randomState=42,

smooth=False,

searching="auto",

divide_testset=True,

label="LABEL",

testSet=False,

testLabel=0, # 测试集标签

pr_curve=False,

style="lancet",

dpi=600,

image_format="jpeg",

isKFold="nest",

resultType=0,

delong=False,

dca_cut=False,

n_splits=10,

scoring="roc_auc",

auto_model="RandomizedSearchCV",

location=1,

comparison_plot_type="boxplot",

**kwargs,

):

"""

@function：执行二分类任务的多模型比较分析。该函数集成了数据预处理、多模型训练、超参数自动搜索（网格/随机/贝叶斯/遗传算法等）、

交叉验证评估、以及全套可视化图表绘制（ROC、校准曲线、DCA、森林图、PR曲线）。

@input：

- df_input: 输入的数据集 (pandas DataFrame)

- dependent_variable: 因变量列名 (str)

- independent_variable: 自变量列名列表 (list of str)

- methods: 需要比较的模型名称列表 (list, 如 ['XGBClassifier', 'LogisticRegression'])

- decimal_places: 结果保留的小数位数 (int)

- testsize: 测试集划分比例 (float, 0.0-1.0)

- boostrap: 交叉验证折数或重采样次数 (int)

- randomState: 随机种子 (int)

- smooth: 是否对校准曲线进行平滑处理 (bool)

- searching: 调参模式 ('auto':自动寻参, 'default':默认参数, 'handle':手动参数)

- divide_testset: 是否独立划分测试集 (bool)

- label: 用于手动划分数据集的列名 (str, 仅当 testSet=True 时有效)

- testSet: 当 divide_testset=True 时，是否启用手动指定测试集模式 (bool)

- testLabel: 手动划分时，测试集在 label 列中的取值

- PR: 是否绘制精度-召回 (PR) 曲线 (bool)

- style: 绘图配色方案 ('lancet', 'nejm', 'jama', 'npg')

- dpi: 输出图片的分辨率 (int)

- image_format: 输出图片的格式 (str, 如 'jpeg', 'jpeg')

- isKFold: 验证策略 ('cross':普通交叉验证, 'nest':嵌套交叉验证, 其他:随机拆分重采样)

- output_dir: 结果保存文件夹路径 (str)

- resultType: 结果展示形式 (0: Mean ± SD, 1: Mean (95% CI))

- delong: 是否进行 Delong 检验以比较 AUC 差异 (bool)

- DCA_cut: DCA曲线是否截断负收益部分 (bool)

- n_splits: 自动寻参时内部交叉验证的折数 (int)

- scoring: 自动寻参时的优化指标 (str, 如 'roc_auc')

- auto_model: 自动寻参的具体算法 ('GridSearchCV', 'RandomizedSearchCV', 'BayesSearchCV', 'Hyperopt' 等)

- location:图例位置,1-4对应不同位置，1：图像内部自适应放置 2：固定右下角 3：放置在图像外部, 4.放置图像下方

- comparison_plot_type: 多模型验证集对比图形式 ('forest'/'boxplot')

- **kwargs: 手动模式下传入的模型参数字典

"""

\# 初始化返回结构列表

figures_list = []

tables_list = []

try:

test_ci_bootstrap = max(int(kwargs.get("test_ci_bootstrap", 1000)), 1)

comparison_plot_type = normalize_comparison_plot_type(comparison_plot_type)

palette_dict = {

"lancet": [

"#00468BFF",

"#ED0000FF",

"#42B540FF",

"#0099B4FF",

"#925E9FFF",

"#FDAF91FF",

"#AD002AFF",

"#ADB6B6FF",

"#1B1919FF",

],

"nejm": [

"#BC3C29FF",

"#0072B5FF",

"#E18727FF",

"#20854EFF",

"#7876B1FF",

"#6F99ADFF",

"#FFDC91FF",

"#EE4C97FF",

"#BC3C29FF",

],

"jama": [

"#374E55FF",

"#DF8F44FF",

"#00A1D5FF",

"#B24745FF",

"#79AF97FF",

"#6A6599FF",

"#80796BFF",

"#374E55FF",

"#DF8F44FF",

],

"npg": [

"#E64B35FF",

"#4DBBD5FF",

"#00A087FF",

"#3C5488FF",

"#F39B7FFF",

"#8491B4FF",

"#91D1C2FF",

"#DC0000FF",

"#7E6148FF",

"#B09C85FF",

],

}

str_time = (

str(datetime.datetime.now().hour)

+ str(datetime.datetime.now().minute)

+ str(datetime.datetime.now().second)

)

random_number = random.randint(1, 100)

str_time = str_time + str(random_number)

model_map = { # 模型映射字典

"LogisticRegression": LogisticRegression,

"CatBoostClassifier": CatBoostClassifier,

"NGBClassifier": NGBClassifier,

"XGBClassifier": XGBClassifier,

"RandomForestClassifier": RandomForestClassifier,

"LGBMClassifier": LGBMClassifier,

"SVC": SVC,

"MLPClassifier": MLPClassifier,

"GaussianNB": GaussianNB,

"ComplementNB": ComplementNB,

"AdaBoostClassifier": AdaBoostClassifier,

"LinearDiscriminantAnalysis": LinearDiscriminantAnalysis,

"BernoulliNB": BernoulliNB,

"SGDClassifier": SGDClassifier,

"KNeighborsClassifier": KNeighborsClassifier,

"DecisionTreeClassifier": DecisionTreeClassifier,

"BaggingClassifier": BaggingClassifier,

"GradientBoostingClassifier": GradientBoostingClassifier,

}

if methods is None:

methods = [

"LogisticRegression",

"RandomForestClassifier",

"SVC",

"AdaBoostClassifier",

"XGBClassifier",

]

if isinstance(divide_testset, str):

divide_testset = divide_testset not in [

"0",

"False",

"false",

"否",

"无",

"不划分",

]

else:

divide_testset = bool(divide_testset)

has_testset = divide_testset

features_flag = False

if divide_testset and testSet: # 固定测试集切割

if label in independent_variable or label == dependent_variable:

raise ValueError("标签列不能在所在模型中，请重新选择数据划分标签列！")

dftemp = df_input[

independent_variable + [dependent_variable] + [label]

].dropna()

features_flag = True

else:

dftemp = df_input[independent_variable + [dependent_variable]].dropna()

if len(dftemp) < 10:

raise ValueError(

f"有效样本量过少 ({len(dftemp)})，无法进行模型训练和交叉验证，请检查数据缺失情况。"

)

x = dftemp[independent_variable]

y = dftemp[dependent_variable]

\# 添加数据标准化

\# scaler = StandardScaler()

\# x = pd.DataFrame(scaler.fit_transform(x), columns=x.columns)

\# scaler = MinMaxScaler()

\# x = pd.DataFrame(scaler.fit_transform(x), columns=x.columns)

\# 样本数量检测

sample_count = len(dftemp)

if sample_count >= 20000 and auto_model == "BayesSearchCV":

invalid_models = []

if "SVC" in methods:

invalid_models.append("SVM")

if "GradientBoostingClassifier" in methods:

invalid_models.append("GBDT")

if invalid_models:

invalid_models_str = "、".join(invalid_models)

raise ValueError(

f"样本量过大 <{sample_count}> samples，{invalid_models_str} 模型暂不支持贝叶斯优化，"

f"请选择其他适合的模型或调参方法（如 RandomizedSearchCV）"

)

u = np.sort(np.unique(np.array(dftemp[dependent_variable])))

if len(u) == 2 and set(u) != {0, 1}:

y_result = label_binarize(

dftemp[dependent_variable], classes=[ii for ii in u]

) # 将标签二值化

y_result_pd = pd.DataFrame(y_result, columns=[dependent_variable])

df = pd.concat(

[dftemp.drop(dependent_variable, axis=1), y_result_pd], axis=1

)

x = df[independent_variable]

y = df[dependent_variable]

elif len(u) > 2:

raise ValueError("暂时只支持二分类。请检查因变量取值情况。")

\# 构造绝对独立的测试集.

\# X_pool: 用于 CV 训练和验证

\# X_test_holdout: 独立测试集，不参与任何训练和调参

if divide_testset and testSet:

if isinstance(dftemp[label].iloc[0], str):

testLabel = str(testLabel)

test_mask = dftemp[label] == testLabel

X_pool = x.loc[~test_mask]

X_test_holdout = x.loc[test_mask]

y_pool = y.loc[~test_mask]

y_test_holdout = y.loc[test_mask]

if X_pool.shape[0] == 0 or X_test_holdout.shape[0] == 0:

raise ValueError("固定测试集划分失败，请检查 testLabel 是否存在于标签列中。")

elif divide_testset:

X_pool, X_test_holdout, y_pool, y_test_holdout = TTS(

x, y, test_size=testsize, random_state=randomState, stratify=y

)

else:

X_pool, y_pool = x.copy(), y.copy()

X_test_holdout, y_test_holdout = None, None

name_dict = {

"LogisticRegression": "logistic",

"CatBoostClassifier": "CatBoost",

"NGBClassifier": "NGBoost",

"XGBClassifier": "XGBoost",

"RandomForestClassifier": "RandomForest",

"LGBMClassifier": "LightGBM",

"SVC": "SVM",

"MLPClassifier": "MLP",

"GaussianNB": "GNB",

"ComplementNB": "CNB",

"AdaBoostClassifier": "AdaBoost",

"LinearDiscriminantAnalysis": "LDA",

"BernoulliNB": "BernoulliNB",

"SGDClassifier": "SGD",

"KNeighborsClassifier": "KNN",

"DecisionTreeClassifier": "DecisionTree",

"BaggingClassifier": "Bagging",

"GradientBoostingClassifier": "GBDT",

}

if len(methods) == 0:

methods = [

"LogisticRegression",

"XGBClassifier",

"RandomForestClassifier",

]

for m in methods:

if m not in model_map:

raise ValueError(

f"不支持的模型方法: {m}。当前支持的有: {list(model_map.keys())}"

)

str_result = "已完成算法：分类模型比较\n本算法采用多种机器学习模型尝试完成数据样本分类任务，包括：{}。各模型的参数值选取情况如下所示：\n\n".format(

methods

)

plot_name_list = []

plot_name_dict = {}

plot_name_dict_save = {}

fig, ax = plt.subplots(figsize=(4, 4), dpi=dpi)

\# 画对角线

ax.plot(

[0, 1],

[0, 1],

linestyle="--",

lw=1,

color="grey",

alpha=1.0,

)

ax.set_xlim([-0.02, 1.02])

ax.set_ylim([-0.02, 1.02])

ax.tick_params(top=False, right=False)

\# ax.legend(bbox_to_anchor=(1.05, 1), loc=2, borderaxespad=0)

ax.set_xlabel("1-Specificity")

ax.set_ylabel("Sensitivity")

ax.set_title("ROC curve(Validation)")

mean_fpr = np.linspace(0, 1, 100)

colors = CB91_Grad_BP

metric_cols = list(make_class_metrics_dict().keys())

df_0_train = pd.DataFrame(columns=metric_cols, index=[0])

df_0_val = pd.DataFrame(columns=metric_cols, index=[0])

df_0_test = pd.DataFrame(columns=metric_cols, index=[0])

df_plot = pd.DataFrame(columns=["method", "mean", "std"])

val_auc_boxplot_records = []

fpr_train_alls, tpr_train_alls, train_method_alls, mean_auc_train_alls = (

[],

[],

[],

[],

)

AUC_95CI_test, AUC_95CI_SD_test, AUC_95CI_train, AUC_95CI_SD_train = (

[],

[],

[],

[],

)

validation_calibration_dict = {}

test_calibration_dict = {}

validation_dca_dict = {}

test_dca_dict = {}

model_test_data_all = {}

sdorci = " SD " if resultType == 0 else " 95%CI "

X_train_ps, Y_train_ps, model_train_s = [], [], [] # PR曲线

X_test_ps, Y_test_ps = [], []

holdout_tpr_dict = {} # 用于存储每个模型的测试集平均TPR曲线

name = []

plot_name_dict1 = {} # 用于存储寻参相关的图片文件名

hyperopt_losses_dict = {}

\# 在进入模型循环前，先对总池子数据进行标准化，用于寻参

\# 注意：后续的交叉验证内部还会进行局部标准化以严谨评估

pool_scaler = StandardScaler()

X_pool_scaled = pd.DataFrame(

pool_scaler.fit_transform(X_pool), columns=X_pool.columns

)

if has_testset:

X_test_holdout_scaled = pd.DataFrame(

pool_scaler.transform(X_test_holdout), columns=X_test_holdout.columns

)

else:

X_test_holdout_scaled = None

for i, method in enumerate(methods):

tprs_train, tprs_test, tprs_holdout = [], [], [] # 添加 tprs_holdout

list_evaluate_dic_train = make_class_metrics_dict()

list_evaluate_dic_val = make_class_metrics_dict()

list_evaluate_dic_test = make_class_metrics_dict()

conf_dic_train, conf_dic_val, conf_dic_test = {}, {}, {}

test_bootstrap_conf_dic = {}

test_bootstrap_sd_dic = {}

name.append(name_dict[method])

model_class = model_map[method]

\# 为新增模型设置可复现且兼容概率预测的基础参数

base_model_kwargs = {}

if method == "CatBoostClassifier":

base_model_kwargs.update(

{

"verbose": 0,

"allow_writing_files": False,

"loss_function": "Logloss",

"random_state": randomState,

}

)

elif method == "NGBClassifier":

base_model_kwargs.update(

{

"verbose": False,

"verbose_eval": 0,

"random_state": randomState,

}

)

elif method == "SGDClassifier":

base_model_kwargs.update(

{

"loss": "log_loss",

"early_stopping": True,

"random_state": randomState,

}

)

elif method == "BernoulliNB":

base_model_kwargs.update({"binarize": 0.0})

if searching == "auto":

\# 网格寻参与随机寻参方法重写·

if (

method in ["MLPClassifier", "ComplementNB", "GaussianNB"]

and auto_model == "Hyperopt"

):

raise ValueError(

f"不允许使用 {method} 模型搭配 Hyperopt 寻参方法，请选择其他寻参方法。"

)

if auto_model == "RandomizedSearchCV": # 随机寻参

searcher = RandomizedSearchCV(

model_class(**base_model_kwargs),

param_distributions=RandDefaultRange[method],

random_state=randomState,

n_jobs=-1,

)

elif auto_model == "GridSearchCV": # 网格寻参

searcher = GridSearchCV(

model_class(**base_model_kwargs),

param_grid=GridDefaultRange[method],

n_jobs=-1,

)

elif auto_model == "BayesSearchCV": # 贝叶斯寻参

searcher = BayesSearchCV(

model_class(**base_model_kwargs),

search_spaces=BayesDefaultRange[method],

n_jobs=-1,

random_state=randomState,

n_iter=10,

)

elif auto_model == "HalvingGridSearchCV": # 连续二分网格搜索

searcher = HalvingGridSearchCV(

model_class(**base_model_kwargs),

param_grid=GridDefaultRange[method],

n_jobs=-1,

random_state=randomState,

min_resources="exhaust" if method == "NGBClassifier" else "smallest",

)

elif auto_model == "HalvingRandomSearchCV": # 连续二分随机搜索

searcher = HalvingRandomSearchCV(

model_class(**base_model_kwargs),

param_distributions=RandDefaultRange[method],

n_jobs=-1,

random_state=randomState,

min_resources="exhaust" if method == "NGBClassifier" else "smallest",

)

elif auto_model == "GeneticSearchCV": # 遗传算法寻参

searcher = GeneticSearchCV(

model_class(**base_model_kwargs),

param_grid=GeneticDefaultRange[method],

n_generations=10,

population_size=50,

mutation_rate=0.1,

crossover_rate=0.8,

elite_size=2,

scoring=scoring,

cv=n_splits,

random_state=randomState,

n_jobs=-1,

)

elif auto_model == "Hyperopt": # Hyperopt 算法寻参

searcher = HyperoptSearchCV(

model_class(**base_model_kwargs),

param_space=HyperDefaultRange[method],

max_evals=kwargs.get("max_evals", 10),

scoring=scoring,

cv=n_splits,

random_state=randomState,

n_jobs=-1,

)

searcher.fit(X_pool_scaled, y_pool)

selected_model = searcher.best_estimator_

if auto_model == "Hyperopt":

trials_manager = getattr(searcher, "trials_", None)

if trials_manager is not None and hasattr(trials_manager, "trials"):

\# 获取所有迭代的损失值

losses = [t["result"]["loss"] for t in trials_manager.trials]

if losses:

hyperopt_losses_dict[name_dict[method]] = losses

elif searching == "default":

if method == "SVC":

selected_model = model_class(probability=True, random_state=42)

elif method == "MLPClassifier":

selected_model = model_class(

hidden_layer_sizes=(20, 10), max_iter=20, random_state=42

)

elif method == "RandomForestClassifier":

selected_model = model_class(n_estimators=20, random_state=42)

else:

default_model_kwargs = base_model_kwargs.copy()

if method in ["CatBoostClassifier", "NGBClassifier"]:

default_model_kwargs["n_estimators"] = 50

if method in random_model:

default_model_kwargs["random_state"] = randomState

selected_model = model_class(**default_model_kwargs)

elif searching == "handle":

method_dicts = kwargs

if i == 0:

me_count = True

for me_list in methods:

if me_list in method_dicts.keys():

me_count = False

continue

if me_count:

raise ValueError("请设置要调参的模型！")

if method in method_dicts.keys():

method_dict = base_model_kwargs.copy()

if method == "SVC":

method_dict.update({"probability": True})

method_dict.update(method_dicts[method])

if (

method == "RandomForestClassifier"

and method_dict["max_depth"] == "None"

):

method_dict["max_depth"] = None

if (

method == "DecisionTreeClassifier"

and method_dict["max_depth"] == "None"

):

method_dict["max_depth"] = None

if method == "MLPClassifier":

hls_vals = str(method_dict["hidden_layer_sizes"]).split(",")

hls_value = ()

for hls_val in hls_vals:

try:

if int(hls_val) >= 5 and int(hls_val) <= 200:

hls_value = hls_value + (int(hls_val),)

else:

raise ValueError("请按照要求重新设置宽度！")

except:

raise ValueError("请重新设神经网络模型中的隐藏层宽度！")

method_dict["hidden_layer_sizes"] = hls_value

if method == "GaussianNB" and method_dict["priors"] == "None":

method_dict["priors"] = None

elif method == "GaussianNB":

pri_vals = str(method_dict["priors"]).split(",")

pri_value = ()

pri_sum = 0.0

for pri_val in pri_vals:

try:

pri_sum = float(pri_val) + pri_sum

pri_value = pri_value + (float(pri_val),)

except:

raise ValueError("请重新设朴素贝叶斯模型中的先验概率！")

if len(pri_vals) == len(np.unique(y)) and pri_sum == 1.0:

method_dict["priors"] = pri_value

else:

raise ValueError("请重新设朴素贝叶斯模型中的先验概率！")

if method in random_model:

method_dict["random_state"] = 42

selected_model = model_class(**method_dict)

else:

searcher = RandomizedSearchCV(

model_class(**base_model_kwargs),

param_distributions=GridDefaultRange[method],

)

selected_model = searcher.fit(x, y)

selected_params = base_model_kwargs.copy()

selected_params.update(selected_model.best_params_)

selected_model = model_class(**selected_params)

list_evaluate_dic_train = make_class_metrics_dict()

list_evaluate_dic_test = make_class_metrics_dict()

data_all = {}

test_data_delong = {}

conf_dic_train, conf_dic_test = {}, {}

if isKFold == "cross":

KF = StratifiedKFold(

n_splits=boostrap, random_state=randomState, shuffle=True

)

for i_k, (train_index, valid_index) in enumerate(

KF.split(X_pool, y_pool)

):

\# Xval 是交叉验证内部的验证集,提取原始数据

Xtrain_raw, Xval_raw = (

X_pool.iloc[train_index],

X_pool.iloc[valid_index],

)

Ytrain, Yval = y_pool.iloc[train_index], y_pool.iloc[valid_index]

if has_testset:

Xtest_raw = X_test_holdout

Ytest = y_test_holdout

else:

Xtest_raw = Xval_raw

Ytest = Yval

\# 仅在训练集上 fit

scaler = StandardScaler()

Xtrain = pd.DataFrame(

scaler.fit_transform(Xtrain_raw), columns=Xtrain_raw.columns

)

Xval = pd.DataFrame(

scaler.transform(Xval_raw), columns=Xval_raw.columns

)

Xtest = pd.DataFrame(

scaler.transform(Xtest_raw), columns=Xtest_raw.columns

)

data_all.update(

{

i_k: {

"Xtrain": Xtrain,

"Ytrain": Ytrain,

"Xval": Xval,

"Yval": Yval, # 验证集

"Xtest": Xtest,

"Ytest": Ytest, # 测试集

}

}

)

test_data_delong.update({i_k: np.array(Ytest)})

elif isKFold == "nest":

KF = StratifiedKFold(

n_splits=boostrap, random_state=randomState, shuffle=True

)

inner_cv = StratifiedKFold(

n_splits=boostrap, random_state=randomState, shuffle=True

)

for i_k, (train_index, valid_index) in enumerate(

KF.split(X_pool, y_pool)

):

\# 嵌套切分的外层：先在训练池中划分训练集和验证集

Xtrain_raw, Xval_raw = (

X_pool.iloc[train_index],

X_pool.iloc[valid_index],

)

Ytrain, Yval = y_pool.iloc[train_index], y_pool.iloc[valid_index]

\# 当指定固定测试集时，测试集始终保持独立；否则沿用外层验证集

if has_testset:

Xtest_raw = X_test_holdout

Ytest = y_test_holdout

else:

Xtest_raw = Xval_raw

Ytest = Yval

\# 局部标准化

scaler = StandardScaler()

Xtrain = pd.DataFrame(

scaler.fit_transform(Xtrain_raw), columns=Xtrain_raw.columns

)

Xval = pd.DataFrame(

scaler.transform(Xval_raw), columns=Xval_raw.columns

)

Xtest = pd.DataFrame(

scaler.transform(Xtest_raw), columns=Xtest_raw.columns

)

data_all.update(

{

i_k: {

"Xtrain": Xtrain,

"Ytrain": Ytrain,

"Xval": Xval,

"Yval": Yval,

"Xtest": Xtest,

"Ytest": Ytest,

}

}

)

test_data_delong.update({i_k: np.array(Ytest)})

else:

for index in range(0, boostrap):

if boostrap == 1:

if has_testset and testSet: # 测试集数据划分

if isinstance(dftemp[label][0], str):

testLabel = str(testLabel)

train_a = dftemp[dftemp[label] != testLabel]

test_a = dftemp[dftemp[label] == testLabel]

Xtrain_raw = train_a[independent_variable]

Ytrain = train_a[dependent_variable]

Xtest_raw = test_a[independent_variable]

Ytest = test_a[dependent_variable]

else:

X = dftemp[independent_variable]

Y = dftemp[dependent_variable]

Xtrain_raw, Xtest_raw, Ytrain, Ytest = TTS(

X,

Y,

test_size=testsize,

random_state=randomState,

stratify=Y,

)

else:

split_random_state = randomState + index

Xtrain, Xtest, Ytrain, Ytest = TTS(

x,

y,

test_size=testsize,

random_state=split_random_state,

stratify=y,

)

data_all.update(

{

index: {

"Xtrain": Xtrain,

"Ytrain": Ytrain,

"Xval": Xtest, # 非Cross模式下，测试集即为验证集

"Yval": Ytest,

"Xtest": Xtest,

"Ytest": Ytest,

}

}

)

test_data_delong.update({index: np.array(Ytest)})

if method == methods[0]:

model_test_data_all.update({"original": test_data_delong})

test_all_data_delong = {}

X_train_p, Y_train_p, model_train = [], [], [] ##PR曲线

X_test_p, Y_test_p = [], []

validation_y_parts = []

validation_probability_parts = []

\# 训练最终模型 (使用之前已标准化好的 X_pool_scaled)

if has_testset:

final_model_for_test = clone(selected_model).fit(X_pool_scaled, y_pool)

\# 计算独立测试集的指标（仅执行一次）

fpr_test_final, tpr_test_final, metric_dic_test_final, _ = (

classification_metric_evaluate(

final_model_for_test,

X_test_holdout_scaled,

y_test_holdout,

Threshold=None,

)

)

\# 记录概率用于 Delong 或其他用途

Yprob_test_final = final_model_for_test.predict_proba(

X_test_holdout_scaled

)[:, 1]

model_display_name = name_dict[method]

test_calibration_dict[model_display_name] = {

"y_true": np.asarray(y_test_holdout),

"y_prob": Yprob_test_final,

}

test_p_serie, test_net_benefit, test_treat_all = (

calculate_net_benefit_from_predictions(

y_test_holdout, Yprob_test_final

)

)

test_dca_dict[model_display_name] = {

"p_serie": test_p_serie,

"net_b_s": test_net_benefit,

"net_b_s_A": test_treat_all,

}

if resultType == 1:

test_bootstrap_conf_dic = bootstrap_test_metric_ci(

y_test_holdout,

Yprob_test_final,

n_bootstrap=test_ci_bootstrap,

random_state=randomState,

)

elif resultType == 0:

test_bootstrap_sd_dic = bootstrap_test_metric_sd(

y_test_holdout,

Yprob_test_final,

n_bootstrap=test_ci_bootstrap,

random_state=randomState,

)

else:

metric_dic_test_final = None

for data_key, data_value in data_all.items():

Xtrain, Ytrain = data_value["Xtrain"], data_value["Ytrain"]

Xtest, Ytest = data_value["Xtest"], data_value["Ytest"]

\# cross 模式，字典里有 Xval bootstrap/nest，通常只有 Xtest。

if "Xval" in data_value:

Xval, Yval = data_value["Xval"], data_value["Yval"]

else:

\# 在 Bootstrap 或 Nest 模式下，当前折的测试集充当验证

Xval, Yval = data_value["Xtest"], data_value["Ytest"]

if isKFold == "nest":

best_auc = 0.0

resThreshold = 0

for j, (train_index_inner, test_index_inner) in enumerate(

inner_cv.split(Xtrain, Ytrain)

):

X_train_inner, X_test_inner = (

Xtrain.iloc[train_index_inner],

Xtrain.iloc[test_index_inner],

)

y_train_inner, y_test_inner = (

Ytrain.iloc[train_index_inner],

Ytrain.iloc[test_index_inner],

)

model_i = clone(selected_model).fit(

X_train_inner, y_train_inner

)

_, _, metric_dic_train_i, _ = classification_metric_evaluate(

model_i, X_train_inner, y_train_inner, True

)

_, _, metric_dic_valid_i, _ = classification_metric_evaluate(

model_i,

X_test_inner,

y_test_inner,

True,

Threshold=metric_dic_train_i["cutoff"],

)

if metric_dic_valid_i["AUC"] > best_auc:

model = model_i

resThreshold = metric_dic_train_i["cutoff"]

else:

model = clone(selected_model).fit(Xtrain, Ytrain)

resThreshold = None

\# 记录模型和数据（用于后续PR曲线绘制）

X_train_p.append(Xtrain)

Y_train_p.append(Ytrain)

model_train.append(model)

X_test_p.append(Xtest)

Y_test_p.append(Ytest)

\# 计算概率

Yprob_test = model.predict_proba(Xtest)[:, 1]

test_all_data_delong.update({data_key: Yprob_test})

\# 汇总每一折的验证集预测，构建完整 OOF 校准与 DCA 数据

prob_pos_val = model.predict_proba(Xval)[:, 1]

validation_y_parts.append(np.asarray(Yval).reshape(-1))

validation_probability_parts.append(

np.asarray(prob_pos_val, dtype=float).reshape(-1)

)

\# #指标评估

\# #训练集指标

\# fpr_train, tpr_train, metric_dic_train, _ = classification_metric_evaluate(

\#	model, Xtrain, Ytrain, Threshold=resThreshold

\# )

\#

\# #验证集指标 (训练集cutoff)

\# fpr_val, tpr_val, metric_dic_val, _ = classification_metric_evaluate(

\#	model, Xval, Yval, Threshold=metric_dic_train["cutoff"]

\# )

\#

\# #测试集指标 (训练集cutoff)

\# fpr_test, tpr_test, metric_dic_test, _ = classification_metric_evaluate(

\#	model, Xtest, Ytest, Threshold=metric_dic_train["cutoff"]

\# )

\# 计算当前折的训练和验证指标 (用于填充表格中的 Train 和 Val 列)

fpr_train, tpr_train, metric_dic_train, _ = (

classification_metric_evaluate(

model, Xtrain, Ytrain, Threshold=resThreshold

)

)

fpr_val, tpr_val, metric_dic_val, _ = classification_metric_evaluate(

model, Xval, Yval, Threshold=metric_dic_train["cutoff"]

)

\# 将外层计算的固定测试指标放入字典，确保无论多少折，其值永远一致

if has_testset:

metric_dic_test = metric_dic_test_final.copy()

else:

metric_dic_test = metric_dic_val.copy()

\# 记录验证集的曲线

tprs_train.append(np.interp(mean_fpr, fpr_train, tpr_train))

tprs_test.append(np.interp(mean_fpr, fpr_val, tpr_val))

tprs_train[-1][0] = 0.0

tprs_test[-1][0] = 0.0

\# 将结果存入三份不同的汇总列表中

for key in list_evaluate_dic_train.keys():

list_evaluate_dic_train[key].append(metric_dic_train[key])

list_evaluate_dic_val[key].append(metric_dic_val[key]) # 存验证集

list_evaluate_dic_test[key].append(

metric_dic_test[key]

) # 存独立测试集

model_test_data_all.update({method: test_all_data_delong})

\# 使用全部验证折的 OOF 预测生成统一的验证集曲线数据

validation_y = np.concatenate(validation_y_parts)

validation_probability = np.concatenate(validation_probability_parts)

model_display_name = name_dict[method]

validation_calibration_dict[model_display_name] = {

"y_true": validation_y,

"y_prob": validation_probability,

}

validation_p_serie, validation_net_benefit, validation_treat_all = (

calculate_net_benefit_from_predictions(

validation_y, validation_probability

)

)

validation_dca_dict[model_display_name] = {

"p_serie": validation_p_serie,

"net_b_s": validation_net_benefit,

"net_b_s_A": validation_treat_all,

}

X_train_ps.append(X_train_p)

Y_train_ps.append(Y_train_p)

model_train_s.append(model_train)

X_test_ps.append(X_test_p)

Y_test_ps.append(Y_test_p)

val_auc_boxplot_records.extend(

[

{"method": name_dict[method], "AUC": float(val_auc)}

for val_auc in list_evaluate_dic_val["AUC"]

if pd.notna(val_auc)

]

)

for key in list_evaluate_dic_train.keys():

if len(list_evaluate_dic_train[key]) > 0:

metric_dic_train[key] = np.nanmean(

list_evaluate_dic_train[key]

) # 使用nanmean忽略NaN

else:

metric_dic_train[key] = np.nan # 或者 0

\# 验证集

if len(list_evaluate_dic_val[key]) > 0:

metric_dic_val[key] = np.nanmean(list_evaluate_dic_val[key])

else:

metric_dic_val[key] = np.nan

\# 测试集

if len(list_evaluate_dic_test[key]) > 0:

metric_dic_test[key] = np.nanmean(list_evaluate_dic_test[key])

else:

metric_dic_test[key] = np.nan

if resultType == 0: ##SD

list_evaluate_dic_train[key] = (

np.std(list_evaluate_dic_train[key], axis=0)

if len(list_evaluate_dic_train[key]) > 0

else 0

)

list_evaluate_dic_val[key] = (

np.std(list_evaluate_dic_val[key], axis=0)

if len(list_evaluate_dic_val[key]) > 0

else 0

)

if has_testset and key in test_bootstrap_sd_dic:

list_evaluate_dic_test[key] = test_bootstrap_sd_dic[key]

else:

list_evaluate_dic_test[key] = (

np.std(list_evaluate_dic_test[key], axis=0)

if len(list_evaluate_dic_test[key]) > 0

else 0

)

elif resultType == 1: ##CI

conf_dic_train[key] = (

list(ci(list_evaluate_dic_train[key]))

if len(list_evaluate_dic_train[key]) > 0

else [0, 0]

)

conf_dic_val[key] = (

list(ci(list_evaluate_dic_val[key]))

if len(list_evaluate_dic_val[key]) > 0

else [0, 0]

)

if has_testset and key != "AUC" and key in test_bootstrap_conf_dic:

conf_dic_test[key] = test_bootstrap_conf_dic[key]

else:

conf_dic_test[key] = (

list(ci(list_evaluate_dic_test[key]))

if len(list_evaluate_dic_test[key]) > 0

else [0, 0]

)

result_dic_train = metric_dic_train

result_dic_val = metric_dic_val

result_dic_test = metric_dic_test

if resultType == 0: ##SD

for tem in ["AUC_L", "AUC_U"]:

del list_evaluate_dic_train[tem]

del list_evaluate_dic_val[tem]

del list_evaluate_dic_test[tem]

for key in list_evaluate_dic_train.keys():

\# 格式化训练集

result_dic_train[key] = (

f"{round_dec(float(metric_dic_train[key]), d=decimal_places)}({round_dec(float(list_evaluate_dic_train[key]), d=decimal_places)})"

)

\# 格式化验证集

result_dic_val[key] = (

f"{round_dec(float(metric_dic_val[key]), d=decimal_places)}({round_dec(float(list_evaluate_dic_val[key]), d=decimal_places)})"

)

\# 格式化测试集

result_dic_test[key] = (

f"{round_dec(float(metric_dic_test[key]), d=decimal_places)}({round_dec(float(list_evaluate_dic_test[key]), d=decimal_places)})"

)

if key == "AUC":

result_dic_train["AUC(95%CI)"] = result_dic_train[key]

result_dic_val["AUC(95%CI)"] = result_dic_val[key] # 新增

result_dic_test["AUC(95%CI)"] = result_dic_test[key]

elif resultType == 1:

for tem in ["AUC_L", "AUC_U"]:

del conf_dic_train[tem]

del conf_dic_val[tem]

del conf_dic_test[tem]

for key in conf_dic_train.keys():

if key == "AUC":

result_dic_train["AUC(95%CI)"] = (

str(round_dec(float(metric_dic_train[key]), decimal_places))

+ " ("

+ str(

round_dec(

float(metric_dic_train["AUC_L"]), decimal_places

)

)

+ "-"

+ str(

round_dec(

float(metric_dic_train["AUC_U"]), decimal_places

)

)

+ ")"

)

result_dic_test["AUC(95%CI)"] = (

str(round_dec(float(metric_dic_test[key]), decimal_places))

+ " ("

+ str(

round_dec(

float(metric_dic_test["AUC_L"]), decimal_places

)

)

+ "-"

+ str(

round_dec(

float(metric_dic_test["AUC_U"]), decimal_places

)

)

+ ")"

)

result_dic_val["AUC(95%CI)"] = (

str(round_dec(float(metric_dic_val[key]), decimal_places))

+ " ("

+ str(

round_dec(

float(metric_dic_val["AUC_L"]), decimal_places

)

)

+ "-"

+ str(

round_dec(

float(metric_dic_val["AUC_U"]), decimal_places

)

)

+ ")"

)

else:

result_dic_train[key] = (

str(round(float(metric_dic_train[key]), decimal_places))

+ "("

+ str(

round_dec(

float(conf_dic_train[key][0]), d=decimal_places

)

)

+ "-"

+ str(

round_dec(

float(conf_dic_train[key][1]), d=decimal_places

)

)

+ ")"

)

result_dic_test[key] = (

str(round(float(metric_dic_test[key]), decimal_places))

+ "("

+ str(

round_dec(

float(conf_dic_test[key][0]), d=decimal_places

)

)

+ "-"

+ str(

round_dec(

float(conf_dic_test[key][1]), d=decimal_places

)

)

+ ")"

)

result_dic_val[key] = (

str(round(float(metric_dic_val[key]), decimal_places))

+ "("

+ str(

round_dec(float(conf_dic_val[key][0]), d=decimal_places)

)

+ "-"

+ str(

round_dec(float(conf_dic_val[key][1]), d=decimal_places)

)

+ ")"

)

df_train_result = pd.DataFrame([result_dic_train], index=["Mean"])

df_val_result = pd.DataFrame([result_dic_val], index=["Mean"])

df_test_result = pd.DataFrame([result_dic_test], index=["Mean"])

df_train_result["Classifications"] = name_dict[method]

df_val_result["Classifications"] = name_dict[method]

df_test_result["Classifications"] = name_dict[method]

AUC_95CI_test.append(list(df_test_result.iloc[0, -4:-2]))

AUC_95CI_train.append(list(df_train_result.iloc[0, -4:-2]))

df_0_train = pd.concat([df_0_train, df_train_result])

df_0_val = pd.concat([df_0_val, df_val_result]) # 对应 CV 验证

df_0_test = pd.concat([df_0_test, df_test_result]) # 对应独立测试

if len(tprs_train) > 0:

mean_tpr_train = np.mean(tprs_train, axis=0)

mean_tpr_train[-1] = 1.0

else:

mean_tpr_train = np.linspace(0, 1, 100) # 默认对角线

if len(tprs_test) > 0:

mean_tpr_test = np.mean(tprs_test, axis=0)

mean_tpr_test[-1] = 1.0

else:

mean_tpr_test = np.linspace(0, 1, 100)

mean_auc_train = auc(mean_fpr, mean_tpr_train) # 计算训练集平均AUC值

mean_auc_test = auc(mean_fpr, mean_tpr_test)

###画训练集ROC

fpr_train_alls.append(mean_fpr)

tpr_train_alls.append(mean_tpr_train)

train_method_alls.append(method)

mean_auc_train_alls.append(mean_auc_train)

std_value = 0

if resultType == 0:

std_value = list_evaluate_dic_val["AUC"] # 取多折或者抽样的验证变动范围标准偏差(真实变幅值非测试常零的SD)

elif resultType == 1:

std_value = (conf_dic_val["AUC"][1] - conf_dic_val["AUC"][0]) / 2 # 用 Val 的波动获取误差

new_row = pd.DataFrame(

[

{

"method": name_dict[method],

"mean": mean_auc_test,

"std": std_value,

}

]

)

df_plot = pd.concat([df_plot, new_row], ignore_index=True)

if has_testset:

\# 测试集auc数据

mean_tpr_holdout = np.interp(mean_fpr, fpr_test_final, tpr_test_final)

mean_tpr_holdout[0] = 0.0

mean_tpr_holdout[-1] = 1.0

holdout_tpr_dict[method] = mean_tpr_holdout

mean_auc_holdout_val = auc(mean_fpr, mean_tpr_holdout)

\# 这里的 std 使用测试集的标准差

std_test = (

test_bootstrap_sd_dic.get("AUC", 0)

if resultType == 0

else 0

)

if "df_plot_test" not in locals(): # 初始化

df_plot_test = pd.DataFrame(columns=["method", "mean", "std"])

new_row_test = pd.DataFrame(

[

{

"method": name_dict[method],

"mean": mean_auc_holdout_val,

"std": std_test,

}

]

)

df_plot_test = pd.concat([df_plot_test, new_row_test], ignore_index=True)

ax.plot(

mean_fpr,

mean_tpr_test,

c=palette_dict[style][i],

label=name_dict[method]

+ "(AUC = "

+ df_val_result["AUC(95%CI)"][0][

: df_val_result["AUC(95%CI)"][0].find("(")

].strip()

+ sdorci

+ df_val_result["AUC(95%CI)"][0][

df_val_result["AUC(95%CI)"][0].find("(") :

].strip()

+ ")",

lw=1.5,

alpha=1,

)

if searching == "auto":

if auto_model == "Hyperopt": # Hyperopt的情况

str_result += (

method

+ ": AUC="

+ str(round_dec(mean_auc_train, decimal_places))

+ "; 模型参数:\n"

+ dic2str(selected_model.get_params(), method)

+ "\n"

)

else: # 其他自动寻参方法

selected_model = searcher.fit(x, y)

str_result += (

method

+ ": AUC="

+ str(round_dec(mean_auc_train, decimal_places))

+ "; 模型参数:\n"

+ dic2str(searcher.best_params_, method)

+ "\n"

)

else:

str_result += (

method

+ ": AUC="

+ str(round_dec(mean_auc_train, decimal_places))

+ "; 模型参数:\n"

+ dic2str(selected_model.get_params(), method)

+ "\n"

)

###模型德龙检测

if delong:

delong_z, delong_p = [], []

for i in range(boostrap):

zzz, ppp = [], []

for method1 in methods:

zz, pp = [], []

for method2 in methods:

z, p = delong_roc_test(

model_test_data_all["original"][i],

model_test_data_all[method1][i],

model_test_data_all[method2][i],

)

\# 检查是否为浮点数

if isinstance(z, float):

zz.append(z)

pp.append(p)

else:

zz.append(z[0][0])

pp.append(p[0][0])

zzz.append(zz)

ppp.append(pp)

delong_z.append(zzz)

delong_p.append(ppp)

if boostrap == 1:

delong_zz1 = pd.DataFrame(

reduce(lambda x, y: np.array(x) + np.array(y), delong_z),

index=methods,

columns=methods,

)

delong_pp1 = pd.DataFrame(

reduce(lambda x, y: np.array(x) + np.array(y), delong_p),

index=methods,

columns=methods,

)

else:

if len(delong_z) > 0:

delong_zz1 = pd.DataFrame(

reduce(lambda x, y: np.array(x) + np.array(y), delong_z)

/ len(delong_z),

index=methods,

columns=methods,

)

delong_pp1 = pd.DataFrame(

reduce(lambda x, y: np.array(x) + np.array(y), delong_p)

/ len(delong_p),

index=methods,

columns=methods,

)

else:

\# 如果 delong_z 为空，创建一个全空的 DataFrame 防止报错

delong_zz1 = pd.DataFrame(np.nan, index=methods, columns=methods)

delong_pp1 = pd.DataFrame(np.nan, index=methods, columns=methods)

delong_zz = delong_zz1.applymap(lambda x: round_dec(x, d=decimal_places))

delong_pp = delong_pp1.applymap(lambda x: round_dec(x, d=decimal_places))

delong_zz_name = pd.DataFrame(

list(delong_zz.index), columns=["name"], index=list(delong_zz.index)

)

delong_zz = pd.concat(

[delong_zz_name, delong_zz], axis=1, ignore_index=False

)

delong_pp_name = pd.DataFrame(

list(delong_pp.index), columns=["name"], index=list(delong_pp.index)

)

delong_pp = pd.concat(

[delong_pp_name, delong_pp], axis=1, ignore_index=False

)

if boostrap != 1:

if not df_plot.empty and df_plot["mean"].notna().any():

current_max = np.nanmax(df_plot["mean"])

current_min = np.nanmin(df_plot["mean"])

current_std_max = (

np.nanmax(df_plot["std"])

if np.nanmax(df_plot["std"]) > 0

else 0.02

)

\# 动态计算缓冲空间

padding = 0.05

ymax = current_max + current_std_max + padding

ymin = 0.0

\# 边界限制，确保不会超出 [0, 1]

ymax = min(1.0, math.ceil(ymax * 10) / 10)

ymin = 0.0

else:

ymax = 1.0

ymin = 0.0

\# 验证集ROC曲线

try:

_set_custom_legend(ax, location, fontsize=6)

\# if location in [3, 4]:

\#	plt.tight_layout()

if output_dir is not None:

jpeg_path = save_fig(

output_dir, "valid_ROC_curve", "jpeg", fig, str_time=str_time

)

fmt_path = save_fig(

output_dir, "valid_ROC_curve", image_format, fig, str_time=str_time

)

figures_list.append(

{

"name": "验证集ROC曲线",

"file": {"jpeg": jpeg_path, image_format: fmt_path},

"description": "验证集各模型ROC曲线对比。",

}

)

except Exception as e:

figures_list.append(

{

"name": "验证集ROC曲线",

"file": {},

"description": f"生成失败: {e}",

}

)

finally:

plt.close(fig)

\# 训练集 ROC 曲线与森林图

try:

df_test_auc = []

if output_dir is not None:

\# 画训练集ROC

fig1 = plt.figure(figsize=(4, 4), dpi=dpi)

\# 画对角线

plt.plot(

[0, 1],

[0, 1],

linestyle="--",

lw=1,

color="grey",

alpha=0.8,

)

for i in range(len(fpr_train_alls)):

df_test_auc.append(df_0_train.iloc[i + 1]["AUC"])

plt.plot(

fpr_train_alls[i],

tpr_train_alls[i],

lw=1.5,

alpha=0.9,

c=palette_dict[style][i],

label=name_dict[train_method_alls[i]]

+ "(AUC = "

+ df_0_train.iloc[i + 1]["AUC(95%CI)"][

: df_0_train.iloc[i + 1]["AUC(95%CI)"].find("(")

].strip()

+ sdorci

+ df_0_train.iloc[i + 1]["AUC(95%CI)"][

df_0_train.iloc[i + 1]["AUC(95%CI)"].find("(") :

].strip()

+ ")",

)

plt.xlim([-0.02, 1.02])

plt.ylim([-0.02, 1.02])

plt.xlabel("1-Specificity")

plt.ylabel("Sensitivity")

plt.title("ROC curve(Training)")

_set_custom_legend(plt, location, fontsize=6)

\# if location in [3, 4]:

\#	plt.tight_layout()

jpeg_path = save_fig(

output_dir, "ROC_Train_curve", "jpeg", fig1, str_time=str_time

)

fmt_path = save_fig(

output_dir, "ROC_Train_curve", image_format, fig1, str_time=str_time

)

figures_list.append(

{

"name": "训练集ROC曲线",

"file": {"jpeg": jpeg_path, image_format: fmt_path},

"description": "训练集各模型ROC曲线对比。",

}

)

plt.close(fig1)

if has_testset:

fig_test_roc = plt.figure(figsize=(4, 4), dpi=dpi)

plt.plot([0, 1], [0, 1], linestyle="--", lw=1, color="grey", alpha=0.8)

for i_m, m_name in enumerate(methods):

\# 获取测试集结果

row_data = df_0_test[

df_0_test["Classifications"] == name_dict[m_name]

].iloc[0]

plt.plot(

mean_fpr,

holdout_tpr_dict[m_name],

lw=1.5,

alpha=0.9,

c=palette_dict[style][i_m],

label=f"{name_dict[m_name]} (AUC = {row_data['AUC(95%CI)']})",

)

plt.xlim([-0.02, 1.02])

plt.ylim([-0.02, 1.02])

plt.xlabel("1-Specificity")

plt.ylabel("Sensitivity")

plt.title("ROC curve(Test)")

_set_custom_legend(plt, location, fontsize=6)

\# if location in [3, 4]:

\#	plt.tight_layout()

jpeg_path_test = save_fig(

output_dir, "ROC_Test_curve", "jpeg", fig_test_roc, str_time=str_time

)

fmt_path_test = save_fig(

output_dir,

"ROC_Test_curve",

image_format,

fig_test_roc,

str_time=str_time,

)

figures_list.append(

{

"name": "测试集ROC曲线",

"file": {"jpeg": jpeg_path_test, image_format: fmt_path_test},

"description": "独立测试集各模型ROC曲线对比。",

}

)

plt.close(fig_test_roc)

\# 验证集模型对比图

if boostrap != 1:

try:

model_colors = palette_dict[style][: len(methods)]

if comparison_plot_type == "forest":

comparison_paths = forest_plot(

df_input=df_plot,

name="method",

value="mean",

err="std",

direct="horizontal",

fig_size=[len(methods) + 3, 4],

ylim=[ymin, ymax],

title="Forest Plot of Each Model AUC Score ",

output_dir=output_dir,

dpi=dpi,

image_format=image_format,

colors=model_colors,

legend_location=location,

)

figure_name = "验证集多模型森林图"

figure_desc = "展示各模型验证集AUC均值及误差范围的森林图。"

else:

df_val_auc_boxplot = pd.DataFrame(val_auc_boxplot_records)

comparison_paths = multimodel_auc_boxplot(

df_input=df_val_auc_boxplot,

name="method",

value="AUC",

title="Validation AUC Boxplot of Each Model",

output_dir=output_dir,

dpi=dpi,

image_format=image_format,

colors=model_colors,

)

figure_name = "验证集多模型箱线图"

figure_desc = "展示各模型验证集AUC分布的箱线图。"

figures_list.append(

{

"name": figure_name,

"file": {

"jpeg": comparison_paths[0],

image_format: comparison_paths[1],

},

"description": figure_desc,

}

)

except Exception as e:

figures_list.append(

{

"name": "验证集多模型对比图",

"file": {},

"description": f"生成失败: {e}",

}

)

except Exception as e:

figures_list.append(

{"name": "训练集ROC曲线", "file": {}, "description": f"生成失败: {e}"}

)

finally:

if 'fig1' in locals(): plt.close(fig1)

if 'fig_test_roc' in locals(): plt.close(fig_test_roc)

\# 验证集校准曲线基于全部交叉验证折的 OOF 预测

try:

if output_dir is not None:

validation_calibration_paths, validation_calibration_desc = (

plot_multimodel_calibration_curves(

validation_calibration_dict,

colors=palette_dict[style],

name="Validation",

output_dir=output_dir,

smooth=smooth,

dpi=dpi,

image_format=image_format,

legend_location=location,

)

)

figures_list.append(

{

"name": "验证集多模型校准曲线",

"file": {

"jpeg": validation_calibration_paths[0],

image_format: validation_calibration_paths[1],

},

"description": (

"基于全部交叉验证折的 OOF 预测绘制。\n"

+ validation_calibration_desc

),

}

)

except Exception as e:

figures_list.append(

{

"name": "验证集多模型校准曲线",

"file": {},

"description": f"这个图生成失败: {str(e)}",

}

)

finally:

plt.close("all")

\# 独立测试集校准曲线基于全建模池重拟合后的最终模型预测

try:

if output_dir is not None and has_testset:

test_calibration_paths, test_calibration_desc = (

plot_multimodel_calibration_curves(

test_calibration_dict,

colors=palette_dict[style],

name="Test",

output_dir=output_dir,

smooth=smooth,

dpi=dpi,

image_format=image_format,

legend_location=location,

)

)

figures_list.append(

{

"name": "独立测试集多模型校准曲线",

"file": {

"jpeg": test_calibration_paths[0],

image_format: test_calibration_paths[1],

},

"description": (

"基于全建模池重拟合后的最终模型预测绘制。\n"

+ test_calibration_desc

),

}

)

elif not has_testset:

figures_list.append(

{

"name": "独立测试集多模型校准曲线",

"file": {},

"description": "未划分独立测试集，无法生成测试集校准曲线。",

}

)

except Exception as e:

figures_list.append(

{

"name": "独立测试集多模型校准曲线",

"file": {},

"description": f"这个图生成失败: {str(e)}",

}

)

finally:

plt.close("all")

\# 验证集 DCA 使用同一组完整 OOF 预测，确保模型间比较口径一致

try:

if output_dir is not None:

validation_dca_paths, validation_dca_desc = plot_decision_curves(

validation_dca_dict,

colors=palette_dict[style],

name="Validation",

output_dir=output_dir,

dpi=dpi,

image_format=image_format,

DCA_cut=dca_cut,

legend_location=location,

)

figures_list.append(

{

"name": "验证集DCA曲线图",

"file": {

"jpeg": validation_dca_paths[0],

image_format: validation_dca_paths[1],

},

"description": (

"基于全部交叉验证折的 OOF 预测绘制。\n"

+ validation_dca_desc

),

}

)

except Exception as e:

figures_list.append(

{

"name": "验证集DCA曲线图",

"file": {},

"description": f"这个图生成失败: {str(e)}",

}

)

finally:

plt.close("all")

\# 独立测试集 DCA 仅使用每个最终模型的一次固定测试集预测

try:

if output_dir is not None and has_testset:

test_dca_paths, test_dca_desc = plot_decision_curves(

test_dca_dict,

colors=palette_dict[style],

name="Test",

output_dir=output_dir,

dpi=dpi,

image_format=image_format,

DCA_cut=dca_cut,

legend_location=location,

)

figures_list.append(

{

"name": "独立测试集DCA曲线图",

"file": {

"jpeg": test_dca_paths[0],

image_format: test_dca_paths[1],

},

"description": (

"基于全建模池重拟合后的最终模型预测绘制。\n"

+ test_dca_desc

),

}

)

elif not has_testset:

figures_list.append(

{

"name": "独立测试集DCA曲线图",

"file": {},

"description": "未划分独立测试集，无法生成测试集 DCA。",

}

)

except Exception as e:

figures_list.append(

{

"name": "独立测试集DCA曲线图",

"file": {},

"description": f"这个图生成失败: {str(e)}",

}

)

finally:

plt.close("all")

df_train_result1 = df_0_train.drop([0])

df_val_result1 = df_0_val.drop([0])

df_test_result1 = df_0_test.drop([0])

classfier = df_train_result1.pop("Classifications")

df_train_result = df_train_result1.applymap(

lambda x: round_dec(x, d=decimal_places)

)

df_train_result.insert(0, "Classifications", classfier)

df_test_result1.pop("Classifications")

df_test_result = df_test_result1.applymap(

lambda x: round_dec(x, d=decimal_places)

)

df_test_result.insert(0, "Classifications", classfier)

df_val_result1.pop("Classifications")

df_val_result = df_val_result1.applymap(

lambda x: round_dec(x, d=decimal_places)

)

df_val_result.insert(0, "Classifications", classfier)

if "AUC(95%CI)" in df_train_result.columns:

AUC_95CI_tr = df_train_result.pop("AUC(95%CI)")

df_train_result.insert(1, "AUC(95%CI)", AUC_95CI_tr)

if "AUC(95%CI)" in df_test_result.columns:

AUC_95CI_te = df_test_result.pop("AUC(95%CI)")

df_test_result.insert(1, "AUC(95%CI)", AUC_95CI_te)

if "AUC(95%CI)" in df_val_result.columns:

AUC_95CI_val = df_val_result.pop("AUC(95%CI)")

df_val_result.insert(1, "AUC(95%CI)", AUC_95CI_val)

df_train_result["AUC_NUM_TEMP"] = df_train_result["AUC"].apply(get_numeric_auc)

df_val_result["AUC_NUM_TEMP"] = df_val_result["AUC"].apply(get_numeric_auc)

name_train_best = df_train_result.sort_values(

by="AUC_NUM_TEMP", ascending=False

).iloc[0]["Classifications"]

name_val_best = df_val_result.sort_values(

by="AUC_NUM_TEMP", ascending=False

).iloc[0]["Classifications"]

df_train_result.drop("AUC_NUM_TEMP", axis=1, inplace=True)

df_val_result.drop("AUC_NUM_TEMP", axis=1, inplace=True)

evaluation_label = "独立测试集" if has_testset else "验证集"

evaluation_df = df_test_result if has_testset else df_val_result

df_eval_tmp = evaluation_df.copy()

df_eval_tmp["AUC_num"] = df_eval_tmp["Classifications"].apply(

lambda x: get_val(evaluation_df, x)

)

max_auc_eval = df_eval_tmp["AUC_num"].max()

candidates = df_eval_tmp[

df_eval_tmp["AUC_num"] >= (max_auc_eval - 0.001)

].copy()

final_list = []

for _, row in candidates.iterrows():

m_name = row["Classifications"]

tr_auc = get_val(df_train_result, m_name)

eval_auc = row["AUC_num"]

gap = abs(tr_auc - eval_auc)

priority = (

1 if "logistic" in m_name.lower() else (2 if "SVM" in m_name else 3)

)

final_list.append(

{

"name": m_name,

"gap": gap,

"priority": priority,

"eval_auc": eval_auc,

"train_auc": tr_auc,

}

)

final_list.sort(key=lambda x: (x["gap"], x["priority"]))

winner = final_list[0]

name_best = winner["name"]

score_eval = winner["eval_auc"]

score_train = winner["train_auc"]

gap_final = winner["gap"]

if isKFold == "cross":

validation_method_desc = f"{boostrap}折交叉验证"

elif isKFold == "nest":

validation_method_desc = f"{boostrap}折嵌套交叉验证"

elif boostrap > 1:

validation_method_desc = f"{boostrap}次重复随机划分验证"

else:

validation_method_desc = "一次性随机划分验证"

if resultType == 0:

uncertainty_desc = (

f"当前结果格式为 Mean(SD)。训练集与验证集的SD根据{validation_method_desc}中各折/各次重复结果的离散程度计算；"

"独立测试集先用全部建模样本重新拟合最终模型，再在预留测试集上计算点估计，"

f"其SD基于测试集预测概率进行{test_ci_bootstrap}次分层bootstrap估计。"

)

else:

uncertainty_desc = (

f"当前结果格式为 Mean(95%CI)。训练集与验证集的非AUC指标基于{validation_method_desc}中各折/各次重复结果计算均值及95%CI，"

"AUC区间来自各折DeLong区间汇总；独立测试集先用全部建模样本重新拟合最终模型，"

"再在预留测试集上计算AUC的DeLong 95%CI，非AUC指标则基于测试集预测概率进行"

f"{test_ci_bootstrap}次分层bootstrap估计95%CI。"

)

str_result += "\n注意：\n"

if has_testset:

modeling_count = int(X_pool.shape[0])

holdout_count = int(X_test_holdout.shape[0])

modeling_ratio = round_dec(modeling_count / x.shape[0] * 100, decimal_places)

holdout_ratio = round_dec(holdout_count / x.shape[0] * 100, decimal_places)

if testSet:

str_result += (

f"本次分析在建模开始前根据标签列“{label}”中取值为“{testLabel}”的样本划分出独立测试集，"

f"测试集共{holdout_count}例（{holdout_ratio}%），其余{modeling_count}例（{modeling_ratio}%）进入模型比较流程。"

)

else:

str_result += (

"本次分析在建模开始前按设定比例划分出独立测试集，"

f"测试集共{holdout_count}例（{holdout_ratio}%），其余{modeling_count}例（{modeling_ratio}%）进入模型比较流程。"

)

str_result += (

f"训练集结果与验证集结果来自模型比较过程中的{validation_method_desc}汇总："

"训练集结果反映各模型在训练子集上的平均拟合表现，验证集结果反映各模型在内部验证阶段的平均表现，"

"是模型比较、筛选和稳健性判断的主要依据。"

)

str_result += uncertainty_desc

str_result += (

"独立测试集结果来源于建模开始前预留、且未参与最终测试性能汇总的样本，"

"用于对最终模型进行独立泛化能力验证。"

)

str_result += (

"需注意，“多模型分类-1.训练集(CV-Train)结果汇总”展示的是训练阶段的平均性能，"

"并不等同于最终使用全部可训练样本重新拟合后的单次训练结果；"

"“多模型分类-2.验证集(CV-Val)结果汇总”展示的是内部验证性能；"

"“多模型分类-3.独立测试集(Hold-out Test)结果汇总”才代表真正独立数据上的最终表现。\n"

)

else:

str_result += (

f"当前未独立划分测试集，全部样本共{x.shape[0]}例（100%）进入模型比较流程，"

f"训练集结果与验证集结果均来自{validation_method_desc}汇总。"

)

if resultType == 0:

str_result += (

f"当前结果格式为 Mean(SD)，训练集与验证集的SD根据{validation_method_desc}中各折/各次重复结果的离散程度计算。"

)

else:

str_result += (

f"当前结果格式为 Mean(95%CI)，训练集与验证集的非AUC指标基于{validation_method_desc}中各折/各次重复结果计算均值及95%CI，AUC区间来自各折DeLong区间汇总。"

)

str_result += (

"其中，训练集结果反映各模型在训练子集上的平均拟合表现，"

"验证集结果反映各模型在内部验证子集上的平均泛化表现，"

"是当前最佳模型筛选与性能比较的主要依据。"

)

str_result += (

"需注意，“多模型分类-1.训练集(CV-Train)结果汇总”展示的是训练阶段的平均性能，"

"并不等同于最终使用全部样本重新拟合后的单次训练结果；"

"“多模型分类-2.验证集(CV-Val)结果汇总”展示的是内部验证性能。"

)

str_result += (

"由于当前未设置独立测试集，结果描述中的“验证集”不等同于真正独立的测试集，"

"最终泛化能力仍建议通过独立测试集或外部数据进一步确认。\n"

)

str_result += "\n[综合评价结论:]\n"

if score_eval >= 0.99:

str_result += f"提示：{evaluation_label}发现极高得分（AUC={score_eval:.3f}）。"

if gap_final < 0.01:

str_result += "模型在各阶段表现近乎完美且极其稳健。请务必核实数据是否存在“标签泄露”（特征中含结局列）或样本量极少的情况。\n"

else:

str_result += "虽然得分极高，但伴随一定的泛化波动，建议在实际数据分析中进行更严格的外部验证。\n"

if len(final_list) > 1:

str_result += f"在{evaluation_label}表现并列第一（AUC均约 {max_auc_eval:.3f}）的多个模型中，最终推荐：**{name_best}**。\n"

if has_testset:

str_result += f"推荐理由：在性能相近的前提下，该模型的训练/测试泛化间隙最窄（仅 {gap_final:.3f}），表现最为稳健。\n"

else:

str_result += f"推荐理由：在性能相近的前提下，该模型的训练/验证差距最小（仅 {gap_final:.3f}），表现最为稳健。\n"

else:

str_result += f"最终判定最佳模型为：**{name_best}**。该模型在{evaluation_label}中表现最强（AUC={score_eval:.3f}）。\n"

\# 泛化分析

if gap_final > 0.10:

str_result += f"分析提示：{name_best} 在训练集得分为 {score_train:.3f}，存在明显的过拟合风险（差值 {gap_final:.3f}）。"

elif gap_final > 0.05:

str_result += f"分析提示：{name_best} 存在轻微过拟合倾向，实际使用时需关注其在外部数据上的稳定性。"

else:

if has_testset:

str_result += f"稳健性评价：{name_best} 的泛化表现理想，训练与测试阶段得分差距（{gap_final:.3f}）处于健康范围。"

else:

str_result += f"稳健性评价：{name_best} 的训练与验证表现接近，差距（{gap_final:.3f}）处于健康范围。"

best_row_info = evaluation_df.loc[

evaluation_df["Classifications"] == name_best

]

str_result += f"\n{name_best} 模型在“{evaluation_label}”的详细评价指标如下：\n"

for col in evaluation_df.columns:

if col not in ["Classifications", "AUC_L", "AUC_U"]:

str_result += f"\t{col}：{best_row_info.iloc[0][col]}\n"

cols_to_drop = ["AUC_L", "AUC_U", "AUC"]

df_train_result = df_train_result.drop(cols_to_drop, axis=1, errors="ignore")

df_test_result = df_test_result.drop(cols_to_drop, axis=1, errors="ignore")

df_val_result = df_val_result.drop(

cols_to_drop, axis=1, errors="ignore"

)

\# 测试集结果汇总表

if has_testset:

df_testset_result = df_test_result.copy()

if features_flag:

df_count_r = round_dec(Xtest.shape[0] / x.shape[0], decimal_places)

else:

df_count_r = round_dec(testsize, decimal_places)

if comparison_plot_type == "forest":

if isKFold in ["cross", "nest"]:

str_result += (

"\n下示森林图展示了各模型进行"

+ dependent_variable

+ "预测的ROC结果,图中的误差线为ROC均值及SD。\n"

+ "模型的ROC均值及SD的是通过"

+ str(boostrap)

+ "折交叉验证,"

+ "模型中的变量包括"

+ ",".join(independent_variable)

+ "。\n"

)

else:

str_result += (

"\n下示森林图展示了各模型进行"

+ dependent_variable

+ "预测的ROC结果,图中的误差线为ROC均值及SD。\n"

+ "模型的ROC均值及SD的是通过多次重复采样计算，重复采样次数为"

+ str(boostrap)

+ "次,"

+ "每一次重采样训练的验证集占总体样本的"

+ str(df_count_r * 100)

+ "%,训练集占"

+ str((1 - df_count_r) * 100)

+ "%,"

+ "模型中的变量包括"

+ ",".join(independent_variable)

+ "。\n"

)

else:

if isKFold in ["cross", "nest"]:

str_result += (

"\n下示箱线图展示了各模型在验证集AUC上的分布情况，箱体与散点来自"

+ str(boostrap)

+ "折交叉验证结果，模型中的变量包括"

+ ",".join(independent_variable)

+ "。其中，每个散点表示该模型在某一折验证集上的真实AUC值；散点在水平方向上的轻微错开仅用于避免重叠，不代表额外分组变量。\n"

)

else:

str_result += (

"\n下示箱线图展示了各模型在验证集AUC上的分布情况，箱体与散点来自"

+ str(boostrap)

+ "次重复采样结果，每一次重采样训练的验证集占总体样本的"

+ str(df_count_r * 100)

+ "%,训练集占"

+ str((1 - df_count_r) * 100)

+ "%,模型中的变量包括"

+ ",".join(independent_variable)

+ "。其中，每个散点表示该模型某一次重复采样验证集上的真实AUC值；散点在水平方向上的轻微错开仅用于避免重叠，不代表额外分组变量。\n"

)

if resultType == 0:

df_train_result.rename(

columns={

"AUC(95%CI)": "AUC(SD)",

"cutoff": "cutoff(SD)",

"准确度": "准确度(SD)",

"灵敏度": "灵敏度(SD)",

"特异度": "特异度(SD)",

"阳性预测值": "阳性预测值(SD)",

"阴性预测值": "阴性预测值(SD)",

"F1分数": "F1分数(SD)",

"Kappa": "Kappa(SD)",

},

inplace=True,

)

df_val_result.rename(

columns={

"AUC(95%CI)": "AUC(SD)",

"cutoff": "cutoff(SD)",

"准确度": "准确度(SD)",

"灵敏度": "灵敏度(SD)",

"特异度": "特异度(SD)",

"阳性预测值": "阳性预测值(SD)",

"阴性预测值": "阴性预测值(SD)",

"F1分数": "F1分数(SD)",

"Kappa": "Kappa(SD)",

},

inplace=True,

)

if has_testset:

df_test_result.rename(

columns={

"AUC(95%CI)": "AUC(SD)",

"cutoff": "cutoff(SD)",

"准确度": "准确度(SD)",

"灵敏度": "灵敏度(SD)",

"特异度": "特异度(SD)",

"阳性预测值": "阳性预测值(SD)",

"阴性预测值": "阴性预测值(SD)",

"F1分数": "F1分数(SD)",

"Kappa": "Kappa(SD)",

},

inplace=True,

)

df_testset_result.rename(

columns={

"AUC(95%CI)": "AUC(SD)",

"cutoff": "cutoff(SD)",

"准确度": "准确度(SD)",

"灵敏度": "灵敏度(SD)",

"特异度": "特异度(SD)",

"阳性预测值": "阳性预测值(SD)",

"阴性预测值": "阴性预测值(SD)",

"F1分数": "F1分数(SD)",

"Kappa": "Kappa(SD)",

},

inplace=True,

)

elif resultType == 1:

df_train_result.rename(

columns={

"cutoff": "cutoff(95%CI)",

"准确度": "准确度(95%CI)",

"灵敏度": "灵敏度(95%CI)",

"特异度": "特异度(95%CI)",

"阳性预测值": "阳性预测值(95%CI)",

"阴性预测值": "阴性预测值(95%CI)",

"F1分数": "F1分数(95%CI)",

"Kappa": "Kappa(95%CI)",

},

inplace=True,

)

df_val_result.rename(

columns={

"cutoff": "cutoff(95%CI)",

"准确度": "准确度(95%CI)",

"灵敏度": "灵敏度(95%CI)",

"特异度": "特异度(95%CI)",

"阳性预测值": "阳性预测值(95%CI)",

"阴性预测值": "阴性预测值(95%CI)",

"F1分数": "F1分数(95%CI)",

"Kappa": "Kappa(95%CI)",

},

inplace=True,

)

if has_testset:

df_test_result.rename(

columns={

"cutoff": "cutoff(95%CI)",

"准确度": "准确度(95%CI)",

"灵敏度": "灵敏度(95%CI)",

"特异度": "特异度(95%CI)",

"阳性预测值": "阳性预测值(95%CI)",

"阴性预测值": "阴性预测值(95%CI)",

"F1分数": "F1分数(95%CI)",

"Kappa": "Kappa(95%CI)",

},

inplace=True,

)

df_testset_result.rename(

columns={

"cutoff": "cutoff(95%CI)",

"准确度": "准确度(95%CI)",

"灵敏度": "灵敏度(95%CI)",

"特异度": "特异度(95%CI)",

"阳性预测值": "阳性预测值(95%CI)",

"阴性预测值": "阴性预测值(95%CI)",

"F1分数": "F1分数(95%CI)",

"Kappa": "Kappa(95%CI)",

},

inplace=True,

)

\# 结果表格整理

df_dict = {

"多模型分类-1.训练集(CV-Train)结果汇总": df_train_result,

"多模型分类-2.验证集(CV-Val)结果汇总": df_val_result,

}

if has_testset:

df_dict.update({"多模型分类-3.独立测试集(Hold-out Test)结果汇总": df_test_result})

if delong:

for ii in range(delong_zz.shape[0]):

delong_zz.iloc[ii, ii + 1] = "NA"

for ii in range(delong_pp.shape[0]):

delong_pp.iloc[ii, ii + 1] = "NA"

df_dict.update({"delong检测Z值均值表": delong_zz})

df_dict.update({"delong检测P值均值表": delong_pp})

###画PR曲线

if pr_curve:

try:

pr_eval_title = "PR Curve(Test)" if has_testset else "PR Curve(Validation)"

pr_eval_file_prefix = "PR_test" if has_testset else "PR_valid"

pr_eval_name = "测试集多模型PR曲线" if has_testset else "验证集多模型PR曲线"

pr_eval_desc = (

"独立测试集精度-召回（PR）曲线。"

if has_testset

else "验证集精度-召回（PR）曲线。"

)

fig = plot_precision_recall_curve(

model_train_s,

X_train_ps,

Y_train_ps,

name=name,

picname="PR Curve(Training)",

resultType=resultType,

location=location,

)

jpeg_path = save_fig(

output_dir, "PR_train", "jpeg", fig, str_time=str_time

)

fmt_path = save_fig(

output_dir, "PR_train", image_format, fig, str_time=str_time

)

figures_list.append(

{

"name": "训练集多模型PR曲线",

"file": {"jpeg": jpeg_path, image_format: fmt_path},

"description": "训练集精度-召回（PR）曲线。",

}

)

plt.close(fig)

fig = plot_precision_recall_curve(

model_train_s,

X_test_ps,

Y_test_ps,

name=name,

picname=pr_eval_title,

resultType=resultType,

location=location,

)

jpeg_path = save_fig(

output_dir, pr_eval_file_prefix, "jpeg", fig, str_time=str_time

)

fmt_path = save_fig(

output_dir, pr_eval_file_prefix, image_format, fig, str_time=str_time

)

figures_list.append(

{

"name": pr_eval_name,

"file": {"jpeg": jpeg_path, image_format: fmt_path},

"description": pr_eval_desc,

}

)

plt.close(fig)

except Exception as e:

figures_list.append(

{

"name": "多模型PR曲线",

"file": {},

"description": f"生成失败: {e}",

}

)

if searching == "auto" and auto_model == "Hyperopt" and len(hyperopt_losses_dict) > 0:

try:

\# fig_combined = plt.figure(figsize=(12, 6), dpi=dpi)

fig_combined = plt.figure(figsize=(4, 4), dpi=dpi)

ax_combined = fig_combined.add_subplot(111)

\# plt.subplots_adjust(right=0.8)

ax_combined.set_xlabel("Iterations")

ax_combined.set_ylabel("Best Loss")

ax_combined.set_title("Best Loss Over Iterations (Train)")

ax_combined.grid(False)

for model_n, mod_loss in hyperopt_losses_dict.items():

min_loss_s = min(mod_loss)

max_loss_s = max(mod_loss)

ax_combined.plot(

mod_loss,

label=f"{model_n}\n(({min_loss_s:.4f})--({max_loss_s:.4f}))"

)

_set_custom_legend(

ax_combined,

location,

framealpha=0.8,

fancybox=True,

shadow=True

)

\# if location in [3, 4]:

\#	plt.tight_layout()

if output_dir is not None:

jpeg_path = save_fig(

output_dir, "best_loss_plot_combined", "jpeg", fig_combined, str_time=str_time

)

fmt_path = save_fig(

output_dir, "best_loss_plot_combined", image_format, fig_combined, str_time=str_time

)

figures_list.append(

{

"name": "迭代最佳损失曲线（训练损失图）",

"file": {"jpeg": jpeg_path, image_format: fmt_path},

"description": "Hyperopt 自动寻参过程中的最佳损失变化曲线。",

}

)

except Exception as e:

figures_list.append(

{

"name": "迭代最佳损失曲线",

"file": {},

"description": f"生成失败: {e}",

}

)

finally:

if 'fig_combined' in locals():

plt.close(fig_combined)

\# 构建标准的 tables 列表

for table_name, df_object in df_dict.items():

tables_list.append(

{

"name": table_name,

"file": {"dataframe": df_object},

"description": f"包含 {table_name} 的详细数据。",

}

)

result_dict = {

"status": "success",

"description": str_result,

"tables": tables_list,

"figures": figures_list,

"environment": env_info,

}

return result_dict

\# 算法主体异常捕获

except Exception:

raise

\# 4. 特征筛选：Lasso 回归

import datetime

import os

import random

import warnings

import matplotlib

import numpy as np

import pandas as pd

\# 解耦导入

from core.utils.analysis_dict import analysis_dict

from core.utils.data_standardization import data_standardization

from core.utils.fea_selectutil import ReliefF, mrmr_classif

from core.utils.get_environment import get_env_info

from core.utils.round_dec import round_dec

current_time = datetime.datetime.now().strftime("%Y-%m-%d_%H-%M-%S")

matplotlib.use("AGG")

import matplotlib as mpl

import matplotlib.pyplot as plt

from core.utils.legend_utils import apply_roc_style_legend

from sklearn.decomposition import PCA

from sklearn.feature_selection import RFECV, SelectFromModel, f_classif, mutual_info_classif

from sklearn.linear_model import LassoCV, LogisticRegression

from sklearn.model_selection import StratifiedKFold

from sklearn.svm import LinearSVC

from xgboost import XGBClassifier

mpl.rcParams["axes.grid"] = False

\# 重置为默认样式

plt.style.use("default")

plt.rcParams["font.sans-serif"] = ["Times New Roman + SimSun"] # 用来正常显示中文标签

plt.rcParams["axes.unicode_minus"] = False # 用来正常显示负号)

plt.rcParams["ps.useafm"] = True

plt.rcParams["font.family"] = "sans-serif"

plt.rcParams["pdf.fonttype"] = 42

warnings.filterwarnings("ignore")

\# 获取环境信息

env_info = get_env_info(

["sklearn", "pandas", "matplotlib", "numpy", "xgboost"]

) # 检索对应import模块的版本信息

def feature_selection(

df_input,

df_selected,

dependent_variable,

independent_variable,

output_dir,

method="LassoCV",

select_num=None,

standardization=False,

dpi=600,

image_format="jpeg",

decimal_places=3,

legend_position=1,

fold=2,

crossstep=0.1,

):

"""

特征筛选：

:param df_input: dataframe 整体数据

:param df_selected: dataframe 筛选之后的数据

:param dependent_variable: str 因变量

:param independent_variable: list 因子

:param method: str 方法

:param standardization: 是否标准化

:param output_dir:保存路径

:param dpi:图像分辨率

:param image_format:图片保存格式

:param decimal_places:保留小数点位数

legend_position：图例位置 1：图像内部自适应放置 2：固定右下角 3：放置在图像外部, 4.放置图像下方

fold： 交叉验证折数：2,3,5 只有svm和xgbrfe可设置,

crossstep：步长：代表每轮投票去除的数量 ,只有svm和xgbrfe可设置，范围[0.1].

select_num:筛选出的特征数量：PCA，mRMR，ReliefF。

:return:

"""

try:

\# 初始化返回格式所需的列表

figures_list = []

tables_list = []

df_tab_dict = {} # 用于收集表格

df_tab = None # 确保 df_tab 在所有分支中都可访问

str_result = ""

result_fea, dropFeature = [], []

str_time = (

str(datetime.datetime.now().hour)

+ str(datetime.datetime.now().minute)

+ str(datetime.datetime.now().second)

)

random_number = random.randint(1, 100)

str_time = str_time + str(random_number)

\# 如果 df_selected 未被提供（即为 None），则默认使用 df_input

if df_selected is None:

df_selected = df_input

if method == "PCA" and dependent_variable is None:

df_temp = (

df_selected[independent_variable]

.dropna()

.reset_index()

.drop(columns="index")

)

else:

\# 确保 结局 不是 None

if dependent_variable is None:

raise ValueError("当 method 不为 'PCA' 时, 'group' 参数不能为空。")

df_temp = (

df_selected[independent_variable + [dependent_variable]]

.dropna()

.reset_index()

.drop(columns="index")

)

if method == "SVMREFCV" and (

len(independent_variable) > 50

or df_temp.shape[0] * df_temp.shape[1] > 100000

):

raise ValueError(

"对于特征筛选方法SVM-RFE:目前只支持特征数小于50的操作，且筛选数据量矩阵小于10w（样本数："

+ str(df_temp.shape[0])

+ "，因子数："

+ str(df_temp.shape[1])

+ "）。如需继续使用此方法请减少样本量或者减少筛选因子数量！"

)

if df_temp.shape[0] * df_temp.shape[1] > 2000000:

raise ValueError(

"对于特征筛选方法"

+ method

+ ":目前只支持筛选数据量矩阵小于200w（样本数为"

+ str(df_temp.shape[0])

+ "，因子数："

+ str(df_temp.shape[1])

+ "）。如需继续使用此方法请减少样本量或者减少筛选因子数量！"

)

if standardization:

rresult_dict = data_standardization(

df_temp, independent_variable, method="StandardScaler"

)

_, df_temp, _, _, _, _ = analysis_dict(rresult_dict)

if method == "LassoCV":

result_method = LassoCV(cv=5, random_state=42).fit(

df_temp[independent_variable], df_temp[dependent_variable]

)

coef = pd.Series(result_method.coef_, index=independent_variable)

dropFeature = list(coef[coef == 0].index)

result_fea = list(coef[coef != 0].index)

imp_coef = coef.drop(dropFeature)

str_result = (

"通过"

+ method

+ "方法共选择出"

+ str((coef != 0).sum())

+ "个的因子分别为："

+ str(result_fea)

+ ",其最优化正则参数为："

+ str(round_dec(float(result_method.alpha_), decimal_places))

+ "。"

)

if dropFeature:

df_tab = df_input.drop(dropFeature, axis=1)

else:

df_tab = df_input

\# Lasso 绘图监控

fig = None

plot_base_name = f"Lasso_{str_time}"

plot_name_jpeg = f"{plot_base_name}.jpeg"

plot_name_format = f"{plot_base_name}.{image_format}"

try:

fig = plt.figure(figsize=(6, 6), dpi=dpi)

imp_coef.plot(kind="barh")

plt.title("Coefficients in the Lasso Model")

plt.grid(False)

plt.savefig(

os.path.join(output_dir, plot_name_jpeg),

bbox_inches="tight",

format="jpeg",

dpi=dpi,

)

plt.savefig(

os.path.join(output_dir, plot_name_format),

bbox_inches="tight",

format=image_format,

dpi=dpi,

)

success_figure_info = {

"name": "Lasso模型系数",

"file": {"jpeg": plot_name_jpeg, image_format: plot_name_format},

"description": "显示Lasso模型中非零系数的特征及其大小。",

}

figures_list.append(success_figure_info)

except Exception as e:

failure_figure_info = {

"name": "Lasso模型系数",

"file": {},

"description": f"这个图生成失败: {str(e)}",

}

figures_list.append(failure_figure_info)

finally:

if fig:

plt.close(fig)

else:

plt.close("all")

elif method == "L1_Classification":

base_model = LogisticRegression(penalty='l1', solver='liblinear', random_state=42)

result_method = base_model.fit(df_temp[independent_variable], df_temp[dependent_variable])

coef = pd.Series(result_method.coef_[0], index=independent_variable)

dropFeature = list(coef[coef == 0].index)

result_fea = list(coef[coef != 0].index)

imp_coef = coef.drop(dropFeature)

str_result = (

"通过"

+ method

+ "方法共选择出"

+ str((coef != 0).sum())

+ "个的因子分别为："

+ str(result_fea)

+ "。"

)

if dropFeature:

df_tab = df_input.drop(dropFeature, axis=1)

else:

df_tab = df_input

\# L1绘图监控

fig = None

plot_base_name = f"L1_Classification_{str_time}"

plot_name_jpeg = f"{plot_base_name}.jpeg"

plot_name_format = f"{plot_base_name}.{image_format}"

try:

fig = plt.figure(figsize=(6, 6), dpi=dpi)

imp_coef.plot(kind="barh")

plt.title("Coefficients in the L1_Classification Model")

plt.grid(False)

plt.savefig(

os.path.join(output_dir, plot_name_jpeg),

bbox_inches="tight",

format="jpeg",

dpi=dpi,

)

plt.savefig(

os.path.join(output_dir, plot_name_format),

bbox_inches="tight",

format=image_format,

dpi=dpi,

)

success_figure_info = {

"name": "L1_Classification模型系数",

"file": {"jpeg": plot_name_jpeg, image_format: plot_name_format},

"description": "显示L1_Classification模型中非零系数的特征及其大小。",

}

figures_list.append(success_figure_info)

except Exception as e:

failure_figure_info = {

"name": "L1_Classification模型系数",

"file": {},

"description": f"这个图生成失败: {str(e)}",

}

figures_list.append(failure_figure_info)

finally:

if fig:

plt.close(fig)

else:

plt.close("all")

elif method == "F-test":

f_scores, p_values = f_classif(df_temp[independent_variable], df_temp[dependent_variable])

coef = pd.Series(f_scores, index=independent_variable).fillna(0)

\# 使用 select_num 进行截断

if select_num and str(select_num).isdigit():

k = min(int(select_num), len(independent_variable))

else:

k = max(1, int(len(independent_variable) * 0.9)) # 默认取前 90%

result_fea = list(coef.nlargest(k).index)

dropFeature = list(set(independent_variable) - set(result_fea))

imp_coef = coef[result_fea].sort_values(ascending=True) # 升序画图

str_result = (

"通过"

+ method

+ "方法共选择出与目标最相关的前"

+ str(len(result_fea))

+ "个因子，分别为："

+ str(result_fea)

+ "。"

)

if dropFeature:

df_tab = df_input.drop(dropFeature, axis=1)

else:

df_tab = df_input

\# F-test绘图监控

fig = None

plot_base_name = f"F_test_{str_time}"

plot_name_jpeg = f"{plot_base_name}.jpeg"

plot_name_format = f"{plot_base_name}.{image_format}"

try:

fig = plt.figure(figsize=(6, 6), dpi=dpi)

imp_coef.plot(kind="barh")

plt.title("F-Scores in the F-test Model")

plt.grid(False)

plt.savefig(

os.path.join(output_dir, plot_name_jpeg),

bbox_inches="tight",

format="jpeg",

dpi=dpi,

)

plt.savefig(

os.path.join(output_dir, plot_name_format),

bbox_inches="tight",

format=image_format,

dpi=dpi,

)

success_figure_info = {

"name": "F-test特征得分",

"file": {"jpeg": plot_name_jpeg, image_format: plot_name_format},

"description": "显示F-test中各特征的得分F-Score大小。",

}

figures_list.append(success_figure_info)

except Exception as e:

failure_figure_info = {

"name": "F-test特征得分",

"file": {},

"description": f"这个图生成失败: {str(e)}",

}

figures_list.append(failure_figure_info)

finally:

if fig:

plt.close(fig)

else:

plt.close("all")

elif method == "Mutual_Information":

mi_scores = mutual_info_classif(df_temp[independent_variable], df_temp[dependent_variable], random_state=42)

coef = pd.Series(mi_scores, index=independent_variable)

if select_num and str(select_num).isdigit():

k = min(int(select_num), len(independent_variable))

else:

k = max(1, int(len(independent_variable) * 0.9))

result_fea = list(coef.nlargest(k).index)

dropFeature = list(set(independent_variable) - set(result_fea))

imp_coef = coef[result_fea].sort_values(ascending=True)

str_result = (

"通过"

+ method

+ "方法共选择出与目标最相关的前"

+ str(len(result_fea))

+ "个因子，分别为："

+ str(result_fea)

+ "。"

)

if dropFeature:

df_tab = df_input.drop(dropFeature, axis=1)

else:

df_tab = df_input

fig = None

plot_base_name = f"Mutual_Info_{str_time}"

plot_name_jpeg = f"{plot_base_name}.jpeg"

plot_name_format = f"{plot_base_name}.{image_format}"

try:

fig = plt.figure(figsize=(6, 6), dpi=dpi)

imp_coef.plot(kind="barh")

plt.title("Mutual Information Scores")

plt.grid(False)

plt.savefig(

os.path.join(output_dir, plot_name_jpeg),

bbox_inches="tight",

format="jpeg",

dpi=dpi,

)

plt.savefig(

os.path.join(output_dir, plot_name_format),

bbox_inches="tight",

format=image_format,

dpi=dpi,

)

success_figure_info = {

"name": "互信息特征得分",

"file": {"jpeg": plot_name_jpeg, image_format: plot_name_format},

"description": "显示基于Mutual Information方法的各特征的关联得分大小。",

}

figures_list.append(success_figure_info)

except Exception as e:

failure_figure_info = {

"name": "互信息特征得分",

"file": {},

"description": f"这个图生成失败: {str(e)}",

}

figures_list.append(failure_figure_info)

finally:

if fig:

plt.close(fig)

else:

plt.close("all")

elif method == "Pearson":

\# 计算因变量和各个自变量相关性作为筛选依据

correlations = []

for col in independent_variable:

corr = df_temp[col].corr(df_temp[dependent_variable], method='pearson')

correlations.append(abs(corr) if not pd.isna(corr) else 0)

coef = pd.Series(correlations, index=independent_variable)

if select_num and str(select_num).isdigit():

k = min(int(select_num), len(independent_variable))

else:

k = max(1, int(len(independent_variable) * 0.9))

result_fea = list(coef.nlargest(k).index)

dropFeature = list(set(independent_variable) - set(result_fea))

imp_coef = coef[result_fea].sort_values(ascending=True)

str_result = (

"通过"

+ method

+ "方法计算皮尔逊相关系数绝对值，共选择出与目标最相关的前"

+ str(len(result_fea))

+ "个因子，分别为："

+ str(result_fea)

+ "。"

)

if dropFeature:

df_tab = df_input.drop(dropFeature, axis=1)

else:

df_tab = df_input

fig = None

plot_base_name = f"Pearson_{str_time}"

plot_name_jpeg = f"{plot_base_name}.jpeg"

plot_name_format = f"{plot_base_name}.{image_format}"

try:

fig = plt.figure(figsize=(6, 6), dpi=dpi)

imp_coef.plot(kind="barh")

plt.title("Absolute Pearson Correlation")

plt.grid(False)

plt.savefig(

os.path.join(output_dir, plot_name_jpeg),

bbox_inches="tight",

format="jpeg",

dpi=dpi,

)

plt.savefig(

os.path.join(output_dir, plot_name_format),

bbox_inches="tight",

format=image_format,

dpi=dpi,

)

success_figure_info = {

"name": "Pearson特征相关性",

"file": {"jpeg": plot_name_jpeg, image_format: plot_name_format},

"description": "显示计算Pearson关联绝对值的各特征的相对大小。",

}

figures_list.append(success_figure_info)

except Exception as e:

failure_figure_info = {

"name": "Pearson特征相关性",

"file": {},

"description": f"这个图生成失败: {str(e)}",

}

figures_list.append(failure_figure_info)

finally:

if fig:

plt.close(fig)

else:

plt.close("all")

elif method == "REFCV":

model = XGBClassifier(random_state=42)

rfecv = RFECV(

estimator=model,

step=crossstep,

cv=StratifiedKFold(fold),

scoring="accuracy",

)

rfecv.fit(df_temp[independent_variable], df_temp[dependent_variable])

fea_support = list(rfecv.support_)

for i in range(len(independent_variable)):

if fea_support[i]:

result_fea.append(independent_variable[i])

else:

dropFeature.append(independent_variable[i])

if rfecv.n_features_ != len(result_fea):

raise ValueError("数据处理错误，请重新运行！---RFE")

str_result = (

"通过"

+ "RFECV"

+ "方法共选择出"

+ str(rfecv.n_features_)

+ "个的因子分别为："

+ str(result_fea)

+ "。"

)

if dropFeature:

df_tab = df_input.drop(dropFeature, axis=1)

else:

df_tab = df_input

scores = rfecv.cv_results_['mean_test_score']

if 'n_features' in rfecv.cv_results_:

num_features_range = list(rfecv.cv_results_['n_features'])

else:

num_features_range = list(range(1, len(scores) + 1))

\# CV Accuracy图

best_n_features_acc = rfecv.n_features_

if best_n_features_acc in num_features_range:

best_idx_acc = num_features_range.index(best_n_features_acc)

best_score_acc = scores[best_idx_acc]

else:

best_idx_acc = np.argmax(scores)

best_n_features_acc = num_features_range[best_idx_acc]

best_score_acc = scores[best_idx_acc]

fig_acc = None

plot_base_name_acc = f"REF_Accuracy_{str_time}"

plot_name_jpeg_acc = f"{plot_base_name_acc}.jpeg"

plot_name_format_acc = f"{plot_base_name_acc}.{image_format}"

try:

fig_acc = plt.figure(figsize=(6, 6), dpi=dpi)

plt.plot(

num_features_range, scores, color="#1f77b4", label="CV Accuracy"

)

plt.xlabel("Number of Features")

plt.ylabel("Cross-validation Accuracy")

plt.grid(False)

plt.title("RFE-CV (Accuracy)")

optimal_label_acc = (

f"Best Features: {best_n_features_acc}\n"

f"Cross-validation accuracy using Best Features: {best_score_acc:.3f}"

)

plt.plot(

best_n_features_acc,

best_score_acc,

"o",

color="red",

mfc="none",

label=optimal_label_acc,

)

apply_roc_style_legend(plt.gca(), legend_location=legend_position)

plt.savefig(

os.path.join(output_dir, plot_name_jpeg_acc),

bbox_inches="tight",

format="jpeg",

dpi=dpi,

)

plt.savefig(

os.path.join(output_dir, plot_name_format_acc),

bbox_inches="tight",

format=image_format,

dpi=dpi,

)

success_figure_info = {

"name": "RFE-CV (Accuracy)",

"file": {

"jpeg": plot_name_jpeg_acc,

image_format: plot_name_format_acc,

},

"description": "RFE-CV 交叉验证正确率随特征数量变化的曲线。",

}

figures_list.append(success_figure_info)

except Exception as e:

failure_figure_info = {

"name": "RFE-CV (Accuracy)",

"file": {},

"description": f"这个图生成失败: {str(e)}",

}

figures_list.append(failure_figure_info)

finally:

if fig_acc:

plt.close(fig_acc)

else:

plt.close("all")

\# CV Error 图

error_rates = [1 - s for s in scores]

best_n_features_err = best_n_features_acc

best_idx_err = best_idx_acc

best_score_err = error_rates[best_idx_err]

str_result += (

"\n"

+ f"x (最佳特征组合数): {best_n_features_err}\n"

+ f"y (x最优时的交叉验证正确率): {best_score_acc:.3f}\n"

+ f"y (x最优时的交叉验证错误率): {best_score_err:.3f}"

)

fig_err = None

plot_base_name_err = f"REF_Error_Rate_{str_time}"

plot_name_jpeg_err = f"{plot_base_name_err}.jpeg"

plot_name_format_err = f"{plot_base_name_err}.{image_format}"

try:

fig_err = plt.figure(figsize=(6, 6), dpi=dpi)

plt.plot(

num_features_range, error_rates, color="#1f77b4", label="CV Error"

)

plt.xlabel("Number of Features")

plt.ylabel("Cross-validation error rate")

plt.grid(False)

plt.title("RFE-CV (Error Rate)")

optimal_label_err = (

f"Best Features: {best_n_features_err}\n"

f"Cross-validation error rate using Best Features): {best_score_err:.3f}"

)

plt.plot(

best_n_features_err,

best_score_err,

"o",

color="red",

mfc="none",

label=optimal_label_err,

)

if len(error_rates) > 0:

baseline_y = error_rates[0]

x_min, x_max = plt.xlim()

plt.plot(

[x_min, x_max],

[baseline_y, baseline_y],

color="black",

linestyle=":",

linewidth=0.8,

label="baseline",

)

plt.xlim(x_min, x_max)

apply_roc_style_legend(plt.gca(), legend_location=legend_position)

plt.savefig(

os.path.join(output_dir, plot_name_jpeg_err),

bbox_inches="tight",

format="jpeg",

dpi=dpi,

)

plt.savefig(

os.path.join(output_dir, plot_name_format_err),

bbox_inches="tight",

format=image_format,

dpi=dpi,

)

success_figure_info = {

"name": "RFE-CV (Error Rate)",

"file": {

"jpeg": plot_name_jpeg_err,

image_format: plot_name_format_err,

},

"description": "RFE-CV 交叉验证错误率随特征数量变化的曲线。",

}

figures_list.append(success_figure_info)

except Exception as e:

failure_figure_info = {

"name": "RFE-CV (Error Rate)",

"file": {},

"description": f"这个图生成失败: {str(e)}",

}

figures_list.append(failure_figure_info)

finally:

if fig_err:

plt.close(fig_err)

else:

plt.close("all")

elif method == "SVMREFCV":

model = LinearSVC(random_state=42, max_iter=1000)

rfecv = RFECV(

estimator=model,

step=crossstep,

cv=StratifiedKFold(fold),

scoring="accuracy",

)

rfecv.fit(df_temp[independent_variable], df_temp[dependent_variable])

fea_support = list(rfecv.support_)

for i in range(len(independent_variable)):

if fea_support[i]:

result_fea.append(independent_variable[i])

else:

dropFeature.append(independent_variable[i])

if rfecv.n_features_ != len(result_fea):

raise ValueError("数据处理错误，请重新运行！---REF")

str_result = (

"通过"

+ method

+ "方法共选择出"

+ str(rfecv.n_features_)

+ "个的因子分别为："

+ str(result_fea)

+ "。"

)

if dropFeature:

df_tab = df_input.drop(dropFeature, axis=1)

else:

df_tab = df_input

scores = rfecv.cv_results_['mean_test_score']

if 'n_features' in rfecv.cv_results_:

num_features_range = list(rfecv.cv_results_['n_features'])

else:

num_features_range = list(range(1, len(scores) + 1))

\# CV Accuracy图

best_n_features_acc = rfecv.n_features_

if best_n_features_acc in num_features_range:

best_idx_acc = num_features_range.index(best_n_features_acc)

best_score_acc = scores[best_idx_acc]

else:

best_idx_acc = np.argmax(scores)

best_n_features_acc = num_features_range[best_idx_acc]

best_score_acc = scores[best_idx_acc]

fig_acc_svm = None

plot_base_name_acc_svm = f"SVM_REF_Accuracy_{str_time}"

plot_name_jpeg_acc_svm = f"{plot_base_name_acc_svm}.jpeg"

plot_name_format_acc_svm = f"{plot_base_name_acc_svm}.{image_format}"

try:

fig_acc_svm = plt.figure(figsize=(6, 6), dpi=dpi)

plt.plot(

num_features_range, scores, color="#1f77b4", label="CV Accuracy"

)

plt.xlabel("Number of Features")

plt.ylabel("Cross-validation Accuracy")

plt.grid(False)

plt.title("SVM-RFE-CV (Accuracy)")

optimal_label_acc = (

f"Best Features: {best_n_features_acc}\n"

f"Cross-validation accuracy using Best Features: {best_score_acc:.3f}"

)

plt.plot(

best_n_features_acc,

best_score_acc,

"o",

color="red",

mfc="none",

label=optimal_label_acc,

)

apply_roc_style_legend(plt.gca(), legend_location=legend_position)

plt.savefig(

os.path.join(output_dir, plot_name_jpeg_acc_svm),

bbox_inches="tight",

format="jpeg",

dpi=dpi,

)

plt.savefig(

os.path.join(output_dir, plot_name_format_acc_svm),

bbox_inches="tight",

format=image_format,

dpi=dpi,

)

success_figure_info = {

"name": "SVM-RFE-CV (Accuracy)",

"file": {

"jpeg": plot_name_jpeg_acc_svm,

image_format: plot_name_format_acc_svm,

},

"description": "SVM-RFE-CV 交叉验证正确率随特征数量变化的曲线。",

}

figures_list.append(success_figure_info)

except Exception as e:

failure_figure_info = {

"name": "SVM-RFE-CV (Accuracy)",

"file": {},

"description": f"这个图生成失败: {str(e)}",

}

figures_list.append(failure_figure_info)

finally:

if fig_acc_svm:

plt.close(fig_acc_svm)

else:

plt.close("all")

\# CV Error图

error_rates = [1 - s for s in scores]

best_n_features_err = best_n_features_acc

best_idx_err = best_idx_acc

best_score_err = error_rates[best_idx_err]

str_result += (

"\n"

+ f"x (最佳特征组合数): {best_n_features_err}\n"

+ f"y (x最优时的交叉验证正确率): {best_score_acc:.3f}\n"

+ f"y (x最优时的交叉验证错误率): {best_score_err:.3f}"

)

fig_err_svm = None

plot_base_name_err_svm = f"SVM_REF_Error_Rate_{str_time}"

plot_name_jpeg_err_svm = f"{plot_base_name_err_svm}.jpeg"

plot_name_format_err_svm = f"{plot_base_name_err_svm}.{image_format}"

try:

fig_err_svm = plt.figure(figsize=(6, 6), dpi=dpi)

plt.plot(

num_features_range, error_rates, color="#1f77b4", label="CV Error"

)

plt.xlabel("Number of Features")

plt.ylabel("Cross-validation error rate")

plt.grid(False)

plt.title("SVM-RFE-CV (Error Rate)")

optimal_label_err = (

f"Best Features: {best_n_features_err}\n"

f"Cross-validation error rate using Best Features): {best_score_err:.3f}"

)

plt.plot(

best_n_features_err,

best_score_err,

"o",

color="red",

mfc="none",

label=optimal_label_err,

)

if len(error_rates) > 0:

baseline_y = error_rates[0]

x_min, x_max = plt.xlim()

plt.plot(

[x_min, x_max],

[baseline_y, baseline_y],

color="black",

linestyle=":",

linewidth=0.8,

label="baseline",

)

plt.xlim(x_min, x_max)

apply_roc_style_legend(plt.gca(), legend_location=legend_position)

plt.savefig(

os.path.join(output_dir, plot_name_jpeg_err_svm),

bbox_inches="tight",

format="jpeg",

dpi=dpi,

)

plt.savefig(

os.path.join(output_dir, plot_name_format_err_svm),

bbox_inches="tight",

format=image_format,

dpi=dpi,

)

success_figure_info = {

"name": "SVM-RFE-CV (Error Rate)",

"file": {

"jpeg": plot_name_jpeg_err_svm,

image_format: plot_name_format_err_svm,

},

"description": "SVM-RFE-CV 交叉验证错误率随特征数量变化的曲线。",

}

figures_list.append(success_figure_info)

except Exception as e:

failure_figure_info = {

"name": "SVM-RFE-CV (Error Rate)",

"file": {},

"description": f"这个图生成失败: {str(e)}",

}

figures_list.append(failure_figure_info)

finally:

if fig_err_svm:

plt.close(fig_err_svm)

else:

plt.close("all")

elif method == "PCA":

if (not select_num) or select_num == "":

pca = PCA(n_components=0.9, random_state=42)

else:

if (

len(independent_variable) >= int(select_num) >= 1

):

pca = PCA(n_components=int(select_num))

else:

raise ValueError(

"因子数目应在1到"

+ str(len(independent_variable))

+ "之间，请重新输入筛选的因子数目！"

)

pca.fit(df_temp[independent_variable])

result_value = pca.transform(np.array(df_input[independent_variable]))

pca1 = PCA(n_components=len(independent_variable), random_state=42)

pca1.fit(df_temp[independent_variable])

dropFeature = independent_variable

if dropFeature:

df_tab = df_input.drop(dropFeature, axis=1)

else:

df_tab = df_input

pca_feas = []

for i in range(pca.n_components_):

pca_feas.append("PCA" + str(i + 1))

result_fea = pca_feas

df_pca_loadings = pd.DataFrame(

pca.components_, index=pca_feas, columns=independent_variable

)

df_pca_loadings = (

df_pca_loadings.round(decimal_places)

.reset_index()

.rename(columns={"index": "主成分"})

)

df_tab_dict["PCA载荷矩阵"] = df_pca_loadings

df_result = pd.DataFrame(result_value, columns=pca_feas)

df_tab = pd.concat([df_tab, df_result], axis=1)

if (not select_num) or select_num == "":

str_result = (

"通过"

+ method

+ "方法当主成分占比90%以上时，因子降维为"

+ str(pca.n_components_)

+ "维，其新的因子为："

+ str(pca_feas)

+ "。"

)

else:

str_result = (

"通过"

+ method

+ "方法当降维为"

+ str(select_num)

+ "时"

+ "，其新的因子为："

+ str(pca_feas)

+ "。"

)

fig_pca1 = None

plot_base_name_pca1 = f"PCA_Scree_{str_time}"

plot_name_jpeg_pca1 = f"{plot_base_name_pca1}.jpeg"

plot_name_format_pca1 = f"{plot_base_name_pca1}.{image_format}"

try:

fig_pca1 = plt.figure(figsize=(6, 6), dpi=dpi)

plt.title("Scree Plot")

plt.xlabel("'Factors")

plt.ylabel("Eigenvalue")

plt.plot(

range(1, len(independent_variable) + 1), pca1.explained_variance_

)

plt.grid(False)

plt.savefig(

os.path.join(output_dir, plot_name_jpeg_pca1),

bbox_inches="tight",

format="jpeg",

dpi=dpi,

)

plt.savefig(

os.path.join(output_dir, plot_name_format_pca1),

bbox_inches="tight",

format=image_format,

dpi=dpi,

)

success_figure_info = {

"name": "PCA 碎石图",

"file": {

"jpeg": plot_name_jpeg_pca1,

image_format: plot_name_format_pca1,

},

"description": "显示PCA各主成分的特征值（Eigenvalue）。",

}

figures_list.append(success_figure_info)

except Exception as e:

failure_figure_info = {

"name": "PCA 碎石图",

"file": {},

"description": f"这个图生成失败: {str(e)}",

}

figures_list.append(failure_figure_info)

finally:

if fig_pca1:

plt.close(fig_pca1)

else:

plt.close("all")

if dependent_variable is not None:

import scikitplot as skplt

plot_base_name_pca2 = "PCA_2D_plot_" + str_time

plot_name_jpeg_pca2 = f"{plot_base_name_pca2}.jpeg"

plot_name_format_pca2 = f"{plot_base_name_pca2}.{image_format}"

try:

skplt.decomposition.plot_pca_2d_projection(

pca1, df_temp[independent_variable], df_temp[dependent_variable]

)

plt.savefig(

os.path.join(output_dir, plot_name_jpeg_pca2),

dpi=dpi,

format="jpeg",

bbox_inches="tight",

)

plt.savefig(

os.path.join(output_dir, plot_name_format_pca2),

dpi=dpi,

format=image_format,

bbox_inches="tight",

)

success_figure_info = {

"name": "PCA 2D 散点图",

"file": {

"jpeg": plot_name_jpeg_pca2,

image_format: plot_name_format_pca2,

},

"description": "显示PCA降维到2D后按组（group）着色的散点图。",

}

figures_list.append(success_figure_info)

except Exception as e:

failure_figure_info = {

"name": "PCA 2D 散点图",

"file": {},

"description": f"这个图生成失败: {str(e)}",

}

figures_list.append(failure_figure_info)

finally:

plt.close("all")

elif method == "mRMR":

score_features, _, _, score = mrmr_classif(

df_temp,

independent_variable=independent_variable,

dependent_variable=dependent_variable,

max_K=len(independent_variable),

return_scores=True,

)

if (not select_num) or select_num == "":

result_fea = mrmr_classif(

df_temp,

independent_variable=independent_variable,

dependent_variable=dependent_variable,

max_K=score.index(max(score)) + 1,

return_scores=False,

)

else:

if (

len(independent_variable) >= int(select_num) >= 1

):

result_fea = mrmr_classif(

df_temp,

independent_variable=independent_variable,

dependent_variable=dependent_variable,

max_K=int(select_num),

return_scores=False,

)

else:

raise ValueError(

"因子数目应在1到"

+ str(len(independent_variable))

+ "之间，请重新输入筛选的因子数目！"

)

dropFeature = [f for f in score_features if f not in result_fea]

if dropFeature:

df_tab = df_input.drop(dropFeature, axis=1)

else:

df_tab = df_input

str_result = (

"通过"

+ method

+ "方法共选择出"

+ str(len(result_fea))

+ "个的因子分别为："

+ str(result_fea)

+ "。"

)

fig_mrmr = None

plot_base_name_mrmr = f"mRMR_{str_time}"

plot_name_jpeg_mrmr = f"{plot_base_name_mrmr}.jpeg"

plot_name_format_mrmr = f"{plot_base_name_mrmr}.{image_format}"

try:

fig_mrmr = plt.figure(figsize=(6, 6), dpi=dpi)

plt.xlabel("Number of independent_variable selected")

plt.ylabel("mRMR Score")

plt.plot(range(1, len(independent_variable) + 1), score)

plt.grid(False)

plt.savefig(

os.path.join(output_dir, plot_name_jpeg_mrmr),

bbox_inches="tight",

format="jpeg",

dpi=dpi,

)

plt.savefig(

os.path.join(output_dir, plot_name_format_mrmr),

bbox_inches="tight",

format=image_format,

dpi=dpi,

)

success_figure_info = {

"name": "mRMR 得分图",

"file": {

"jpeg": plot_name_jpeg_mrmr,

image_format: plot_name_format_mrmr,

},

"description": "mRMR 得分随特征数量变化的曲线。",

}

figures_list.append(success_figure_info)

except Exception as e:

failure_figure_info = {

"name": "mRMR 得分图",

"file": {},

"description": f"这个图生成失败: {str(e)}",

}

figures_list.append(failure_figure_info)

finally:

if fig_mrmr:

plt.close(fig_mrmr)

else:

plt.close("all")

elif method == "ReliefF":

score = []

ff_features = []

if (not select_num) or select_num == "":

fs = ReliefF(n_features_to_keep=len(independent_variable))

fs.fit(

np.array(df_temp[independent_variable]),

np.array(df_temp[dependent_variable]),

)

for i in fs.top_features:

score.append(fs.feature_scores[i])

ff_features.append(independent_variable[i])

for fs_idx in range(len(score)):

if score[fs_idx] >= 0:

result_fea.append(ff_features[fs_idx])

else:

if (

len(independent_variable) >= int(select_num) >= 1

):

fs = ReliefF(n_features_to_keep=int(select_num))

fs.fit(

np.array(df_temp[independent_variable]),

np.array(df_temp[dependent_variable]),

)

for i in fs.top_features:

score.append(fs.feature_scores[i])

ff_features.append(independent_variable[i])

result_fea = ff_features[0 : int(select_num)]

else:

raise ValueError(

"因子数目应在1到"

+ str(len(independent_variable))

+ "之间，请重新输入筛选的因子数目！"

)

dropFeature = [f for f in ff_features if f not in result_fea]

if dropFeature:

df_tab = df_input.drop(dropFeature, axis=1)

else:

df_tab = df_input

str_result = (

"通过"

+ method

+ "方法共选择出"

+ str(len(result_fea))

+ "个的因子分别为："

+ str(result_fea)

+ "。"

)

fig_relief = None

plot_base_name_relief = f"ReliefF_{str_time}"

plot_name_jpeg_relief = f"{plot_base_name_relief}.jpeg"

plot_name_format_relief = f"{plot_base_name_relief}.{image_format}"

try:

fig_relief = plt.figure(figsize=(6, 6), dpi=dpi)

plt.xlabel("Number of independent_variable selected")

plt.ylabel("ReliefF Score")

plt.plot(

range(1, len(ff_features) + 1), score

) # 使用 ff_features 的长度

plt.grid(False)

plt.savefig(

os.path.join(output_dir, plot_name_jpeg_relief),

bbox_inches="tight",

format="jpeg",

dpi=dpi,

)

plt.savefig(

os.path.join(output_dir, plot_name_format_relief),

bbox_inches="tight",

format=image_format,

dpi=dpi,

)

success_figure_info = {

"name": "ReliefF 得分图",

"file": {

"jpeg": plot_name_jpeg_relief,

image_format: plot_name_format_relief,

},

"description": "ReliefF (Relieved) 特征得分图。",

}

figures_list.append(success_figure_info)

except Exception as e:

failure_figure_info = {

"name": "ReliefF 得分图",

"file": {},

"description": f"这个图生成失败: {str(e)}",

}

figures_list.append(failure_figure_info)

finally:

if fig_relief:

plt.close(fig_relief)

else:

plt.close("all")

else:

raise ValueError(f"未知的特征筛选方法: {method}。")

\# 标准化 Tables List

if df_tab is not None:

df_tab_dict["结果保存表"] = df_tab

for table_name, df_object in df_tab_dict.items():

tables_list.append(

{

"name": table_name,

"file": {"dataframe": df_object},

"description": f"包含 {table_name} 的详细数据。",

}

)

consensus_hint = (

"建议可运行多个特征筛选方法后，跳转到特征共识分析，用于进一步筛选多方法共同支持的稳定特征。"

)

if str_result:

str_result = str_result + consensus_hint

else:

str_result = "特征筛选执行完毕。" + consensus_hint

result_dict = {

"status": "success",

"description": str_result,

"feature_list": result_fea,

"tables": tables_list,

"figures": figures_list,

"environment": env_info,

}

return result_dict

except Exception:

raise

\# 5. 二分类模型：随机森林，内部验证为嵌套交叉验证

import datetime

import inspect

import os

import pickle

import random

import time

import matplotlib as mpl

mpl.use("AGG")

import matplotlib.pyplot as plt

import numpy as np

import pandas as pd

import scikitplot as skplt

import shap

from catboost import CatBoostClassifier

from core.utils.get_environment import get_env_info

from core.utils.ml.assistant import (bootstrap_test_metric_ci,

bootstrap_test_metric_sd, ci,

classification_metric_evaluate, dic2str,

make_class_metrics_dict, round_dec,

save_fig)

from core.utils.ml.GeneticSearch import GeneticSearchCV

from core.utils.ml.Hyperparameters import (BayesDefaultRange,

GeneticDefaultRange,

GridDefaultRange, HyperDefaultRange,

RandDefaultRange)

from core.utils.ml.HyperSearch import HyperoptSearchCV

from core.utils.ml.plot_conffusion_matrix import plot_conffusion_matrix_new

from core.utils.ml.plotdraw import (calculate_net_benefit,

calculate_net_benefit_from_predictions,

draw_shap_circle_plot,

draw_shap_rose_plot_new,

plot_calibration_curve,

plot_decision_curves, plot_learning_curve,

plot_shap_radar)

current_time = datetime.datetime.now().strftime("%Y-%m-%d_%H-%M-%S")

from joblib.externals.loky.backend.resource_tracker import ResourceTracker

plt.show = lambda: None

from lightgbm import LGBMClassifier

from ngboost import NGBClassifier

from scipy.interpolate import interp1d

from scipy.stats import ks_2samp

from sklearn.base import clone

from sklearn.ensemble import (AdaBoostClassifier, BaggingClassifier,

GradientBoostingClassifier,

RandomForestClassifier)

from sklearn.experimental import enable_halving_search_cv # noqa

from sklearn.linear_model import LogisticRegression

from sklearn.metrics import confusion_matrix

from sklearn.model_selection import (GridSearchCV, HalvingGridSearchCV,

HalvingRandomSearchCV, RandomizedSearchCV,

StratifiedKFold)

from sklearn.model_selection import train_test_split as TTS

from sklearn.naive_bayes import ComplementNB, GaussianNB, BernoulliNB

from sklearn.linear_model import SGDClassifier

from sklearn.discriminant_analysis import LinearDiscriminantAnalysis

from sklearn.neighbors import KNeighborsClassifier

from sklearn.neural_network import MLPClassifier

from sklearn.preprocessing import label_binarize

from sklearn.svm import SVC

from sklearn.tree import DecisionTreeClassifier

from skopt import BayesSearchCV

from xgboost import XGBClassifier

from yellowbrick.features import parallel_coordinates

from core.utils.legend_utils import apply_roc_style_legend

\# Make NGBoost sklearn-clone compatible by returning only constructor parameters.

if not getattr(NGBClassifier.get_params, "_medstat_safe_patch", False):

def _ngboost_safe_get_params(self, deep=True):

params = {}

for name, parameter in inspect.signature(type(self).__init__).parameters.items():

if name == "self":

continue

if hasattr(self, name):

params[name] = getattr(self, name)

elif parameter.default is not inspect._empty:

params[name] = parameter.default

return params

_ngboost_safe_get_params._medstat_safe_patch = True

NGBClassifier.get_params = _ngboost_safe_get_params

if not getattr(NGBClassifier.fit, "_medstat_safe_patch", False):

_ngboost_original_fit = NGBClassifier.fit

def _ngboost_safe_fit(self, X, y, *args, **kwargs):

result = _ngboost_original_fit(self, X, y, *args, **kwargs)

self.classes_ = np.unique(np.asarray(y))

return result

_ngboost_safe_fit._medstat_safe_patch = True

NGBClassifier.fit = _ngboost_safe_fit

if hasattr(NGBClassifier, "__sklearn_tags__") and not getattr(

NGBClassifier.__sklearn_tags__, "_medstat_safe_patch", False

):

_ngboost_original_sklearn_tags = NGBClassifier.__sklearn_tags__

def _ngboost_safe_sklearn_tags(self):

tags = _ngboost_original_sklearn_tags(self)

tags.estimator_type = "classifier"

return tags

_ngboost_safe_sklearn_tags._medstat_safe_patch = True

NGBClassifier.__sklearn_tags__ = _ngboost_safe_sklearn_tags

NGBClassifier._estimator_type = "classifier"

mpl.rcParams["axes.grid"] = False

plt.rcParams["font.sans-serif"] = ["Times New Roman + SimSun"] # 用来正常显示中文标签

plt.rcParams["axes.unicode_minus"] = False # 用来正常显示负号)

plt.rcParams["ps.useafm"] = True

plt.rcParams["font.family"] = "sans-serif"

plt.rcParams["pdf.fonttype"] = 42

random_model = [

"CatBoostClassifier",

"LGBMClassifier",

"NGBClassifier",

"XGBClassifier",

"XGBRegressor",

"RandomForestClassifier",

"AdaBoostClassifier",

"DecisionTreeClassifier",

"MLPClassifier",

"SVC",

"LogisticRegression",

"LinearDiscriminantAnalysis",

"BernoulliNB",

"SGDClassifier",

"LogisticRegressionCV",

"RandomForestRegressor",

"GradientBoostingClassifier",

"AdaBoostRegressor",

"LinearSVR",

"LassoCV",

"PCA",

"GaussianMixture",

"KMeans",

"SpectralClustering",

]

env_info = get_env_info(

[

"pandas",

"numpy",

"sklearn",

"matplotlib",

"scipy",

"seaborn",

"xgboost",

"lightgbm",

"catboost",

"ngboost",

"yellowbrick",

"shap",

"statsmodels",

]

)

\# 确保在使用全核寻参下不出现字节错误

def _send(self, cmd, name, rtype):

if len(name) > 512:

raise ValueError("name too long")

\# 确保 tracker 正在运行，初始化 self._fd

self.ensure_running()

msg = f"{cmd}:{name}:{rtype}\n".encode()

if self._fd is not None:

nbytes = os.write(self._fd, msg)

assert nbytes == len(msg)

else:

import warnings

warnings.warn(f"ResourceTracker _fd is None while sending {cmd}:{name}")

ResourceTracker._send = _send

def _shap_predict_proba(x, model, feature_names):

"""

SHAP 安全预测函数：保证输入有 feature names

"""

if not isinstance(x, pd.DataFrame):

x = pd.DataFrame(x, columns=feature_names)

return model.predict_proba(x)[:, 1]

def _set_legend_style(loc_option, **kwargs):

"""

统一控制图例位置的辅助函数

:param loc_option: 1=Best, 2=Lower Right, 3=Outside Right, 4=Bottom Center

:param kwargs: 传递给 plt.legend 的其他参数 (如 fontsize)

"""

return apply_roc_style_legend(plt.gca(), legend_location=loc_option, **kwargs)

def _predict_positive_probability(model, X_data):

"""

提取二分类阳性类别预测概率

model:object 已训练模型

X_data:DataFrame 特征数据

"""

if hasattr(model, "predict_proba"):

return model.predict_proba(X_data)[:, 1]

prob_pos = model.decision_function(X_data)

denom = prob_pos.max() - prob_pos.min()

if denom == 0:

denom = 1e-9

return (prob_pos - prob_pos.min()) / denom

class _FixedProbabilityPredictor:

"""

固定预测概率适配器

"""

def __init__(self, probabilities):

"""

初始化固定概率适配器

probabilities:array-like 阳性类别预测概率

"""

self.probabilities = np.asarray(probabilities, dtype=float).reshape(-1)

def predict_proba(self, X_data):

"""

返回二分类预测概率矩阵

X_data:DataFrame 特征数据

"""

if len(X_data) != self.probabilities.shape[0]:

raise ValueError("预测概率长度与输入样本数不一致。")

prob_pos = np.clip(self.probabilities, 0.0, 1.0)

return np.column_stack([1.0 - prob_pos, prob_pos])

def _plot_calibration_curve_from_predictions(

y_true,

y_prob,

model_name,

output_dir,

smooth,

image_format,

dpi,

legend_location,

decimal_places,

dataset_name,

):

"""

基于已预测概率绘制校准曲线

y_true:array-like 真实二分类标签

y_prob:array-like 阳性类别预测概率

model_name:str 模型显示名称

output_dir:str 图片保存路径

smooth:bool 是否平滑校准曲线

image_format:str 图片保存格式

dpi:int 图片分辨率

legend_location:int 图例位置

decimal_places:int 校准指标小数保留位数

dataset_name:str 数据集名称

"""

y_series = pd.Series(np.asarray(y_true).reshape(-1)).reset_index(drop=True)

prob_array = np.asarray(y_prob, dtype=float).reshape(-1)

if y_series.shape[0] == 0 or y_series.shape[0] != prob_array.shape[0]:

raise ValueError("真实标签与预测概率必须长度一致且不能为空。")

dummy_x = pd.DataFrame({"sample_index": np.arange(y_series.shape[0])})

fixed_model = _FixedProbabilityPredictor(prob_array)

return plot_calibration_curve(

fixed_model,

dummy_x,

dummy_x,

y_series,

y_series,

name=model_name,

output_dir=output_dir,

smooth=smooth,

image_format=image_format,

dpi=dpi,

istraing=False,

legend_location=legend_location,

show_calibration_metrics=True,

decimal_places=decimal_places,

dataset_name=dataset_name,

)

def _ml_metric_text(value, decimal_places):

"""

格式化机器学习分类指标数值

value:float 指标值

decimal_places:int 小数保留位数

"""

if value is None:

return "NA"

try:

if pd.isna(value):

return "NA"

return str(round_dec(float(value), d=int(decimal_places)))

except Exception:

return str(value)

def _ml_auc_text(metric_dic, std_dic, resulttype, decimal_places):

"""

生成AUC文本

metric_dic:dict 指标均值字典

std_dic:dict 指标标准差字典

resulttype:int 结果展示方式

decimal_places:int 小数保留位数

"""

auc_text = _ml_metric_text(metric_dic.get("AUC"), decimal_places)

if resulttype == 1:

auc_l = metric_dic.get("AUC_L")

auc_u = metric_dic.get("AUC_U")

if auc_l is not None and auc_u is not None and not pd.isna(auc_l) and not pd.isna(auc_u):

return (

auc_text

+ " (95%CI "

+ _ml_metric_text(auc_l, decimal_places)

+ "-"

+ _ml_metric_text(auc_u, decimal_places)

+ ")"

)

elif resulttype == 0 and std_dic is not None and "AUC" in std_dic:

return auc_text + " ± " + _ml_metric_text(std_dic.get("AUC"), decimal_places)

return auc_text

def _ml_auc_grade(auc_value):

"""

根据AUC给出模型区分能力分层

auc_value:float AUC值

"""

try:

auc_value = float(auc_value)

except Exception:

return "无法判断"

if auc_value >= 0.9:

return "优异"

if auc_value >= 0.8:

return "良好"

if auc_value >= 0.7:

return "尚可"

return "一般"

def _build_binary_classification_description(context):

"""

构建机器学习二分类分块式结果描述

context:dict 描述所需上下文信息

"""

decimal_places = int(context.get("decimal_places", 3))

method = context.get("method", "")

method_label = context.get("method_label", method)

dependent_variable = context.get("dependent_variable", "")

independent_variable = context.get("independent_variable", [])

feature_text = "、".join([str(item) for item in independent_variable])

sample_count = int(context.get("sample_count", 0))

binary = bool(context.get("binary", True))

class_counts = context.get("class_counts", [])

class_text = "；".join(

[f"类别({label})：N={count}例" for label, count in class_counts]

)

if not class_text:

class_text = "未能生成类别分布信息"

searching = context.get("searching", "default")

auto_model = context.get("auto_model", "")

param_text = str(context.get("param_text", "")).strip()

if searching == "auto":

search_text = f"本次参数选择方式为自动寻参，自动寻参方法为 {auto_model}。"

elif searching == "handle":

search_text = "本次参数选择方式为手动设定参数。"

else:

search_text = "本次参数选择方式为默认参数。"

if param_text:

search_text += "\n最终模型参数为：\n" + param_text

has_testset = bool(context.get("has_testset", False))

testset = bool(context.get("testset", False))

trainset = bool(context.get("trainset", False))

isKFold = context.get("isKFold", "")

n_splits = int(context.get("n_splits", 0))

val_method_name = context.get("val_method_name", "验证")

train_pool_n = context.get("train_pool_n")

test_n = context.get("test_n")

valid_n = context.get("valid_n")

validation_sizes = context.get("validation_sizes", [])

validation_ratio = context.get("validation_ratio")

test_ratio = context.get("test_ratio")

label = context.get("label", "")

testlabel = context.get("testlabel", "")

trainlabel = context.get("trainlabel", "")

if has_testset:

if testset:

if isKFold == "resample1" and trainset:

split_text = (

f"本次根据标签列 {label} 进行固定划分：{label}={testlabel} 的样本作为测试集，"

f"{label}={trainlabel} 的样本作为训练集，其余样本作为验证集。"

)

else:

split_text = (

f"本次根据标签列 {label} 进行固定测试集划分：{label}={testlabel} 的样本作为测试集，"

"其余样本作为训练集。"

)

else:

split_text = (

f"本次从总体样本中随机划分独立测试集，test_ratio={test_ratio}。"

)

split_text += (

f"原始划分后训练池样本量为 N={train_pool_n} 例，"

f"独立测试集样本量为 N={test_n} 例。"

)

else:

split_text = "当前未单独划分独立测试集，全部有效样本进入建模和内部验证流程。"

if isKFold in ["cross", "nest"]:

split_text += f"训练池内部使用 {n_splits} 折{val_method_name}评估模型稳定性。"

elif isKFold == "resample":

split_text += (

f"训练池内部按 validation_ratio={validation_ratio} "

f"重复随机抽取验证集，重复次数为 {n_splits} 次。"

)

elif isKFold == "resample1":

if valid_n is not None:

split_text += f"内部验证集样本量为 N={valid_n} 例。"

else:

split_text += "本次采用一次性重采样/留出验证方式。"

if validation_sizes:

min_valid, max_valid = min(validation_sizes), max(validation_sizes)

if min_valid == max_valid:

split_text += f"每次验证集样本量为 N={min_valid} 例。"

else:

split_text += f"各次验证集样本量范围为 N={min_valid}-{max_valid} 例。"

mean_train = context.get("mean_train", {})

mean_valid = context.get("mean_valid", {})

mean_test = context.get("metric_test", {})

std_train = context.get("std_train", {})

std_valid = context.get("std_valid", {})

std_test = context.get("std_test", {})

resulttype = int(context.get("resulttype", 1))

train_auc_text = _ml_auc_text(mean_train, std_train, resulttype, decimal_places)

valid_auc_text = _ml_auc_text(mean_valid, std_valid, resulttype, decimal_places)

train_acc_text = _ml_metric_text(mean_train.get("准确度"), decimal_places)

valid_acc_text = _ml_metric_text(mean_valid.get("准确度"), decimal_places)

train_text = (

f"训练集平均 AUC 为 {train_auc_text}，平均准确度为 {train_acc_text}。"

f"验证集平均 AUC 为 {valid_auc_text}，平均准确度为 {valid_acc_text}。"

f"验证集结果来自{val_method_name}过程，是模型选择和调参的主要参考依据。"

)

test_text = ""

generalization_text = ""

conclusion_auc = mean_valid.get("AUC")

conclusion_source = "验证集"

if has_testset:

test_auc_text = _ml_auc_text(mean_test, std_test, resulttype, decimal_places)

test_acc_text = _ml_metric_text(mean_test.get("准确度"), decimal_places)

test_sen_text = _ml_metric_text(mean_test.get("灵敏度"), decimal_places)

test_spe_text = _ml_metric_text(mean_test.get("特异度"), decimal_places)

test_text = (

f"最终模型在独立测试集上的 AUC 为 {test_auc_text}，准确度为 {test_acc_text}，"

f"灵敏度为 {test_sen_text}，特异度为 {test_spe_text}。"

"独立测试集未参与训练和内部验证，更适合用于判断模型最终泛化能力。"

)

conclusion_auc = mean_test.get("AUC")

conclusion_source = "独立测试集"

try:

auc_diff = float(mean_valid.get("AUC")) - float(mean_test.get("AUC"))

rel_gap = auc_diff / float(mean_test.get("AUC")) if float(mean_test.get("AUC")) != 0 else np.nan

generalization_text = (

f"验证集 AUC 与测试集 AUC 的差值为 "

f"{_ml_metric_text(auc_diff, decimal_places)}"

)

if not pd.isna(rel_gap):

generalization_text += (

f"，相对差异约为 {_ml_metric_text(rel_gap * 100, decimal_places)}%。"

)

if (

float(mean_train.get("AUC", 0)) >= 0.99

or float(mean_valid.get("AUC", 0)) >= 0.99

):

generalization_text += (

"训练集或验证集 AUC 接近完全可分，需警惕样本量偏小、信息泄漏或模型复杂度偏高。"

)

elif auc_diff > 0.05 and not pd.isna(rel_gap) and rel_gap > 0.08:

generalization_text += (

"验证集表现明显高于测试集，提示模型在不同数据划分上的稳定性不足，建议重新划分数据、降低模型复杂度或进一步调参。"

)

else:

generalization_text += "差异处于常见可接受范围内，模型在当前划分下表现出一定泛化一致性。"

except Exception:

generalization_text = "验证集与测试集之间的泛化一致性未能自动计算，请结合结果表进一步判断。"

else:

generalization_text = (

"当前未设置独立测试集，模型性能主要依据验证集结果评估；最终泛化能力仍需通过独立测试集或外部验证进一步确认。"

)

figure_names = context.get("figure_names", [])

figure_name_text = "、".join(figure_names) if figure_names else "未生成额外图表"

chart_lines = [f"本次输出图表包括：{figure_name_text}。"]

if any("ROC" in item for item in figure_names):

chart_lines.append("ROC 曲线用于展示不同阈值下灵敏度与特异度的权衡，AUC 越高表示区分能力越强。")

if any("校准" in item for item in figure_names):

chart_lines.append("校准曲线用于观察预测概率与真实发生率是否一致。")

if any("混淆" in item for item in figure_names):

chart_lines.append("混淆矩阵展示当前阈值下的真阳性、假阳性、真阴性和假阴性分布。")

if any("DCA" in item or "决策" in item for item in figure_names):

chart_lines.append("DCA 曲线用于评估不同阈值概率下模型的临床净获益。")

if any("SHAP" in item for item in figure_names):

chart_lines.append("SHAP 相关图用于解释特征对模型预测结果的贡献方向和强度。")

grade_text = _ml_auc_grade(conclusion_auc)

conclusion_text = (

f"综合当前结果，模型在{conclusion_source}上的区分能力为{grade_text}。"

)

if grade_text == "一般":

conclusion_text += "从当前结果看，模型区分能力一般，建议进一步优化模型特征、参数设置或数据划分策略。"

if has_testset:

conclusion_text += "建议优先参考独立测试集结果作为最终性能判断。"

else:

conclusion_text += "由于未设置独立测试集，建议后续补充独立测试或外部验证。"

if has_testset:

validation_source_text = (

f"本研究中，{val_method_name}的作用本质是一种模型评估方法，用于评估模型效果优劣。"

f"训练集及验证集的分析结果是通过{val_method_name}计算获得，"

"测试集性能验证基于实验初始阶段独立划分的测试集完成。"

)

else:

validation_source_text = (

f"本研究中，{val_method_name}的作用本质是一种模型评估方法，用于评估模型效果优劣。"

f"当前未独立划分测试集，训练集及验证集的分析结果均通过{val_method_name}计算获得。"

)

if has_testset and testset:

if isKFold == "resample1" and trainset:

train_confusion_source = (

f"该训练集来源于标签列定义的数据划分，其中标签{label}为{testlabel}的样本作为测试集，"

f"标签{label}为{trainlabel}的样本作为训练集，其余样本作为验证集。"

)

else:

train_confusion_source = (

f"该训练集来源于标签列定义的数据划分，其中标签{label}为{testlabel}的样本作为测试集，"

"其余样本作为训练集。"

)

elif has_testset:

train_confusion_source = "该训练集来源于原始数据按设定比例进行的一次性随机划分。"

elif isKFold == "resample1":

train_confusion_source = "该训练集来源于原始数据按设定比例进行的一次性随机划分，剩余样本作为验证集。"

else:

train_confusion_source = "该训练集来源于未独立划分测试集时纳入建模的全部样本。"

result_scope_text = (

validation_source_text

+ "本算法中“训练集混淆矩阵”所对应的训练集，是最终用于训练模型的全量训练数据。"

+ train_confusion_source

+ "训练集混淆矩阵是基于训练好的最终模型对同一训练集样本进行预测后统计得到的结果。"

+ "需注意，训练集结果汇总和验证集结果汇总反映的是交叉验证或重采样过程中的平均性能，"

+ "并不等同于训练集混淆矩阵对应的全量训练集结果。"

)

save_text = str(context.get("save_str", "")).strip()

timing_results = context.get("timing_results", {})

timing_lines = []

for step_name, duration in timing_results.items():

timing_lines.append(f"{step_name}: {float(duration):.4f} 秒")

sections = [

"入参说明：\n"

+ f"本次使用 {method} 机器学习方法进行分类建模。分类变量为 {dependent_variable}，"

+ f"纳入模型的特征变量共 {len(independent_variable)} 个：{feature_text}。"

+ ("当前任务按二分类流程进行。" if binary else "当前任务识别为多分类流程。"),

"数据概况：\n"

+ f"建模前按所选变量剔除缺失值后，有效样本量为 N={sample_count} 例。"

+ f"分类变量 {dependent_variable} 的类别分布为：{class_text}。",

"模型设定与训练过程：\n" + search_text,

"数据划分与验证策略：\n" + split_text,

"训练与验证结果：\n" + train_text,

]

if test_text:

sections.append("独立测试集结果：\n" + test_text)

sections.extend(

[

"泛化一致性判断：\n" + generalization_text,

"结果口径说明：\n" + result_scope_text,

"图表解读：\n" + "\n".join(chart_lines),

"总结结论：\n" + conclusion_text,

]

)

if save_text:

sections.append("结果保存说明：\n" + save_text)

if timing_lines:

sections.append("流程执行时间汇总：\n" + "\n".join(timing_lines))

return "\n\n".join(sections)

def ML_Classfication(

df_input,

dependent_variable,

independent_variable,

output_dir,

decimal_places=3,

validation_ratio=0.15,

test_ratio=0.15,

scoring="roc_auc",

method="RandomForestClassifier",

isKFold="nest",

n_splits=10,

explain=False,

shapSet=2,

explain_numvar=2,

explain_indices=0, # 解释的样本行

shap_catter=False,

shap_catter_feas=None,

searching="default",

auto_model="RandomizedSearchCV",

validationCurve=False,

smooth=False,

style="lancet",

dpi=600,

image_format="jpeg",

divide_testset=True,

label="LABEL", # 标签名

testset=False,

trainset=False,

modelsave=True,

datasave=False,

testlabel=2,

trainlabel=0,

randomstate=42,

resulttype=0,

shap_circle=False,

dca_cut=0,

location=1,

radar_plot=False,

shap_rose=False,

shap_sample_type="waterfall", # 新增：控制样本解释图类型，可选 "force" 或 "waterfall"

**kwargs,

):

"""

机器学习分类分析

Input:

df_input:DataFrame 输入的待处理数据

group_name:str 分组名

validation_ratio:float 验证集比例

test_ratio:float 测试集比例

isKFold:str "cross"交叉验证，nest:嵌套交叉验证 resample：重采样 resample1：无

scoring:str 目标评价指标

method:str 使用的机器学习分类方法/模型

'LogisticRegression':LogisticRegression(**kwargs),

'CatBoostClassifier':CatBoostClassifier(**kwargs),

'NGBClassifier':NGBClassifier(**kwargs),

'XGBClassifier':XGBClassifier(**kwargs),

'RandomForestClassifier':RandomForestClassifier(**kwargs),

'SVC':SVC(**kwargs),

'KNeighborsClassifier':KNeighborsClassifier(**kwargs),

n_splits:int 交叉验证的子集数目

explain:bool 是否进行模型解释

explain_numvar:int/None 需要解释的变量数；为空时默认展示全部自变量

explain_indices=0,解释的样本行

searching:str 是否进行自动寻参，默认为否 ("default", "handle", "auto")

auto_model:str 自动寻参的方法 ("RandomizedSearchCV", "GridSearchCV", "BayesSearchCV", "HalvingGridSearchCV", "HalvingRandomSearchCV", "GeneticSearchCV", "Hyperopt")

validationCurve:bool 是否绘制交叉验证/重采样折叠的 ROC 曲线

smooth:bool 是否对测试集 ROC 曲线进行平滑处理

savePath:str 图片存储路径

style:str 绘图风格 ("lancet", "nejm", "jama", "npg")

dpi:int 图片分辨率

picFormat:str 图片格式 ("jpeg", "jpeg", "pdf", "svg"等)

divide_testset:bool 是否独立划分测试集

label:str 数据集中用于划分测试集/训练集的标签列名 # 标签名

testSet:bool 当 divide_testset=True 时，是否根据 label 列划分测试集

trainSet:bool 当 testSet=True 且 isKFold='resample1' 时，是否根据 label 列划分训练集

modelSave:bool 是否保存训练好的模型

datasave:bool 是否保存带有预测结果的数据表

testLabel:int/str 测试集的标签值 # 测试集标签

trainLabel:int/str 训练集的标签值 (仅用于 testSet=True 且 isKFold='resample1') # 训练集标签

randomState:int 随机状态种子，用于复现结果

resultType:int 结果表格中置信区间/标准差的显示类型 (0: SD, 1: CI)

DCA_cut:boolean 决策曲线分析图的横坐标截断值

**kwargs:dict 使用机器学习分类方法的参数

location:图例位置,1-4对应不同位置，1：图像内部自适应放置 2：固定右下角 3：放置在图像外部, 4.放置图像下方

shap_sample_type="waterfall", 控制样本解释图类型，可选 "force" 或 "waterfall"

radar_plot：是否绘制shap雷达图

shap_rose=False,是否绘制shap玫瑰图

"""

\# 初始化返回结构

figures_list = []

tables_list = []

result_model_save = {}

if shap_catter_feas is None:

shap_catter_feas = []

try:

if explain_numvar in (None, ""):

explain_numvar = len(independent_variable) if independent_variable else 0

else:

explain_numvar = int(explain_numvar)

if explain_numvar > 0 and independent_variable:

explain_numvar = min(explain_numvar, len(independent_variable))

test_ci_bootstrap = max(int(kwargs.get("test_ci_bootstrap", 1000)), 1)

\# 配色方案

palette_dict = {

"lancet": [

"#00468BFF",

"#ED0000FF",

"#42B540FF",

"#0099B4FF",

"#925E9FFF",

"#FDAF91FF",

"#AD002AFF",

"#ADB6B6FF",

"#1B1919FF",

],

"nejm": [

"#BC3C29FF",

"#0072B5FF",

"#E18727FF",

"#20854EFF",

"#7876B1FF",

"#6F99ADFF",

"#FFDC91FF",

"#EE4C97FF",

"#BC3C29FF",

],

"jama": [

"#374E55FF",

"#DF8F44FF",

"#00A1D5FF",

"#B24745FF",

"#79AF97FF",

"#6A6599FF",

"#80796BFF",

"#374E55FF",

"#DF8F44FF",

],

"npg": [

"#E64B35FF",

"#4DBBD5FF",

"#00A087FF",

"#3C5488FF",

"#F39B7FFF",

"#8491B4FF",

"#91D1C2FF",

"#DC0000FF",

"#7E6148FF",

"#B09C85FF",

],

}

name_dict = { # 模型名称字典

"LogisticRegression": "logistic",

"CatBoostClassifier": "CatBoost",

"NGBClassifier": "NGBoost",

"XGBClassifier": "XGBoost",

"RandomForestClassifier": "RandomForest",

"LGBMClassifier": "LightGBM",

"SVC": "SVM",

"MLPClassifier": "MLP",

"GaussianNB": "GNB",

"ComplementNB": "CNB",

"AdaBoostClassifier": "AdaBoost",

"LinearDiscriminantAnalysis": "LDA",

"BernoulliNB": "BernoulliNB",

"SGDClassifier": "SGD",

"KNeighborsClassifier": "KNN",

"DecisionTreeClassifier": "DecisionTree",

"BaggingClassifier": "Bagging",

"GradientBoostingClassifier": "GBDT",

}

model_map = { # 模型映射字典

"LogisticRegression": LogisticRegression,

"CatBoostClassifier": CatBoostClassifier,

"NGBClassifier": NGBClassifier,

"XGBClassifier": XGBClassifier,

"RandomForestClassifier": RandomForestClassifier,

"LGBMClassifier": LGBMClassifier,

"SVC": SVC,

"MLPClassifier": MLPClassifier,

"GaussianNB": GaussianNB,

"ComplementNB": ComplementNB,

"AdaBoostClassifier": AdaBoostClassifier,

"LinearDiscriminantAnalysis": LinearDiscriminantAnalysis,

"BernoulliNB": BernoulliNB,

"SGDClassifier": SGDClassifier,

"KNeighborsClassifier": KNeighborsClassifier,

"DecisionTreeClassifier": DecisionTreeClassifier,

"BaggingClassifier": BaggingClassifier,

"GradientBoostingClassifier": GradientBoostingClassifier,

}

if method not in model_map:

raise ValueError(f"不支持的模型方法: {method}")

val_method_name = {

"cross": "交叉验证",

"nest": "嵌套交叉验证",

"resample": "重采样",

"resample1": "重采样",

}.get(isKFold, "指定的验证方式")

timing_results = {} ## 用于存储时间信息

str_time = (

str(datetime.datetime.now().hour)

+ str(datetime.datetime.now().minute)

+ str(datetime.datetime.now().second)

)

random_number = random.randint(1, 100)

str_time = str_time + str(random_number)

list_name = [dependent_variable]

save_str = ""

conf_dic_train, conf_dic_valid, conf_dic_test = {}, {}, {} # 用于存储置信区间

df_input = df_input.copy(deep=True)

df_source_save = df_input.copy(deep=True)

Xtest, Ytest, X_valid, Y_valid = None, None, None, None

df_save = None

metric_dic_test = {"AUC": 0, "准确度": 0}

df_test_result, cm_df = None, None

if isinstance(divide_testset, str):

divide_testset = divide_testset not in [

"0",

"False",

"false",

"否",

"无",

"不划分",

]

else:

divide_testset = bool(divide_testset)

has_testset = divide_testset

\# _____数据处理计时_____

start_time_data_prep = time.time()

if divide_testset and testset:

df_input = df_input[

independent_variable + [dependent_variable] + [label]

].dropna()

if label in independent_variable or label == dependent_variable:

raise ValueError("标签列不能在所在模型中，请重新选择数据划分标签列！")

else: # 如果未指定测试集划分标签，只选择特征列和分组列

df_input = df_input[independent_variable + [dependent_variable]].dropna()

binary = True

u = np.sort(np.unique(np.array(df_input[dependent_variable]))) #

if len(u) == 2 and set(u) != {0, 1}:

y_result = label_binarize(

df_input[dependent_variable], classes=[ii for ii in u]

)

y_result_pd = pd.DataFrame(

y_result, columns=[dependent_variable], index=df_input.index

)

df_input = pd.concat(

[df_input.drop(dependent_variable, axis=1), y_result_pd], axis=1

)

elif len(u) > 2:

if len(u) > 10:

raise ValueError("暂不允许类别数目大于10的情况。请检查因变量取值情况。")

binary = False

if scoring == "roc_auc":

scoring = scoring + "_ovo"

else:

scoring = scoring + "_macro"

\# 数据划分逻辑

if divide_testset and testset:

if isinstance(df_input[label][0], str):

testlabel = str(testlabel)

trainlabel = str(trainlabel)

df_input = df_input[

independent_variable + [dependent_variable] + [label]

].dropna()

if datasave:

df_save = df_source_save.copy()

if isKFold == "resample1" and trainset:

test_a = df_input[df_input[label] == testlabel]

train_a = df_input[df_input[label] != testlabel]

train_t = train_a[train_a[label] == trainlabel]

valid_t = train_a[train_a[label] != trainlabel]

train_alls = train_t.drop(label, axis=1)

valid_alls = valid_t.drop(label, axis=1)

test_alls = test_a.drop(label, axis=1)

Xtrain = train_alls.drop(dependent_variable, axis=1)

Ytrain = train_alls.loc[:, list_name].squeeze(axis=1)

X_valid = valid_alls.drop(dependent_variable, axis=1)

Y_valid = valid_alls.loc[:, list_name].squeeze(axis=1)

\# Xtest = test_a.drop(label, axis=1)

Xtest = test_alls.drop(dependent_variable, axis=1)

Ytest = test_a.loc[:, list_name].squeeze(axis=1)

if datasave:

df_save["Label_ML"] = np.nan

df_save.loc[list(Xtest.index), "Label_ML"] = 2

df_save.loc[list(X_valid.index), "Label_ML"] = 1

df_save.loc[list(Xtrain.index), "Label_ML"] = 0

save_str += "在对新数据进行保存中，新的数据划分标签为：Label_ML，便签数据中的2为测试集，1为验证集，0为训练集。"

else: # 如果指定了测试集划分标签，不是 resample1 模式或未指定训练集划分标签

test_a = df_input[df_input[label] == testlabel]

train_a = df_input[df_input[label] != testlabel]

train_all = train_a.drop(label, axis=1)

test_all = test_a.drop(label, axis=1)

df_input = df_input.drop(label, axis=1)

Xtrain = train_all.drop(dependent_variable, axis=1)

Ytrain = train_all.loc[:, list_name].squeeze(axis=1)

Xtest = test_all.drop(dependent_variable, axis=1)

Ytest = test_all.loc[:, list_name].squeeze(axis=1)

if datasave:

df_save["Label_ML"] = np.nan

df_save.loc[list(Xtest.index), "Label_ML"] = 1 # 测试集标签为 1

df_save.loc[list(Xtrain.index), "Label_ML"] = 0

save_str += "在对新数据进行保存中，新的数据划分标签为：Label_ML，便签数据中的1为测试集，0为训练集。"

elif divide_testset: # 如果未指定测试集划分标签 (随机划分训练集和测试集)

df_input = df_input[independent_variable + [dependent_variable]].dropna()

X = df_input.drop(dependent_variable, axis=1)

Y = df_input.loc[:, list_name].squeeze(axis=1)

X_train_temp, Xtest, Y_train_temp, Ytest = TTS(

X, Y, test_size=test_ratio, random_state=randomstate, stratify=Y

)

if isKFold == "resample1" and trainset:

\# 在临时训练集上再次分割，创建出最终的训练集和验证集

val_size_adjusted = validation_ratio / (1.0 - test_ratio)

Xtrain, X_valid, Ytrain, Y_valid = TTS(

X_train_temp,

Y_train_temp,

test_size=val_size_adjusted,

random_state=randomstate,

stratify=Y_train_temp,

)

else:

Xtrain, Ytrain = X_train_temp, Y_train_temp

if datasave:

df_save = df_source_save.copy()

df_save["Label_ML"] = np.nan

df_save.loc[list(Xtrain.index), "Label_ML"] = 0

if isKFold == "resample1": # 如果是 resample1 模式，测试集标签为 2

df_save.loc[list(Xtest.index), "Label_ML"] = 2

if X_valid is not None:

df_save.loc[list(X_valid.index), "Label_ML"] = 1

save_str += "在对新数据进行保存中，新的数据划分标签为：Label_ML，便签数据中的2为测试集，1为验证集，0为训练集。"

else:

df_save.loc[list(Xtest.index), "Label_ML"] = 1

save_str += "在对新数据进行保存中，新的数据划分标签为：Label_ML，便签数据中的1为测试集，0为训练集。"

else: # 不划分测试集，仅使用训练集和验证集

df_input = df_input[independent_variable + [dependent_variable]].dropna()

X = df_input.drop(dependent_variable, axis=1)

Y = df_input.loc[:, list_name].squeeze(axis=1)

Xtrain, Ytrain = X, Y

if datasave:

df_save = df_source_save.copy()

if isKFold == "resample1":

Xtrain, X_valid, Ytrain, Y_valid = TTS(

X,

Y,

test_size=validation_ratio,

random_state=randomstate,

stratify=Y,

)

if datasave:

df_save["Label_ML"] = np.nan

df_save.loc[list(X_valid.index), "Label_ML"] = 1

df_save.loc[list(Xtrain.index), "Label_ML"] = 0

save_str += "在对新数据进行保存中，新的数据划分标签为：Label_ML，标签数据中的1为验证集，0为训练集。"

elif datasave:

save_str += "在当前未独立划分测试集的设置下，训练集与验证集为过程内动态生成，结果保存表中不单独标记 Label_ML。"

single_split_mode = isKFold == "resample1" and (

(divide_testset and trainset) or not divide_testset

)

df_dict = {}

\# 数据预处理计算结束

end_time_data_prep = time.time()

timing_results["数据预处理与划分时间"] = (

end_time_data_prep - start_time_data_prep

)

\# 计时超参数调优

start_time_tuning = time.time()

if method == "ComplementNB":

if (Xtrain < 0).any().any():

raise ValueError(

"ComplementNB 要求输入特征必须为非负数。请检查数据是否经过了标准化(StandardScaler)处理，或原始数据中包含负数。建议对该模型使用 MinMaxScaler。"

)

if searching == "auto":

base_model_kwargs = {}

if method == "CatBoostClassifier":

base_model_kwargs.update(

{

"verbose": 0,

"allow_writing_files": False,

"loss_function": "Logloss",

"random_state": randomstate,

}

)

elif method == "NGBClassifier":

base_model_kwargs.update(

{"verbose": False, "verbose_eval": 0, "random_state": randomstate}

)

elif method == "SGDClassifier":

base_model_kwargs.update(

{"loss": "log_loss", "early_stopping": True, "random_state": randomstate}

)

elif method == "BernoulliNB":

base_model_kwargs.update({"binarize": 0.0})

\# 网格寻参和随机寻参方法重写

if auto_model == "RandomizedSearchCV": # 随机寻参

searcher = RandomizedSearchCV(

model_map[method](**base_model_kwargs),

param_distributions=RandDefaultRange[method],

random_state=randomstate,

n_jobs=-1,

cv=n_splits,

) # 添加 random_state

elif auto_model == "GridSearchCV": # 网格寻参

searcher = GridSearchCV(

model_map[method](**base_model_kwargs),

param_grid=GridDefaultRange[method],

n_jobs=-1,

cv=n_splits,

)

elif auto_model == "BayesSearchCV": # 贝叶斯寻参

searcher = BayesSearchCV(

model_map[method](**base_model_kwargs),

search_spaces=BayesDefaultRange[method],

random_state=randomstate,

n_jobs=-1,

n_iter=10,

cv=n_splits,

) # 添加 random_state

elif auto_model == "HalvingGridSearchCV": # 连续二分网格搜索

searcher = HalvingGridSearchCV(

model_map[method](**base_model_kwargs),

param_grid=GridDefaultRange[method],

random_state=randomstate,

min_resources="exhaust" if method == "NGBClassifier" else "smallest",

n_jobs=-1,

cv=n_splits,

) # 添加 random_state

elif auto_model == "HalvingRandomSearchCV": # 连续二分随机搜索

searcher = HalvingRandomSearchCV(

model_map[method](**base_model_kwargs),

param_distributions=RandDefaultRange[method],

random_state=randomstate,

min_resources="exhaust" if method == "NGBClassifier" else "smallest",

n_jobs=-1,

cv=n_splits,

) # 添加 random_state

elif auto_model == "GeneticSearchCV": # 遗传算法寻参

searcher = GeneticSearchCV(

model_map[method](**base_model_kwargs),

param_grid=GeneticDefaultRange[method],

n_generations=10,

population_size=50,

mutation_rate=0.1,

crossover_rate=0.8,

elite_size=2,

scoring=scoring,

cv=n_splits,

random_state=randomstate,

n_jobs=-1,

)

elif auto_model == "Hyperopt": # Hyperopt 算法寻参

searcher = HyperoptSearchCV(

estimator=model_map[method](**base_model_kwargs),

param_space=HyperDefaultRange[method],

max_evals=100,

scoring=scoring,

cv=n_splits,

random_state=randomstate,

n_jobs=-1,

)

searcher.fit(Xtrain, Ytrain) # 执行寻参包含了交叉验证

clf = searcher.best_estimator_

print(clf)

\# Hyperopt 绘图尝试

if auto_model == "Hyperopt":

try:

fig = searcher.plot_trials()

best_loss = abs(searcher.best_score_)

trials_count = len(searcher.trials_.trials)

hyp_desc = f"迭代优化曲线分析：\n算法共进行了 {trials_count} 次迭代尝试。曲线展示了随着迭代次数增加，模型性能的提升过程（损失函数下降或分数上升）。最终找到的最佳参数组合对应的得分为 {best_loss:.4f}。如果曲线尾部趋于平缓，说明模型已收敛。"

plot_name_temp = f"best_loss_plot_{str_time}"

pic_name_jpeg = f"{plot_name_temp}.jpeg"

pic_name_fmt = f"{plot_name_temp}.{image_format}"

fig.savefig(

os.path.join(output_dir, pic_name_jpeg),

bbox_inches="tight",

format="jpeg",

dpi=dpi,

)

fig.savefig(

os.path.join(output_dir, pic_name_fmt),

bbox_inches="tight",

format=image_format,

dpi=dpi,

)

plt.close(fig)

figures_list.append(

{

"name": "迭代最佳损失曲线",

"position": "1-1",

"file": {"jpeg": pic_name_jpeg, image_format: pic_name_fmt},

"description": hyp_desc,

}

)

except Exception as e:

figures_list.append(

{

"name": "迭代最佳损失曲线",

"file": {},

"description": f"图表生成失败: {str(e)}",

}

)

elif searching == "handle": # 如果手动指定参数

if method == "LGBMClassifier":

kwargs["verbosity"] = -1

if "verbose" in kwargs:

kwargs.pop("verbose")

if method == "CatBoostClassifier":

kwargs.setdefault("verbose", 0)

kwargs.setdefault("allow_writing_files", False)

kwargs.setdefault("loss_function", "Logloss")

elif method == "NGBClassifier":

kwargs.setdefault("verbose", False)

kwargs.setdefault("verbose_eval", 0)

elif method == "SGDClassifier":

kwargs.setdefault("loss", "log_loss")

kwargs.setdefault("early_stopping", True)

elif method == "BernoulliNB":

kwargs.setdefault("binarize", 0.0)

if method == "SVC":

kwargs["probability"] = True #

if method == "RandomForestClassifier" and kwargs.get("max_depth") == "None":

kwargs["max_depth"] = None

if method == "DecisionTreeClassifier" and kwargs.get("max_depth") == "None":

kwargs["max_depth"] = None

if method == "MLPClassifier": # 神经网络参数处理

hls_vals = str(kwargs["hidden_layer_sizes"]).split(

","

) # 获取隐藏层大小字符串并分割

hls_value = () # 初始化隐藏层大小元组

for hls_val in hls_vals:

try:

if 5 <= int(hls_val) <= 200: # 检查隐藏层宽度范围

hls_value = hls_value + (int(hls_val),)

else:

raise ValueError("请按照要求重新设置隐藏层宽度！")

except:

raise ValueError("请重新设神经网络模型中的隐藏层宽度！")

kwargs["hidden_layer_sizes"] = hls_value

if method == "GaussianNB" and kwargs.get("priors") == "None":

kwargs["priors"] = None

elif method == "GaussianNB":

pri_vals = str(kwargs["priors"]).split(",")

pri_value = ()

pri_sum = 0.0

for pri_val in pri_vals:

try:

pri_sum = float(pri_val) + pri_sum

pri_value = pri_value + (float(pri_val),)

except:

raise ValueError("请重新设朴素贝叶斯模型中的先验概率！")

if len(pri_vals) == len(Y.unique()) and abs(pri_sum - 1.0) < 1e-6:

kwargs["priors"] = pri_value

else:

raise ValueError("请重新设朴素贝叶斯模型中的先验概率！")

if method in random_model:

kwargs["random_state"] = 42

\# 获取当前模型能够接受的合法参数名列表

sig = inspect.signature(model_map[method].__init__)

has_var_keyword = any(p.kind == inspect.Parameter.VAR_KEYWORD for p in sig.parameters.values())

if has_var_keyword:

safe_kwargs = kwargs.copy()

else:

valid_params = sig.parameters.keys()

\# 过滤 kwargs，剔除不支持的参数

safe_kwargs = {k: v for k, v in kwargs.items() if k in valid_params}

clf = model_map[method](**safe_kwargs).fit(Xtrain, Ytrain)

elif searching == "default": # 如果使用默认参数

if method == "LGBMClassifier":

kwargs["verbosity"] = -1

if "verbose" in kwargs:

kwargs.pop("verbose")

if method == "CatBoostClassifier":

kwargs.setdefault("verbose", 0)

kwargs.setdefault("allow_writing_files", False)

kwargs.setdefault("loss_function", "Logloss")

kwargs.setdefault("n_estimators", 50)

elif method == "NGBClassifier":

kwargs.setdefault("verbose", False)

kwargs.setdefault("verbose_eval", 0)

kwargs.setdefault("n_estimators", 50)

elif method == "SGDClassifier":

kwargs.setdefault("loss", "log_loss")

kwargs.setdefault("early_stopping", True)

elif method == "BernoulliNB":

kwargs.setdefault("binarize", 0.0)

if method == "SVC":

kwargs["probability"] = True

elif method == "MLPClassifier":

kwargs["hidden_layer_sizes"] = (20, 10)

kwargs["max_iter"] = 20

elif method == "RandomForestClassifier":

kwargs["n_estimators"] = 20

if method in random_model:

kwargs["random_state"] = 42

sig = inspect.signature(model_map[method].__init__)

has_var_keyword = any(p.kind == inspect.Parameter.VAR_KEYWORD for p in sig.parameters.values())

if has_var_keyword:

safe_kwargs = kwargs.copy()

else:

valid_params = sig.parameters.keys()

safe_kwargs = {k: v for k, v in kwargs.items() if k in valid_params}

clf = model_map[method](**safe_kwargs).fit(Xtrain, Ytrain)

group_labels = df_input[dependent_variable].unique()

group_labels.sort()

\# 计时超参数调优

end_time_tuning = time.time()

timing_results["超参数调优及模型构建与训练时间"] = (

end_time_tuning - start_time_tuning

)

\# 学习曲线

try:

if isKFold == "resample1":

lc_splits = 5

else:

lc_splits = n_splits

plot_name_list_lc, desc = plot_learning_curve(

clf,

Xtrain,

Ytrain,

cv=lc_splits,

scoring=scoring,

output_dir=output_dir,

dpi=dpi,

image_format=image_format,

legend_location=location,

)

figures_list.append(

{

"name": "学习曲线",

"position": "1-2",

"file": {

"jpeg": plot_name_list_lc[0],

image_format: plot_name_list_lc[1],

},

"description": desc,

}

)

except Exception as e:

figures_list.append(

{

"name": "学习曲线",

"file": {},

"description": f"图表生成失败: {str(e)}",

}

)

\# 校准曲线

if has_testset:

try:

calibration_curve_name, cal_desc = plot_calibration_curve(

clf,

Xtrain,

Xtest,

Ytrain,

Ytest,

name=name_dict[method],

output_dir=output_dir,

smooth=smooth,

image_format=image_format,

dpi=dpi,

legend_location=location,

show_calibration_metrics=True,

decimal_places=decimal_places,

dataset_name="Test",

)

figures_list.append(

{

"name": "测试集校准曲线",

"position": "1-5",

"file": {

"jpeg": calibration_curve_name[0],

image_format: calibration_curve_name[1],

},

"description": cal_desc,

}

)

except Exception as e:

figures_list.append(

{

"name": "测试集校准曲线",

"file": {},

"description": f"这个图生成失败: {str(e)}",

}

)

finally:

plt.close("all")

\# 初始化 ROC 图相关

fig_roc_valid = None

if binary:

fig_roc_valid = plt.figure(figsize=(4, 4), dpi=dpi) # 验证集 ROC 曲线图

plt.plot([0, 1], [0, 1], linestyle="--", lw=1, color="r", alpha=0.8)

plt.grid(

which="major", axis="both", linestyle="-.", alpha=0.08, color="grey"

)

data_all = {}

validation_curve_y_parts = []

validation_curve_probability_parts = []

validation_curve_collect_error = ""

_, _, full_train_metrics, _ = classification_metric_evaluate(

clf, Xtrain, Ytrain, binary

)

resThreshold = full_train_metrics["cutoff"]

tprs_train, tprs_valid = [], []

fpr_train_alls, tpr_train_alls = [], []

mean_fpr = np.linspace(0, 1, 100)

list_evaluate_dic_train = make_class_metrics_dict()

list_evaluate_dic_valid = make_class_metrics_dict()

\# 计时验证

start_time_cv = time.time()

\# 交叉验证/重采样逻辑

if isKFold == "cross" or isKFold == "nest": # 交叉验证或嵌套交叉验证

KF = StratifiedKFold(

n_splits=n_splits, random_state=randomstate, shuffle=True

) # 分层 KFold 交叉验证

for i, (train_index, valid_index) in enumerate(

KF.split(Xtrain, Ytrain)

): # 遍历各折

X_train_fold, X_valid_fold = (

Xtrain.iloc[train_index],

Xtrain.iloc[valid_index],

)

Y_train_fold, Y_valid_fold = (

Ytrain.iloc[train_index],

Ytrain.iloc[valid_index],

)

data_all.update(

{

i: {

"Xtrain": X_train_fold,

"Ytrain": Y_train_fold,

"Xvalid": X_valid_fold,

"Yvalid": Y_valid_fold,

}

}

)

if isKFold == "nest": # 嵌套交叉验证

best_auc_inner = 0.0

inner_cv = StratifiedKFold(

n_splits=n_splits, random_state=randomstate, shuffle=True

)

for j, (train_index_inner, test_index_inner) in enumerate(

inner_cv.split(X_train_fold, Y_train_fold)

): # 遍历内层各折

X_train_inner, X_test_inner = (

X_train_fold.iloc[train_index_inner],

X_train_fold.iloc[test_index_inner],

)

y_train_inner, y_test_inner = (

Y_train_fold.iloc[train_index_inner],

Y_train_fold.iloc[test_index_inner],

)

model_i = clone(clf).fit(X_train_inner, y_train_inner)

_, _, metric_dic_train_i, _ = classification_metric_evaluate(

model_i, X_train_inner, y_train_inner, binary

)

_, _, metric_dic_valid_i, _ = classification_metric_evaluate(

model_i,

X_test_inner,

y_test_inner,

binary,

Threshold=metric_dic_train_i["cutoff"],

)

if metric_dic_valid_i["AUC"] > best_auc_inner:

best_auc_inner = metric_dic_valid_i["AUC"]

model_inner = model_i

resThreshold_inner = metric_dic_train_i["cutoff"]

data_all[i].update(

{"model": model_inner, "threshold": resThreshold_inner}

)

else: # 如果是重采样模式 ('resample' 或 'resample1')

if single_split_mode:

data_all.update(

{

0: {

"Xtrain": Xtrain,

"Ytrain": Ytrain,

"Xvalid": X_valid,

"Yvalid": Y_valid,

}

}

)

model = clone(clf).fit(Xtrain, Ytrain)

data_all[0].update({"model": model, "threshold": 0.5})

else:

for i in range(n_splits):

X_train_rs, X_valid_rs, Y_train_rs, Y_valid_rs = TTS(

Xtrain,

Ytrain,

test_size=validation_ratio,

random_state=randomstate + i,

)

data_all.update(

{

i: {

"Xtrain": X_train_rs,

"Ytrain": Y_train_rs,

"Xvalid": X_valid_rs,

"Yvalid": Y_valid_rs,

}

}

)

if (

isKFold == "resample1"

and datasave

and df_save is not None

and "Label_ML" in df_save.columns

):

df_save.loc[list(data_all[0]["Xvalid"].index), "Label_ML"] = 1

i = 0

for data_key, data_value in data_all.items():

X_train_iter, Y_train_iter, X_valid_iter, Y_valid_iter = (

data_value["Xtrain"],

data_value["Ytrain"],

data_value["Xvalid"],

data_value["Yvalid"],

)

if isKFold != "nest":

if not single_split_mode:

model = clone(clf).fit(X_train_iter, Y_train_iter)

fpr_train, tpr_train, metric_dic_train, _ = (

classification_metric_evaluate(

model, X_train_iter, Y_train_iter, binary

)

)

else:

model = data_all[data_key]["model"]

fpr_train, tpr_train, metric_dic_train, _ = (

classification_metric_evaluate(

model,

X_train_iter,

Y_train_iter,

binary,

Threshold=data_all[data_key]["threshold"],

)

)

fpr_valid, tpr_valid, metric_dic_valid, _ = classification_metric_evaluate(

model,

X_valid_iter,

Y_valid_iter,

binary,

Threshold=metric_dic_train["cutoff"],

)

metric_dic_valid.update({"cutoff": metric_dic_train["cutoff"]})

if binary:

try:

valid_prob_iter = _predict_positive_probability(model, X_valid_iter)

validation_curve_y_parts.append(

np.asarray(Y_valid_iter).reshape(-1)

)

validation_curve_probability_parts.append(

np.asarray(valid_prob_iter, dtype=float).reshape(-1)

)

except Exception as e:

validation_curve_collect_error = str(e)

\# 计算所有评价指标

for key in list_evaluate_dic_train.keys():

list_evaluate_dic_train[key].append(metric_dic_train[key])

list_evaluate_dic_valid[key].append(metric_dic_valid[key])

if binary:

tprs_valid.append(np.interp(mean_fpr, fpr_valid, tpr_valid))

tprs_valid[-1][0] = 0.0

if validationCurve and fig_roc_valid is not None:

try:

plt.figure(fig_roc_valid.number)

plt.plot(

fpr_valid,

tpr_valid,

lw=1,

alpha=0.4,

color=palette_dict[style][i % len(palette_dict[style])],

label=(

"ROC fold %4d (auc=%0.3f 95%%CI (%0.3f-%0.3f))"

% (

i + 1,

metric_dic_valid["AUC"],

metric_dic_valid["AUC_L"],

metric_dic_valid["AUC_U"],

)

if resulttype == 1

else "ROC fold %4d auc=%0.3f"

% (i + 1, metric_dic_valid["AUC"])

),

)

except Exception as e:

return {

"status": "error",

"description": str(e),

}

fpr_train_alls.append(fpr_train)

tpr_train_alls.append(tpr_train)

tprs_train.append(np.interp(mean_fpr, fpr_train, tpr_train))

tprs_train[-1][0] = 0.0

i += 1

\# 验证

end_time_cv = time.time()

timing_results["验证时间（交叉验证/嵌套交叉验证/重采样）"] = (

end_time_cv - start_time_cv

)

if modelsave:

modelfile_name = method + str_time + ".pkl"

modelfile_path = os.path.join(output_dir, modelfile_name)

with open(modelfile_path, "wb") as modelfile:

pickle.dump(clf, modelfile)

result_model_save["name"] = method

result_model_save["feature"] = independent_variable

result_model_save["file"] = {"pkl": modelfile_name}

if datasave:

save_str = (

"结果保存表保留原始数据中的全部列，并在原始数据后追加模型结果列；"

"因缺失值等原因未进入建模的样本，新增结果列为空。"

+ save_str

)

save_predict_index = df_input.index.intersection(df_save.index)

res_pro = clf.predict_proba(

df_save.loc[save_predict_index, independent_variable]

)

feas_Yprob = []

for i in range(res_pro.shape[1]):

feas_Yprob.append("Yprob_" + str(i) + "_" + str_time)

pd_Yprob = pd.DataFrame(

res_pro, columns=feas_Yprob, index=save_predict_index

)

df_save = pd.concat([df_save, pd_Yprob], axis=1)

if binary:

predict_label_name = "predict_label_" + str_time

df_save[predict_label_name] = np.nan

df_save.loc[save_predict_index, predict_label_name] = list(

map(

lambda x: int(x),

(res_pro[:, 1] >= resThreshold).astype(int),

)

)

save_str += (

"其中 Label_ML 为数据划分标签，不表示模型预测类别；"

f"模型预测类别保存在 {predict_label_name} 列中。"

)

df_save["Threshold" + "_" + str_time] = np.nan

df_save.loc[save_predict_index, "Threshold" + "_" + str_time] = resThreshold

df_dict.update({"结果保存表": df_save})

\# 验证集 ROC 曲线汇总绘制与保存

if binary:

try:

mean_tpr_valid = np.mean(tprs_valid, axis=0)

mean_tpr_valid[-1] = 1.0

mean_auc_valid = np.mean(list_evaluate_dic_valid["AUC"])

plt.figure(fig_roc_valid.number)

plt.plot(

mean_fpr,

mean_tpr_valid,

color=palette_dict[style][0],

lw=2,

alpha=0.8,

label=(

r"Mean (validation) ROC (auc=%0.3f 95%%CI (%0.3f-%0.3f))"

% (

mean_auc_valid,

np.mean(list_evaluate_dic_valid["AUC_L"]),

np.mean(list_evaluate_dic_valid["AUC_U"]),

)

if resulttype == 1

else r"Mean (validation) ROC (auc=%0.3f SD (%0.3f))"

% (mean_auc_valid, np.std(list_evaluate_dic_valid["AUC"]))

),

)

roc_desc = f"验证集 ROC 曲线分析：\n模型在验证集上的平均 AUC 为 {mean_auc_valid:.3f}。"

if mean_auc_valid > 0.9:

roc_desc += " 模型分类性能极其优异。"

elif mean_auc_valid > 0.8:

roc_desc += " 模型分类性能良好。"

elif mean_auc_valid > 0.7:

roc_desc += " 模型分类性能尚可。"

else:

roc_desc += " 模型分类性能一般，可能需要优化。"

plt.xlim([-0.02, 1.02])

plt.ylim([-0.02, 1.02])

plt.xlabel("1-Specificity")

plt.ylabel("Sensitivity")

plt.title("ROC curve(Validation)")

_set_legend_style(location, fontsize=5)

if output_dir is not None:

valid_roc_jpeg = save_fig(

output_dir, "ROC_curve", "jpeg", fig_roc_valid, str_time=str_time

)

valid_roc_fmt = save_fig(

output_dir,

"ROC_curve",

image_format,

fig_roc_valid,

str_time=str_time,

)

figures_list.append(

{

"name": "验证集 ROC 曲线",

"position": "2-1",

"file": {"jpeg": valid_roc_jpeg, image_format: valid_roc_fmt},

"description": roc_desc,

}

)

except Exception as e:

figures_list.append(

{

"name": "验证集 ROC 曲线",

"file": {},

"description": f"图表生成失败: {e}",

}

)

finally:

plt.close(fig_roc_valid)

mean_dic_train, stdv_dic_train = {}, {}

mean_dic_valid, stdv_dic_valid = {}, {}

stdv_dic_test = {}

for key in list_evaluate_dic_valid.keys():

mean_dic_train[key] = np.mean(list_evaluate_dic_train[key])

mean_dic_valid[key] = np.mean(list_evaluate_dic_valid[key])

if resulttype == 0:

stdv_dic_train[key] = np.std(list_evaluate_dic_train[key], axis=0)

stdv_dic_valid[key] = np.std(list_evaluate_dic_valid[key], axis=0)

elif resulttype == 1:

conf_dic_train[key] = list(ci(list_evaluate_dic_train[key]))

conf_dic_valid[key] = list(ci(list_evaluate_dic_valid[key]))

validation_auc_value = float(mean_dic_valid.get("AUC", 0))

validation_accuracy_value = float(mean_dic_valid.get("准确度", 0))

resThreshold_train = mean_dic_train["cutoff"]

if has_testset:

(

fpr_test,

tpr_test,

metric_dic_test,

df_test_result,

) = classification_metric_evaluate( # 计算测试集指标

clf, Xtest, Ytest, binary, Threshold=resThreshold # 使用最佳模型和阈值

)

metric_dic_test.update({"cutoff": resThreshold})

if resulttype == 1 and binary:

y_prob_test = clf.predict_proba(Xtest)[:, 1]

conf_dic_test = bootstrap_test_metric_ci(

Ytest,

y_prob_test,

n_bootstrap=test_ci_bootstrap,

random_state=randomstate,

threshold=resThreshold,

)

elif resulttype == 0 and binary:

y_prob_test = clf.predict_proba(Xtest)[:, 1]

stdv_dic_test = bootstrap_test_metric_sd(

Ytest,

y_prob_test,

n_bootstrap=test_ci_bootstrap,

random_state=randomstate,

threshold=resThreshold,

)

\# 混淆矩阵表格(测试集)

y_pred = clf.predict_proba(Xtest)[:, 1] # 获取测试集预测概率

y_pred[y_pred > resThreshold] = 1

y_pred[y_pred <= resThreshold] = 0

cm = confusion_matrix(Ytest, y_pred) # 计算混淆矩阵(测试集)

cm_df = pd.DataFrame(

cm,

index=[

"真实值: " + str(group_labels[0]),

"真实值: " + str(group_labels[1]),

],

columns=[

"预测值: " + str(group_labels[0]),

"预测值: " + str(group_labels[1]),

],

).T # 转置数据框

cm_df["Freq"] = cm_df.sum(axis=1)

cm_df = cm_df.reset_index().rename(columns={"index": "预测值"})

\# 混淆矩阵表格(训练集)

train_cutoff_numeric = np.mean(list_evaluate_dic_train["cutoff"])

y_pred_train = clf.predict_proba(Xtrain)[:, 1]

y_pred_train_classified = (y_pred_train > train_cutoff_numeric).astype(int)

cm_train = confusion_matrix(Ytrain, y_pred_train_classified)

cm_df_train = pd.DataFrame(

cm_train,

index=[

"真实值: " + str(group_labels[0]),

"真实值: " + str(group_labels[1]),

],

columns=[

"预测值: " + str(group_labels[0]),

"预测值: " + str(group_labels[1]),

],

).T

cm_df_train["Freq"] = cm_df_train.sum(axis=1)

cm_df_train = cm_df_train.reset_index().rename(columns={"index": "预测值"})

\# 画训练集 ROC

if binary:

try:

fig_roc_train = plt.figure(figsize=(4, 4), dpi=dpi)

plt.plot([0, 1], [0, 1], linestyle="--", lw=1, color="r", alpha=0.8)

plt.grid(

which="major", axis="both", linestyle="-.", alpha=0.08, color="grey"

)

if validationCurve:

for i in range(len(tpr_train_alls)):

plt.plot(

fpr_train_alls[i],

tpr_train_alls[i],

lw=1,

color=palette_dict[style][i % len(palette_dict[style])],

alpha=0.4,

label=(

"ROC fold %4d (auc=%0.3f 95%%CI (%0.3f-%0.3f)) "

% (

i + 1,

list_evaluate_dic_train["AUC"][i],

list_evaluate_dic_train["AUC_L"][i],

list_evaluate_dic_train["AUC_U"][i],

)

if resulttype == 1

else "ROC fold %4d auc=%0.3f "

% (i + 1, list_evaluate_dic_train["AUC"][i])

),

)

mean_tpr_train = np.mean(tprs_train, axis=0)

mean_tpr_train[-1] = 1.0

mean_auc_train = np.mean(list_evaluate_dic_train["AUC"])

plt.plot(

mean_fpr,

mean_tpr_train,

color=palette_dict[style][0],

lw=1.8,

alpha=0.7,

label=(

r"Mean (train) ROC (auc=%0.3f 95%%CI (%0.3f-%0.3f))"

% (

mean_auc_train,

np.mean(list_evaluate_dic_train["AUC_L"]),

np.mean(list_evaluate_dic_train["AUC_U"]),

)

if resulttype == 1

else "Mean (train) ROC (auc=%0.3f SD (%0.3f))"

% (mean_auc_train, np.std(list_evaluate_dic_train["AUC"]))

),

)

plt.xlim([-0.02, 1.02])

plt.ylim([-0.02, 1.02])

plt.xlabel("1-Specificity")

plt.ylabel("Sensitivity")

plt.title("ROC curve(Training)")

_set_legend_style(location, fontsize=5)

train_roc_desc = f"训练集 ROC 曲线：\n模型在训练集上的平均 AUC 为 {mean_auc_train:.3f}。通常训练集性能高于验证集，如果两者差距过大则提示过拟合风险。"

if output_dir is not None:

train_roc_jpeg = save_fig(

output_dir,

"ROC_curve_train",

"jpeg",

fig_roc_train,

str_time=str_time,

)

train_roc_fmt = save_fig(

output_dir,

"ROC_curve_train",

image_format,

fig_roc_train,

str_time=str_time,

)

figures_list.append(

{

"name": "训练集 ROC 曲线",

"position": "2-2",

"file": {"jpeg": train_roc_jpeg, image_format: train_roc_fmt},

"description": train_roc_desc,

}

)

except Exception as e:

figures_list.append(

{

"name": "训练集 ROC 曲线",

"file": {},

"description": f"图表生成失败: {str(e)}",

}

)

finally:

plt.close(fig_roc_train)

if has_testset:

\# 画测试集 ROC

try:

fig_roc_test = plt.figure(figsize=(4, 4), dpi=dpi)

plt.plot([0, 1], [0, 1], linestyle="--", lw=1, color="r", alpha=0.8)

plt.grid(

which="major", axis="both", linestyle="-.", alpha=0.08, color="grey"

)

if smooth:

tpr_test_unique, tpr_test_index = np.unique(

fpr_test, return_index=True

)

fpr_test_new = np.linspace(

min(fpr_test), max(fpr_test), len(fpr_test)

)

f = interp1d(

tpr_test_unique, tpr_test[tpr_test_index], kind="linear"

)

tpr_test_new = f(fpr_test_new)

else:

fpr_test_new = fpr_test

tpr_test_new = tpr_test

plt.plot(

fpr_test_new,

tpr_test_new,

lw=1.5,

alpha=0.6,

color=palette_dict[style][0],

label=(

"Test Set ROC (auc=%0.3f 95%%CI (%0.3f-%0.3f)) "

% (

metric_dic_test["AUC"],

metric_dic_test["AUC_L"],

metric_dic_test["AUC_U"],

)

if resulttype == 1

else "Test Set ROC auc=%0.3f" % (metric_dic_test["AUC"])

),

)

plt.xlim([-0.02, 1.02])

plt.ylim([-0.02, 1.02])

plt.xlabel("1-Specificity")

plt.ylabel("Sensitivity")

plt.title("ROC curve(Test)")

_set_legend_style(location, fontsize=5)

test_auc_val = metric_dic_test["AUC"]

test_roc_desc = f"测试集 ROC 曲线：\n在完全独立的测试集上，模型 AUC 达到 {test_auc_val:.3f}。这是衡量模型最终泛化能力的金标准。"

if output_dir is not None:

test_roc_jpeg = save_fig(

output_dir,

"ROC_curve_test",

"jpeg",

fig_roc_test,

str_time=str_time,

)

test_roc_fmt = save_fig(

output_dir,

"ROC_curve_test",

image_format,

fig_roc_test,

str_time=str_time,

)

figures_list.append(

{

"name": "测试集 ROC 曲线",

"position": "2-3",

"file": {"jpeg": test_roc_jpeg, image_format: test_roc_fmt},

"description": test_roc_desc,

}

)

except Exception as e:

figures_list.append(

{

"name": "测试集 ROC 曲线",

"file": {},

"description": f"图表生成失败: {str(e)}",

}

)

finally:

plt.close(fig_roc_test)

if has_testset:

df_test_result = df_test_result.map(

lambda x: round_dec(x, d=decimal_places)

)

description_mean_train = mean_dic_train.copy()

description_mean_valid = mean_dic_valid.copy()

description_metric_test = metric_dic_test.copy()

description_std_train = stdv_dic_train.copy()

description_std_valid = stdv_dic_valid.copy()

description_std_test = stdv_dic_test.copy()

if resulttype == 1:

for tem in ["AUC", "AUC_L", "AUC_U"]:

if tem in mean_dic_train and tem != "AUC":

del mean_dic_train[tem]

if tem in mean_dic_valid and tem != "AUC":

del mean_dic_valid[tem]

if tem in conf_dic_train and tem != "AUC":

del conf_dic_train[tem]

if tem in conf_dic_valid and tem != "AUC":

del conf_dic_valid[tem]

for key in conf_dic_train.keys():

mean_dic_train[key] = (

str(round_dec(float(mean_dic_train[key]), d=decimal_places))

+ "("

+ str(round_dec(float(conf_dic_train[key][0]), d=decimal_places))

+ "-"

+ str(round_dec(float(conf_dic_train[key][1]), d=decimal_places))

+ ")"

)

mean_dic_valid[key] = (

str(round_dec(float(mean_dic_valid[key]), d=decimal_places))

+ "("

+ str(round_dec(float(conf_dic_valid[key][0]), d=decimal_places))

+ "-"

+ str(round_dec(float(conf_dic_valid[key][1]), d=decimal_places))

+ ")"

)

df_train_result = pd.DataFrame([mean_dic_train], index=["Mean"])

df_valid_result = pd.DataFrame([mean_dic_valid], index=["Mean"])

df_train_result.rename(

columns={

"AUC": "AUC(95%CI)",

"cutoff": "cutoff(95%CI)",

"准确度": "准确度(95%CI)",

"灵敏度": "灵敏度(95%CI)",

"特异度": "特异度(95%CI)",

"阳性预测值": "阳性预测值(95%CI)",

"阴性预测值": "阴性预测值(95%CI)",

"F1分数": "F1分数(95%CI)",

"Kappa": "Kappa(95%CI)",

},

inplace=True,

)

df_valid_result.rename(

columns={

"AUC": "AUC(95%CI)",

"cutoff": "cutoff(95%CI)",

"准确度": "准确度(95%CI)",

"灵敏度": "灵敏度(95%CI)",

"特异度": "特异度(95%CI)",

"阳性预测值": "阳性预测值(95%CI)",

"阴性预测值": "阴性预测值(95%CI)",

"F1分数": "F1分数(95%CI)",

"Kappa": "Kappa(95%CI)",

},

inplace=True,

)

if has_testset:

test_result_dict = {

"AUC (95%CI)": (

str(round_dec(float(metric_dic_test["AUC"]), d=decimal_places))

+ " ("

+ str(

round_dec(float(metric_dic_test["AUC_L"]), d=decimal_places)

)

+ "-"

+ str(

round_dec(float(metric_dic_test["AUC_U"]), d=decimal_places)

)

+ ")"

)

}

for key in [

"cutoff",

"准确度",

"灵敏度",

"特异度",

"阳性预测值",

"阴性预测值",

"F1分数",

"Kappa",

]:

if key == "cutoff":

test_result_dict["cutoff"] = str(

round_dec(float(metric_dic_test[key]), d=decimal_places)

)

else:

lower_val, upper_val = conf_dic_test.get(

key, [metric_dic_test[key], metric_dic_test[key]]

)

test_result_dict[f"{key}(95%CI)"] = (

str(round_dec(float(metric_dic_test[key]), d=decimal_places))

+ "("

+ str(round_dec(float(lower_val), d=decimal_places))

+ "-"

+ str(round_dec(float(upper_val), d=decimal_places))

+ ")"

)

df_test_result = pd.DataFrame([test_result_dict], index=["Mean"])

new_order = ["AUC (95%CI)"] + [

col for col in df_test_result.columns if col != "AUC (95%CI)"

]

df_test_result = df_test_result[new_order]

elif resulttype == 0:

for tem in ["AUC_L", "AUC_U"]:

if tem in mean_dic_train:

del mean_dic_train[tem]

if tem in mean_dic_valid:

del mean_dic_valid[tem]

if tem in stdv_dic_train:

del stdv_dic_train[tem]

if tem in stdv_dic_valid:

del stdv_dic_valid[tem]

for key in stdv_dic_train.keys():

mean_dic_train[key] = (

str(round_dec(float(mean_dic_train[key]), d=decimal_places))

+ " ("

+ str(round_dec(float(stdv_dic_train[key]), d=decimal_places))

+ ")"

)

mean_dic_valid[key] = (

str(round_dec(float(mean_dic_valid[key]), d=decimal_places))

+ " ("

+ str(round_dec(float(stdv_dic_valid[key]), d=decimal_places))

+ ")"

)

df_train_result = pd.DataFrame([mean_dic_train], index=["Mean"])

df_valid_result = pd.DataFrame([mean_dic_valid], index=["Mean"])

df_train_result.rename(

columns={

"AUC": "AUC(SD)",

"cutoff": "cutoff(SD)",

"准确度": "准确度(SD)",

"灵敏度": "灵敏度(SD)",

"特异度": "特异度(SD)",

"阳性预测值": "阳性预测值(SD)",

"阴性预测值": "阴性预测值(SD)",

"F1分数": "F1分数(SD)",

"Kappa": "Kappa(SD)",

},

inplace=True,

)

df_valid_result.rename(

columns={

"AUC": "AUC(SD)",

"cutoff": "cutoff(SD)",

"准确度": "准确度(SD)",

"灵敏度": "灵敏度(SD)",

"特异度": "特异度(SD)",

"阳性预测值": "阳性预测值(SD)",

"阴性预测值": "阴性预测值(SD)",

"F1分数": "F1分数(SD)",

"Kappa": "Kappa(SD)",

},

inplace=True,

)

if has_testset:

test_result_dict = {

"AUC(SD)": (

str(round_dec(float(metric_dic_test["AUC"]), d=decimal_places))

+ " ("

+ str(

round_dec(

float(stdv_dic_test.get("AUC", 0.0)), d=decimal_places

)

)

+ ")"

)

}

for key in [

"cutoff",

"准确度",

"灵敏度",

"特异度",

"阳性预测值",

"阴性预测值",

"F1分数",

"Kappa",

]:

if key == "cutoff":

test_result_dict["cutoff"] = str(

round_dec(float(metric_dic_test[key]), d=decimal_places)

)

else:

test_result_dict[f"{key}(SD)"] = (

str(round_dec(float(metric_dic_test[key]), d=decimal_places))

+ " ("

+ str(

round_dec(

float(stdv_dic_test.get(key, 0.0)), d=decimal_places

)

)

+ ")"

)

df_test_result = pd.DataFrame([test_result_dict], index=["Mean"])

new_order = ["AUC(SD)"] + [

col for col in df_test_result.columns if col != "AUC(SD)"

]

df_test_result = df_test_result[new_order]

if modelsave:

if has_testset:

result_model_save.update(

{

"test_auc": float(metric_dic_test.get("AUC", 0)),

"test_accuracy": float(metric_dic_test.get("准确度", 0)),

}

)

else:

result_model_save.update(

{

"validation_auc": validation_auc_value,

"validation_accuracy": validation_accuracy_value,

}

)

df_dictjq = { # 结果数据表字典

"训练集结果汇总": df_train_result,

"验证集结果汇总": df_valid_result,

"训练集混淆矩阵": cm_df_train,

}

if has_testset:

df_dictjq.update(

{

"测试集结果汇总": df_test_result,

"测试集混淆矩阵": cm_df, # 混淆矩阵数据框

}

)

df_dict.update(df_dictjq)

\# 训练集与验证集校准曲线

if binary:

try:

train_calibration_curve_name, train_cal_desc = (

_plot_calibration_curve_from_predictions(

Ytrain,

y_pred_train,

name_dict[method],

output_dir=output_dir,

smooth=smooth,

image_format=image_format,

dpi=dpi,

legend_location=location,

decimal_places=decimal_places,

dataset_name="Train",

)

)

figures_list.append(

{

"name": "训练集校准曲线",

"position": "1-3",

"file": {

"jpeg": train_calibration_curve_name[0],

image_format: train_calibration_curve_name[1],

},

"description": (

"基于最终模型在训练集上的预测概率绘制。\n"

+ train_cal_desc

),

}

)

except Exception as e:

figures_list.append(

{

"name": "训练集校准曲线",

"file": {},

"description": f"这个图生成失败: {str(e)}",

}

)

finally:

plt.close("all")

try:

if not validation_curve_y_parts:

err_msg = "未收集到验证集预测概率。"

if validation_curve_collect_error:

err_msg += validation_curve_collect_error

raise ValueError(err_msg)

validation_curve_y = np.concatenate(validation_curve_y_parts)

validation_curve_probability = np.concatenate(

validation_curve_probability_parts

)

valid_calibration_curve_name, valid_cal_desc = (

_plot_calibration_curve_from_predictions(

validation_curve_y,

validation_curve_probability,

name_dict[method],

output_dir=output_dir,

smooth=smooth,

image_format=image_format,

dpi=dpi,

legend_location=location,

decimal_places=decimal_places,

dataset_name="Validation",

)

)

valid_calibration_source = (

"基于固定验证集预测概率绘制。\n"

if single_split_mode

else "基于各验证折预测概率汇总绘制。\n"

)

figures_list.append(

{

"name": "验证集校准曲线",

"position": "1-4",

"file": {

"jpeg": valid_calibration_curve_name[0],

image_format: valid_calibration_curve_name[1],

},

"description": valid_calibration_source + valid_cal_desc,

}

)

except Exception as e:

figures_list.append(

{

"name": "验证集校准曲线",

"file": {},

"description": f"这个图生成失败: {str(e)}",

}

)

finally:

plt.close("all")

\# 训练集与验证集 DCA 曲线

if binary:

try:

train_p_serie, train_net_benefit, train_treat_all = (

calculate_net_benefit_from_predictions(Ytrain, y_pred_train)

)

train_DCA_dict = {

name_dict[method]: {

"p_serie": train_p_serie,

"net_b_s": train_net_benefit,

"net_b_s_A": train_treat_all,

}

}

train_decision_curve_p, train_dca_desc = plot_decision_curves(

train_DCA_dict,

colors=palette_dict[style],

name="Train",

output_dir=output_dir,

dpi=dpi,

image_format=image_format,

DCA_cut=dca_cut,

legend_location=location,

)

figures_list.append(

{

"name": "训练集 DCA 曲线图",

"position": "3-1",

"file": {

"jpeg": train_decision_curve_p[0],

image_format: train_decision_curve_p[1],

},

"description": train_dca_desc,

}

)

except Exception as e:

figures_list.append(

{

"name": "训练集 DCA 曲线图",

"file": {},

"description": f"这个图生成失败: {str(e)}",

}

)

finally:

plt.close("all")

try:

if not validation_curve_y_parts:

err_msg = "未收集到验证集预测概率。"

if validation_curve_collect_error:

err_msg += validation_curve_collect_error

raise ValueError(err_msg)

validation_curve_y = np.concatenate(validation_curve_y_parts)

validation_curve_probability = np.concatenate(

validation_curve_probability_parts

)

valid_p_serie, valid_net_benefit, valid_treat_all = (

calculate_net_benefit_from_predictions(

validation_curve_y,

validation_curve_probability,

)

)

valid_DCA_dict = {

name_dict[method]: {

"p_serie": valid_p_serie,

"net_b_s": valid_net_benefit,

"net_b_s_A": valid_treat_all,

}

}

valid_decision_curve_p, valid_dca_desc = plot_decision_curves(

valid_DCA_dict,

colors=palette_dict[style],

name="Validation",

output_dir=output_dir,

dpi=dpi,

image_format=image_format,

DCA_cut=dca_cut,

legend_location=location,

)

valid_dca_source = (

"基于固定验证集预测概率绘制。\n"

if single_split_mode

else "基于各验证折预测概率汇总绘制。\n"

)

figures_list.append(

{

"name": "验证集 DCA 曲线图",

"position": "3-2",

"file": {

"jpeg": valid_decision_curve_p[0],

image_format: valid_decision_curve_p[1],

},

"description": valid_dca_source + valid_dca_desc,

}

)

except Exception as e:

figures_list.append(

{

"name": "验证集 DCA 曲线图",

"file": {},

"description": f"这个图生成失败: {str(e)}",

}

)

finally:

plt.close("all")

\# DCA 曲线

if binary and has_testset:

try:

DCA_dict = {}

(

prob_pos,

p_serie,

net_benefit_serie,

net_benefit_serie_All,

) = calculate_net_benefit(clf, Xtest, Ytest)

DCA_dict[name_dict[method]] = {

"p_serie": p_serie,

"net_b_s": net_benefit_serie,

"net_b_s_A": net_benefit_serie_All,

}

decision_curve_p, dca_desc = plot_decision_curves(

DCA_dict,

colors=palette_dict[style],

name="Test",

output_dir=output_dir,

dpi=dpi,

image_format=image_format,

DCA_cut=dca_cut,

legend_location=location,

)

figures_list.append(

{

"name": "测试集 DCA 曲线图",

"position": "3-3",

"file": {

"jpeg": decision_curve_p[0],

image_format: decision_curve_p[1],

},

"description": dca_desc,

}

)

except Exception as e:

figures_list.append(

{

"name": "测试集 DCA 曲线图",

"file": {},

"description": f"这个图生成失败: {str(e)}",

}

)

finally:

plt.close("all")

\# 混淆矩阵图

try:

plt.grid(False)

\# 处理训练集和验证集

if single_split_mode: # 模式 A

\# 画训练集

res_train = plot_conffusion_matrix_new(

clf,

Xtrain,

Ytrain,

group_labels,

resThreshold=resThreshold_train,

name="train_confusionMatrix_",

dic_name="最优模型训练集",

str_time=str_time,

output_dir=output_dir,

image_format=image_format,

dpi=600,

title="Confusion Matrix (Train Set)",

)

figures_list.append(

{

"name": "训练集混淆矩阵图",

"position": "3-4",

"file": {

"jpeg": res_train["pic"][next(iter(res_train["pic"]))],

image_format: res_train["save_pic"][

next(iter(res_train["save_pic"]))

],

},

"description": res_train.get("desc", "训练集混淆矩阵分析。"),

}

)

\# 2. 画验证集

res_valid = plot_conffusion_matrix_new(

clf,

X_valid,

Y_valid,

group_labels,

resThreshold=resThreshold_train,

name="valid_confusionMatrix_",

dic_name="最优模型验证集",

str_time=str_time,

output_dir=output_dir,

image_format=image_format,

dpi=600,

title="Confusion Matrix (Valid Set)",

)

figures_list.append(

{

"name": "验证集混淆矩阵图",

"position": "3-5",

"file": {

"jpeg": res_valid["pic"][next(iter(res_valid["pic"]))],

image_format: res_valid["save_pic"][

next(iter(res_valid["save_pic"]))

],

},

"description": res_valid.get("desc", "验证集混淆矩阵分析。"),

}

)

else: # 模式 B (Cross/Nest/Resample/Resample1无固定验证集)

\# 画训练集

res_train = plot_conffusion_matrix_new(

clf,

Xtrain,

Ytrain,

group_labels,

resThreshold=resThreshold_train,

name="train_confusionMatrix_",

dic_name="最优模型训练集",

str_time=str_time,

output_dir=output_dir,

image_format=image_format,

dpi=600,

title="Confusion Matrix (Train Set)",

)

figures_list.append(

{

"name": "训练集混淆矩阵图",

"position": "3-4",

"file": {

"jpeg": res_train["pic"][next(iter(res_train["pic"]))],

image_format: res_train["save_pic"][

next(iter(res_train["save_pic"]))

],

},

"description": res_train.get("desc", "训练集混淆矩阵分析。"),

}

)

if has_testset:

res_test = plot_conffusion_matrix_new(

clf,

Xtest,

Ytest,

group_labels,

resThreshold=resThreshold,

name="test_confusionMatrix_",

dic_name="最优模型测试集",

str_time=str_time,

output_dir=output_dir,

image_format=image_format,

dpi=600,

title="Confusion Matrix (Test Set)",

)

figures_list.append(

{

"name": "测试集混淆矩阵图",

"position": "3-6",

"file": {

"jpeg": res_test["pic"][next(iter(res_test["pic"]))],

image_format: res_test["save_pic"][

next(iter(res_test["save_pic"]))

],

},

"description": res_test.get("desc", "测试集混淆矩阵分析。"),

}

)

except Exception as e:

figures_list.append(

{

"name": "混淆矩阵图",

"file": {},

"description": f"图表生成失败: {str(e)}",

}

)

\# KS 曲线图

if has_testset:

try:

plot_name = "KS_" + str_time + ".jpeg"

plot_name_save = "KS_" + str_time + "." + image_format

y_probas = clf.predict_proba(Xtest)

y_true_binary = label_binarize(Ytest, classes=[0, 1]).flatten()

probs_class0 = y_probas[y_true_binary == 0, 0] # 负样本预测为0的概率

probs_class1 = y_probas[y_true_binary == 1, 0] # 正样本预测为0的概率

ks_stat, _ = ks_2samp(

probs_class1, probs_class0

) # 这里的计算方式可能因库而异，简单描述即可

ks_desc = f"KS 曲线分析：\nKS统计量用于评估模型区分正负样本的能力（数值越大越好）。本模型在测试集上的KS统计量表现如上图所示。"

skplt.metrics.plot_ks_statistic(

Ytest, y_probas, title="KS Statistic Plot(Test)"

)

_set_legend_style(location)

plt.savefig(

os.path.join(output_dir, plot_name),

dpi=dpi,

format="jpeg",

bbox_inches="tight",

)

plt.savefig(

os.path.join(output_dir, plot_name_save),

dpi=dpi,

format=image_format,

bbox_inches="tight",

)

plt.close()

figures_list.append(

{

"name": "测试集 KS 曲线",

"position": "4-1",

"file": {"jpeg": plot_name, image_format: plot_name_save},

"description": ks_desc,

}

)

except Exception as e:

figures_list.append(

{

"name": "KS 曲线",

"file": {},

"description": f"图表生成失败: {str(e)}",

}

)

\# 画 parallel_coordinates 图

if len(independent_variable) > 20:

figures_list.append(

{

"name": "平行坐标图",

"file": {},

"description": (

f"当前纳入变量数为 {len(independent_variable)} 个，超过平行坐标图建议展示上限 20 个。"

"为避免坐标标签重叠并影响图形可读性，系统已自动跳过该图绘制。"

),

}

)

else:

try:

\# 自动处理长变量

X_plot = df_input[independent_variable].copy()

y_plot = df_input[dependent_variable]

def split_label(label, max_len=8):

"""如果标签超过指定长度，在中间位置强制换行"""

if len(label) <= max_len:

return label

\# 计算中间位置

mid = len(label) // 2

return label[:mid] + "-\n" + label[mid:]

\# 应用换行逻辑到列名

new_columns = [split_label(col) for col in independent_variable]

X_plot.columns = new_columns

\# 设置画布

plt.figure(figsize=(12, 7), dpi=dpi)

visualizer = parallel_coordinates(

X_plot, y_plot, normalize=None, show=False

)

\# 生成默认元素

visualizer.finalize()

_set_legend_style(location)

plt.xticks(rotation=0, fontsize=9)

pc_desc = f"平行坐标图分析：\n该图展示了不同类别样本在 {len(independent_variable)} 个特征上的分布差异。"

pc_desc += " 每条线代表一个样本，颜色代表类别。如果您观察到某种颜色的线条在特定特征轴上聚集在较高或较低的区域，说明该特征对分类具有良好的区分度。"

plot_name = "parallel_coordinates_" + str_time + ".jpeg"

plot_name_save = "parallel_coordinates_" + str_time + "." + image_format

plt.savefig(

os.path.join(output_dir, plot_name),

dpi=dpi,

format="jpeg",

bbox_inches="tight",

)

plt.savefig(

os.path.join(output_dir, plot_name_save),

dpi=dpi,

format=image_format,

bbox_inches="tight",

)

plt.close()

figures_list.append(

{

"name": "平行坐标图",

"position": "4-2",

"file": {"jpeg": plot_name, image_format: plot_name_save},

"description": pc_desc,

}

)

except Exception as e:

figures_list.append(

{

"name": "平行坐标图",

"file": {},

"description": f"图表生成失败: {str(e)}",

}

)

\# 决策树图

if method == "DecisionTreeClassifier" and len(independent_variable) < 10:

import pydotplus

from sklearn import tree

from sklearn.tree import export_graphviz

try:

plot_name = "DecisionTree_pic_" + str_time + ".jpeg"

plot_name_save = "DecisionTree_pic_" + str_time + "." + image_format

group_u = list(

np.sort(np.unique(np.array(df_input[dependent_variable])))

)

group_name = [str(num) for num in group_u]

model_for_plot = clf

if searching == "auto":

if hasattr(searcher, "best_estimator_"):

model_for_plot = searcher.best_estimator_

dot_data = tree.export_graphviz(

model_for_plot,

out_file=None,

feature_names=independent_variable,

class_names=np.array(group_name),

filled=True,

rounded=True,

special_characters=True,

)

graph = pydotplus.graph_from_dot_data(dot_data)

graph.write_jpeg(os.path.join(output_dir, plot_name))

if image_format == "pdf":

graph.write_pdf(os.path.join(output_dir, plot_name_save))

else:

graph.write_jpeg(os.path.join(output_dir, plot_name_save))

dt_desc = "决策树可视化分析：\n根节点（最上方）代表了区分度最强的特征。每个节点中的 'gini' 或 'entropy' 表示纯度，'samples' 表示该节点的样本数，'value' 表示各类别的数量分布。"

dt_desc += " 您可以沿着路径向下，理解模型是如何通过一系列判定规则（如 X <= value）做出最终分类决策的。"

figures_list.append(

{

"name": "决策树图",

"position": "4-3",

"file": {"jpeg": plot_name, image_format: plot_name_save},

"description": dt_desc,

}

)

except Exception as e:

figures_list.append(

{

"name": "决策树图",

"file": {},

"description": f"图表生成失败: {str(e)}",

}

)

\# SHAP分析

if explain or modelsave:

try:

feature_names = Xtrain.columns.tolist()

f = lambda x: _shap_predict_proba(x, clf, feature_names)

\# 使用训练集中位数作为背景集（中性参考）

med = Xtrain.median().values.reshape((1, Xtrain.shape[1]))

if modelsave:

result_model_save.update(

{

"metadata": {"class": "Classification"},

"shap_value": [

float("{:.3f}".format(i)) for i in list(med[0])

],

"threshold": resThreshold,

}

)

if explain:

plt.rcParams["axes.grid"] = False

if shapSet == 2: # 测试集

if has_testset:

df_shap_global = Xtest

elif single_split_mode:

df_shap_global = X_valid

else:

df_shap_global = Xtrain

elif shapSet == 1: # 训练集

df_shap_global = Xtrain

else: # 全部数据 (0)

if has_testset:

df_shap_global = pd.concat([Xtrain, Xtest], axis=0)

elif single_split_mode:

df_shap_global = pd.concat([Xtrain, X_valid], axis=0)

else:

df_shap_global = Xtrain

\# 根据模型类型选择合适的解释器

if method in [

"DecisionTreeClassifier",

"RandomForestClassifier",

"CatBoostClassifier",

"LGBMClassifier",

"GradientBoostingClassifier",

]:

\# 使用TreeExplainer

explainer = shap.TreeExplainer(clf)

shap_values_global = explainer.shap_values(df_shap_global)

\# 对于二分类，TreeExplainer返回列表，取第二个元素（正类）

if isinstance(shap_values_global, list):

shap_values_global = shap_values_global[1]

else:

\# 其他模型使用KernelExplainer

f = lambda x: _shap_predict_proba(x, clf, feature_names)

med = Xtrain.median().values.reshape((1, Xtrain.shape[1]))

explainer = shap.KernelExplainer(f, med)

shap_values_raw = explainer.shap_values(df_shap_global)

if (

isinstance(shap_values_raw, list)

and len(shap_values_raw) > 1

):

shap_values_global = shap_values_raw[1]

else:

shap_values_global = shap_values_raw

\# 全局图表 (Summary / Bar / Radar)

if explain_numvar > 0:

\# Beeswarm Summary Plot

plt.clf()

shap.summary_plot(

shap_values_global,

df_shap_global,

show=False,

max_display=explain_numvar,

)

sum_jpeg = save_fig(

output_dir,

"shap_summary",

"jpeg",

plt.gcf(),

str_time=str_time,

)

sum_fmt = save_fig(output_dir, "shap_summary", image_format, plt.gcf(), str_time=str_time)

figures_list.append(

{

"name": "SHAP 变量贡献度总结",

"position": "5-1",

"file": {"jpeg": sum_jpeg, image_format: sum_fmt},

"description": "展示了特征值对预测的影响。红色代表高值，蓝色代表低值。",

}

)

plt.close()

\# Bar Plot

plt.clf()

shap.summary_plot(

shap_values_global,

df_shap_global,

plot_type="bar",

show=False,

max_display=explain_numvar,

)

bar_jpeg = save_fig(

output_dir,

"shap_import",

"jpeg",

plt.gcf(),

str_time=str_time,

)

bar_fmt = save_fig(output_dir, "shap_import", image_format, plt.gcf(), str_time=str_time)

figures_list.append(

{

"name": "SHAP 重要性图",

"position": "5-2",

"file": {"jpeg": bar_jpeg, image_format: bar_fmt},

"description": "按平均绝对影响排名的特征重要性。",

}

)

plt.close()

\# 特征依赖散点图

if shap_catter and len(shap_catter_feas) > 0:

for fea in shap_catter_feas:

try:

plt.clf()

shap.dependence_plot(

fea,

shap_values_global,

df_shap_global,

interaction_index=None,

show=False,

)

sc_jpeg = save_fig(

output_dir,

"shap_catter_" + fea,

"jpeg",

plt.gcf(),

str_time=str_time,

)

sc_fmt = save_fig(output_dir, "shap_catter_" + fea, image_format, plt.gcf(),

str_time=str_time)

figures_list.append(

{

"name": "SHAP 依赖图_" + fea,

"position": "5-3",

"file": {"jpeg": sc_jpeg, image_format: sc_fmt},

"description": f"展示特征 {fea} 的数值变化如何影响模型评分。",

}

)

plt.close()

except Exception as e:

figures_list.append(

{

"name": "SHAP依赖图",

"file": {},

"description": f"图表生成失败: {e}",

}

)

\# 相关性雷达图

if radar_plot:

try:

radar_names, radar_desc = plot_shap_radar(

shap_values_global,

feature_names,

output_dir,

str_time,

image_format=image_format,

dpi=dpi,

)

if radar_names:

figures_list.append(

{

"name": "特征相关性雷达图",

"position": "5-4",

"file": {

"jpeg": radar_names[0],

image_format: radar_names[1],

},

"description": radar_desc,

}

)

except Exception as e:

figures_list.append(

{

"name": "特征相关性雷达图",

"file": {},

"description": f"图表生成失败: {e}",

}

)

\# 个体样本解释

X_full = df_input[independent_variable] # 从全数据拿指定行

target_indices = (

[explain_indices]

if isinstance(explain_indices, int)

else explain_indices

)

for idx in target_indices:

if idx >= len(X_full):

continue

sample_row = X_full.iloc[idx : idx + 1, :]

sv_sample_raw = explainer.shap_values(sample_row)

sv_sample = (

sv_sample_raw[1][0]

if isinstance(sv_sample_raw, list)

else sv_sample_raw[0]

)

base_val = explainer.expected_value

if isinstance(base_val, (list, np.ndarray)):

base_val = base_val[1] if len(base_val) > 1 else base_val[0]

current_prob = f(sample_row)[0]

common_desc = (

f"样本(第{idx}行)分析：SHAP基准输出值 {base_val:.3f}，"

f"预测概率 {current_prob:.3f}。"

)

\# 力图

if shap_sample_type in ["force", "both"]:

plt.clf()

shap.force_plot(

base_val,

sv_sample,

sample_row.iloc[0, :],

matplotlib=True,

show=False,

)

sf_jpeg = save_fig(

output_dir,

f"shap_force_idx_{idx}",

"jpeg",

plt.gcf(),

str_time=str_time,

)

sf_fmt = save_fig(output_dir, f"shap_force_idx_{idx}", image_format, plt.gcf(),

str_time=str_time)

figures_list.append(

{

"name": f"样本{idx + 1}力图解释",

"position": "5-5",

"file": {"jpeg": sf_jpeg, image_format: sf_fmt},

"description": common_desc,

}

)

plt.close()

\# 瀑布图

if shap_sample_type in ["waterfall", "both"]:

plt.clf()

exp_obj = shap.Explanation(

values=sv_sample,

base_values=base_val,

data=sample_row.iloc[0, :].values,

feature_names=feature_names,

)

shap.waterfall_plot(exp_obj, show=False)

ax = plt.gca()

ax.grid(False, which="both") # 关闭所有网格

for gl in ax.xaxis.get_gridlines():

gl.set_visible(False)

for gl in ax.yaxis.get_gridlines():

gl.set_visible(False)

sw_jpeg = save_fig(

output_dir,

f"shap_waterfall_idx_{idx}",

"jpeg",

plt.gcf(),

str_time=str_time,

)

sw_fmt = save_fig(output_dir, f"shap_waterfall_idx_{idx}", image_format, plt.gcf(),

str_time=str_time)

figures_list.append(

{

"name": f"样本{idx}瀑布图解释",

"position": "5-6",

"file": {"jpeg": sw_jpeg, image_format: sw_fmt},

"description": common_desc,

}

)

plt.close()

if shap_circle:

try:

fig, ax = plt.subplots(figsize=(12, 10), dpi=300)

draw_shap_circle_plot(

clf,

df_shap_global,

independent_variable,

ax=ax,

top_features=10,

)

circle_name = "shap_interaction_network"

sc_jpeg = save_fig(

output_dir, circle_name, "jpeg", fig, str_time=str_time

)

sc_fmt = save_fig(

output_dir,

circle_name,

image_format,

fig,

str_time=str_time,

)

figures_list.append(

{

"name": "SHAP交互网络图",

"position": "5-7",

"file": {"jpeg": sc_jpeg, image_format: sc_fmt},

"description": "特征间交互强度分析：连线越粗交互越强，节点大小代表特征重要性。",

}

)

plt.close(fig)

except Exception as e:

figures_list.append(

{

"name": "SHAP交互网络图",

"file": {},

"description": f"图表生成失败: {e}",

}

)

if shap_rose:

try:

\# 传入已计算好的全局 SHAP 值和对应的全局数据集

rose_names, rose_desc = draw_shap_rose_plot_new(

shap_values_input=shap_values_global,

feature_names=independent_variable,

output_dir=output_dir,

str_time=str_time,

image_format=image_format,

dpi=dpi,

color_scheme=palette_dict[style][0],

title="SHAP_Bar_Rose",

)

if rose_names:

figures_list.append(

{

"name": "SHAP 综合玫瑰图",

"position": "5-8",

"file": {

"jpeg": rose_names[0],

image_format: rose_names[1],

},

"description": rose_desc,

}

)

except Exception as e:

figures_list.append(

{

"name": "SHAP综合玫瑰图",

"file": {},

"description": f"图表生成失败: {e}",

}

)

except Exception as e:

return {

"status": "error",

"description": str(e),

}

if searching == "auto" and "searcher" in locals():

param_source = getattr(searcher, "best_params_", clf.get_params())

param_name = method

else:

param_source = clf.get_params()

param_name = clf.__class__.__name__

param_text = dic2str(param_source, param_name)

if not str(param_text).strip():

param_items = list(param_source.items())[:20]

param_text = "\n".join([f"\t{k}: {v}" for k, v in param_items])

class_counts = []

for lab in sorted(df_input[dependent_variable].dropna().unique().tolist()):

class_counts.append((lab, int((df_input[dependent_variable] == lab).sum())))

validation_sizes = []

if "data_all" in locals() and isinstance(data_all, dict):

for data_value in data_all.values():

if isinstance(data_value, dict) and "Xvalid" in data_value:

validation_sizes.append(int(data_value["Xvalid"].shape[0]))

str_result = _build_binary_classification_description(

{

"method": method,

"method_label": name_dict.get(method, method),

"dependent_variable": dependent_variable,

"independent_variable": independent_variable,

"sample_count": int(df_input.shape[0]),

"class_counts": class_counts,

"binary": binary,

"searching": searching,

"auto_model": auto_model,

"param_text": param_text,

"has_testset": has_testset,

"testset": testset,

"trainset": trainset,

"isKFold": isKFold,

"n_splits": n_splits,

"val_method_name": val_method_name,

"train_pool_n": int(Xtrain.shape[0]) if Xtrain is not None else None,

"test_n": int(Xtest.shape[0]) if Xtest is not None else None,

"valid_n": int(X_valid.shape[0]) if X_valid is not None else None,

"validation_sizes": validation_sizes,

"validation_ratio": validation_ratio,

"test_ratio": test_ratio,

"label": label,

"testlabel": testlabel,

"trainlabel": trainlabel,

"mean_train": description_mean_train,

"mean_valid": description_mean_valid,

"metric_test": description_metric_test,

"std_train": description_std_train,

"std_valid": description_std_valid,

"std_test": description_std_test,

"resulttype": resulttype,

"decimal_places": decimal_places,

"figure_names": [fig.get("name", "") for fig in figures_list],

"save_str": save_str,

"timing_results": timing_results,

}

)

table_desc_mapping = {

"训练集结果汇总": f"基于{val_method_name}策略，展示模型在训练折/采样集上的平均性能指标及波动情况（SD/CI）。",

"验证集结果汇总": f"基于{val_method_name}策略，展示模型在验证折/留出集上的平均泛化能力，是调参的主要参考依据。",

"测试集结果汇总": "展示模型在完全独立的测试集上的最终性能，代表模型在实际应用中的预期表现。",

}

table_location_mapping = {

"训练集结果汇总": "6-1",

"验证集结果汇总": "6-2",

"测试集结果汇总": "6-3",

"测试集混淆矩阵": "7-1",

"训练集混淆矩阵": "7-2",

}

\# 转换表格格式

for table_name, df_obj in df_dict.items():

tables_list.append(

{

"name": table_name,

"position": table_location_mapping.get(table_name),

"file": {"dataframe": df_obj},

"description": table_desc_mapping.get(

table_name, f"包含 {table_name} 的详细统计数据。"

),

}

)

final_model_output = [result_model_save] if result_model_save else []

\# 最终返回结果

result_dict = {

"status": "success",

"description": str_result,

"tables": tables_list,

"figures": figures_list,

"model": final_model_output,

"environment": env_info,

}

return result_dict

\# 算法主体异常捕获

except Exception:

raise

\# 6. 外部验证

import datetime

import os

import random

import joblib

import matplotlib

import numpy as np

import pandas as pd

matplotlib.use("AGG")

import matplotlib as mpl

import matplotlib.pyplot as plt

from core.utils.get_environment import get_env_info

from core.utils.ml.assistant import (classification_metric_evaluate,

regression_metric_evaluate)

from core.utils.ml.plotdraw import (calculate_net_benefit, conf_matrix,

plot_calibration_curve,

plot_decision_curves)

mpl.rcParams["axes.grid"] = False

current_time = datetime.datetime.now().strftime("%Y-%m-%d_%H-%M-%S")

plt.rcParams["font.sans-serif"] = ["Times New Roman + SimSun"] # 用来正常显示中文标签

plt.rcParams["axes.unicode_minus"] = False # 用来正常显示负号)

plt.rcParams["ps.useafm"] = True

plt.rcParams["font.family"] = "sans-serif"

plt.rcParams["pdf.fonttype"] = 42

\# 获取环境信息

env_info = get_env_info(["pandas", "numpy", "matplotlib", "joblib"])

##通过模型识别码获模型文件路径，模型阈值，

def external_validation(

df_input,

dependent_variable,

independent_variable,

modelpath,

output_dir,

cutoff,

method="Classification",

model_name="XGBClassifier",

image_format="jpeg",

dpi=600,

decimal_places=3,

):

"""

外部验证

:param df_input: dataframe 分析数据

:param dependent_variable: str 结局(因变量)

:param independent_variable: list 自变量

:param modelpath: str 模型路径

:param cutoff: float 模型阈值

:param output_dir: str 图片存储路径

:param method: str 模型方法 ('Classification':二分类, 'Regression'：回归, 'Multiclassification'：多分类)

:param model_name: str 模型名称

:param image_format: str

:param dpi: int 图像分辨率

:param decimal_places:int 保留小数点数

"""

\# 初始化返回容器

figures_list = []

tables_list = []

str_res = ""

\# 颜色配置

name_dict = {

"LogisticRegression": "logistic",

"XGBClassifier": "XGBoost",

"RandomForestClassifier": "RandomForest",

"LGBMClassifier": "LightGBM",

"SVC": "SVM",

"MLPClassifier": "MLP",

"GaussianNB": "GNB",

"ComplementNB": "CNB",

"AdaBoostClassifier": "AdaBoost",

"KNeighborsClassifier": "KNN",

"DecisionTreeClassifier": "DecisionTree",

"BaggingClassifier": "Bagging",

"GradientBoostingClassifier": "GBDT",

"LogisticAT": "LogisticAT",

"LinearRegression": "LinearReg",

"XGBRegressor": "XGBoostReg",

"RandomForestRegressor": "RandomForestReg",

"LinearSVR": "LinearSVR",

"KNeighborsRegressor": "KNNReg",

}

colors = [

"#BC3C29FF",

"#0072B5FF",

"#E18727FF",

"#20854EFF",

"#7876B1FF",

"#6F99ADFF",

"#FFDC91FF",

"#EE4C97FF",

"#BC3C29FF",

]

str_time = (

datetime.datetime.now().strftime("%H%M%S") + f"_{random.randint(1, 1000):04d}"

)

methods = {"Classification", "Regression", "Multiclassification"}

try:

if method not in methods:

raise ValueError(f"暂不支持{method}做外部验证")

\# 加载模型并提取模型内部记录的特征名称

try:

model = joblib.load(modelpath)

except Exception as e:

raise ValueError(f"模型加载失败: {e}")

\# 尝试获取模型训练时的特征顺序

model_features = None

if hasattr(model, "feature_names_in_"):

model_features = list(model.feature_names_in_)

elif hasattr(model, "get_booster"):

\# XGBoost 特殊处理

model_features = model.get_booster().feature_names

\# 确定最终使用的特征列表

final_features = independent_variable

if model_features:

\# 检查缺失列

missing_cols = set(model_features) - set(df_input.columns)

if missing_cols:

raise ValueError(f"输入数据缺少模型所需的列: {missing_cols}")

\# 强制重排序

final_features = model_features

str_res += f"已自动对齐特征顺序: {final_features}\n"

else:

str_res += "警告：未能从模型检测到特征名称，将使用用户提供的变量顺序。请确保顺序与训练时完全一致！\n"

try:

\# 确保提取的列包含模型所需特征

cols_to_extract = list(

set(independent_variable + final_features + [dependent_variable])

)

res_df = df_input[cols_to_extract].dropna()

missing_in_res = set(final_features) - set(res_df.columns)

if missing_in_res:

raise ValueError(f"处理后的数据缺少模型所需特征: {missing_in_res}")

except KeyError as e:

raise ValueError(f"数据中缺少必要的列: {e}")

except Exception as e:

raise ValueError(f"数据提取或去空值时发生错误: {e}")

if method == "Classification":

un_group = np.sort(np.unique(np.array(res_df[dependent_variable])))

if not set(un_group).issubset({0, 1}):

raise ValueError(f"二分类标签必须为 0 或 1，检测到标签：{un_group}")

elif method == "Multiclassification":

un_group = np.sort(np.unique(np.array(df_input[dependent_variable])))

if len(un_group) > 10:

raise ValueError(

"暂不允许类别数目大于10的情况。请检查结局变量取值情况。"

)

\# 加载模型

model = joblib.load(filename=modelpath)

str_res += (

"在"

+ method

+ "中建模的方法为："

+ name_dict.get(model_name, model_name)

+ "模型的完整参数为："

+ str(model.get_params())

+ "\n"

)

\# 二分类

if method == "Classification":

\# 计算指标

(

fpr_test,

tpr_test,

metric_dic_test,

df_test_result,

) = classification_metric_evaluate(

clf=model,

Xtest=res_df[final_features],

Ytest=res_df[[dependent_variable]],

binary=True,

Threshold=cutoff,

)

df_test_result = df_test_result.applymap(lambda x: round(x, decimal_places))

\# 表格

tables_list.append(

{

"name": "测试集分析表",

"file": {"dataframe": df_test_result},

"description": "包含测试集各项分类指标。",

}

)

\# 绘制ROC曲线

try:

fig = plt.figure(figsize=(4, 4), dpi=dpi)

plt.plot([0, 1], [0, 1], linestyle="--", lw=1, color="r", alpha=0.8)

plt.grid(

which="major", axis="both", linestyle="-.", alpha=0.08, color="grey"

)

plt.plot(

fpr_test,

tpr_test,

lw=1.5,

alpha=0.6,

color="b",

label="Test Set ROC (auc=%0.3f 95%%CI (%0.3f-%0.3f)) "

% (

metric_dic_test["AUC"],

metric_dic_test["AUC_L"],

metric_dic_test["AUC_U"],

),

)

plt.xlim([-0.02, 1.02])

plt.ylim([-0.02, 1.02])

plt.xlabel("1-Specificity")

plt.ylabel("Sensitivity")

plt.title("ROC curve(Test)")

plt.legend(loc="lower right", fontsize=5)

\# 保存逻辑优化

plot_base_name = f"ROC_curve_test_{str_time}"

plot_name_jpeg = f"{plot_base_name}.jpeg"

plot_name_custom = f"{plot_base_name}.{image_format}"

if output_dir is not None:

plt.savefig(

os.path.join(output_dir, plot_name_jpeg),

dpi=dpi,

format="jpeg",

bbox_inches="tight",

)

plt.savefig(

os.path.join(output_dir, plot_name_custom),

dpi=dpi,

format=image_format,

bbox_inches="tight",

)

figures_list.append(

{

"name": "测试集ROC曲线",

"file": {

"jpeg": plot_name_jpeg,

image_format: plot_name_custom,

},

"description": "测试集上的ROC曲线。",

}

)

except Exception as e:

figures_list.append(

{

"name": "测试集ROC曲线",

"file": {},

"description": f"生成失败: {e}",

}

)

finally:

plt.close(fig)

\# 绘制 DCA 曲线

try:

DCA_dict = {}

prob_pos, p_serie, net_benefit_serie, net_benefit_serie_All = (

calculate_net_benefit(

model,

Xtest=res_df[final_features],

Ytest=res_df[[dependent_variable]],

)

)

DCA_dict[name_dict.get(model_name, model_name)] = {

"p_serie": p_serie,

"net_b_s": net_benefit_serie,

"net_b_s_A": net_benefit_serie_All,

}

\# 接收

dca_files, desc_str = plot_decision_curves(

DCA_dict,

colors=colors,

name="Test",

output_dir=output_dir,

dpi=dpi,

image_format=image_format,

DCA_cut=0,

)

figures_list.append(

{

"name": "测试集DCA曲线图",

"file": {

"jpeg": os.path.basename(dca_files[0]),

image_format: os.path.basename(dca_files[1]),

},

"description": desc_str,

}

)

except Exception as e:

figures_list.append(

{

"name": "测试集DCA曲线图",

"file": {},

"description": f"生成失败: {e}",

}

)

\# 校准曲线

try:

calibration_curve_name, _ = plot_calibration_curve(

model,

X_train=res_df[final_features],

X_test=res_df[final_features],

y_train=res_df[[dependent_variable]],

y_test=res_df[[dependent_variable]],

name=name_dict.get(model_name, model_name),

output_dir=output_dir,

smooth=True,

image_format=image_format,

dpi=600,

istraing=False,

)

figures_list.append(

{

"name": "测试集校准曲线",

"file": {

"jpeg": os.path.basename(calibration_curve_name[0]),

image_format: os.path.basename(calibration_curve_name[1]),

},

"description": "测试集校准曲线。",

}

)

except Exception as e:

figures_list.append(

{

"name": "测试集校准曲线",

"file": {},

"description": f"生成失败: {e}",

}

)

\# 混淆矩阵

if method == "Classification" or method == "Multiclassification":

try:

un_group = np.sort(np.unique(np.array(res_df[[dependent_variable]])))

test_report = conf_matrix(

model,

x_data=res_df[final_features],

y_data=res_df[[dependent_variable]],

un_group=un_group,

pic_name="testset",

str_time=str_time,

random_number=2,

output_dir=output_dir,

image_format=image_format,

dpi=dpi,

decimal_places=decimal_places,

Threshold=cutoff,

)

if method in ["Classification", "Multiclassification"]:

tables_list.append(

{

"name": "测试集分析表",

"file": {"dataframe": test_report["tab"]},

"description": "多分类混淆矩阵报告。",

}

)

pic_dict = test_report.get("pic", {})

save_pic_dict = test_report.get("save_pic", {})

for key in pic_dict.keys():

jpeg_file = os.path.basename(pic_dict[key])

custom_file = os.path.basename(save_pic_dict.get(key, ""))

figures_list.append(

{

"name": key,

"file": {"jpeg": jpeg_file, image_format: custom_file},

"description": "混淆矩阵热图。",

}

)

except Exception as e:

figures_list.append(

{

"name": "混淆矩阵热图",

"file": {},

"description": f"生成失败: {e}",

}

)

\# 回归 (Regression)

if method == "Regression":

res_pred = model.predict(res_df[final_features])

df_result, metric_dic = regression_metric_evaluate(

model, res_df[final_features], res_df[[dependent_variable]]

)

df_result = df_result.applymap(lambda x: round(x, decimal_places))

tables_list.append(

{

"name": "测试集分析表",

"file": {"dataframe": df_result},

"description": "回归模型评价指标。",

}

)

Y_test = np.array(res_df[[dependent_variable]])

\# 降维

Ytest_flat = Y_test.ravel()

\# 范围比较图

try:

fig1, ax1 = plt.subplots(figsize=(4, 4), dpi=dpi)

ax1.scatter(

Ytest_flat,

res_pred,

edgecolors="none",

marker="*",

color="#2CBDFE",

s=50.0,

)

ax1.spines["top"].set_visible(False)

ax1.spines["right"].set_visible(False)

ax1.grid(which="major", axis="both", linestyle="-.", alpha=0.2)

ax1.spines["left"].set_position("center")

ax1.spines["bottom"].set_position("center")

ax1.plot(

[Ytest_flat.min(), Ytest_flat.max()],

[Ytest_flat.min(), Ytest_flat.max()],

"k--",

lw=2,

)

ax1.set_xlabel("True", fontsize="x-small", loc="right")

ax1.set_ylabel("Predicted", fontsize="x-small", loc="top")

base_name1 = f"Range_Comparison_{str_time}"

if output_dir is not None:

plt.savefig(

os.path.join(output_dir, f"{base_name1}.jpeg"),

dpi=dpi,

format="jpeg",

bbox_inches="tight",

)

plt.savefig(

os.path.join(output_dir, f"{base_name1}.{image_format}"),

dpi=dpi,

format=image_format,

bbox_inches="tight",

)

figures_list.append(

{

"name": "比较图",

"file": {

"jpeg": f"{base_name1}.jpeg",

image_format: f"{base_name1}.{image_format}",

},

"description": "真实值与预测值的散点比较。",

}

)

except Exception as e:

figures_list.append(

{"name": "比较图", "file": {}, "description": f"生成失败: {e}"}

)

finally:

plt.close(fig1)

\# 真实预测比较图 (线性)

try:

fig2, ax2 = plt.subplots(figsize=(8, 4), dpi=dpi)

ax2.spines["top"].set_visible(False)

ax2.spines["right"].set_visible(False)

ax2.spines["bottom"].set_visible(False)

ax2.tick_params(bottom=False)

ax2.grid(which="major", axis="y", linestyle="--")

ax2.plot(

np.arange(res_df[final_features].shape[0]),

Ytest_flat,

color="k",

label="True",

)

ax2.plot(

np.arange(res_df[final_features].shape[0]),

res_pred,

color="r",

label=method + "Predicted",

)

ax2.set_xlabel("Data Samples", fontsize="x-small", loc="center")

ax2.set_ylabel("Value", fontsize="x-small", loc="top")

ax2.legend(loc="lower right")

base_name2 = f"True_vs_Predicted_{str_time}"

if output_dir is not None:

plt.savefig(

os.path.join(output_dir, f"{base_name2}.jpeg"),

dpi=dpi,

format="jpeg",

bbox_inches="tight",

)

plt.savefig(

os.path.join(output_dir, f"{base_name2}.{image_format}"),

dpi=dpi,

format=image_format,

bbox_inches="tight",

)

figures_list.append(

{

"name": "真实预测比较图",

"file": {

"jpeg": f"{base_name2}.jpeg",

image_format: f"{base_name2}.{image_format}",

},

"description": "样本序列上的真实值与预测值对比。",

}

)

except Exception as e:

figures_list.append(

{

"name": "真实预测比较图",

"file": {},

"description": f"生成失败: {e}",

}

)

finally:

plt.close(fig2)

\# 残差图

try:

fig3, ax3 = plt.subplots(figsize=(8, 4), dpi=dpi)

ax3.scatter(

Ytest_flat,

res_pred - Ytest_flat,

marker="X",

edgecolors="none",

color="#661D98",

s=50.0,

)

ax3.spines["top"].set_visible(False)

ax3.spines["right"].set_visible(False)

ax3.spines["left"].set_position("center")

ax3.axhline(y=0, color="r", linestyle="--")

ax3.grid(which="major", axis="y", linestyle="-.")

ax3.set_xlabel("True Value", fontsize="x-small", loc="right")

ax3.set_ylabel("Residual", fontsize="x-small", loc="top")

base_name3 = f"Residual_{str_time}"

if output_dir is not None:

plt.savefig(

os.path.join(output_dir, f"{base_name3}.jpeg"),

dpi=dpi,

format="jpeg",

bbox_inches="tight",

)

plt.savefig(

os.path.join(output_dir, f"{base_name3}.{image_format}"),

dpi=dpi,

format=image_format,

bbox_inches="tight",

)

figures_list.append(

{

"name": "残差图",

"file": {

"jpeg": f"{base_name3}.jpeg",

image_format: f"{base_name3}.{image_format}",

},

"description": "预测残差分布图。",

}

)

except Exception as e:

figures_list.append(

{"name": "残差图", "file": {}, "description": f"生成失败: {e}"}

)

finally:

plt.close(fig3)

\# 构建最终返回

result_dict = {

"status": "success",

"description": str_res,

"tables": tables_list,

"figures": figures_list,

"environment": env_info,

}

return result_dict

\# 算法主体异常捕获

except Exception:

raise

\# 7. 模型解释：SHAP

import datetime

import random

import matplotlib.pyplot as plt

import numpy as np

import pandas as pd

import shap

from core.utils.ml.assistant import save_fig

from core.utils.ml.plotdraw import draw_shap_circle_plot, draw_shap_rose_plot_new, plot_shap_radar

def _shap_predict_proba(x, model, feature_names):

"""

SHAP 安全预测函数：保证输入有 feature names

x:array-like/DataFrame 待解释样本

model:object 已训练分类模型

feature_names:list 特征名称

"""

if not isinstance(x, pd.DataFrame):

x = pd.DataFrame(x, columns=feature_names)

return model.predict_proba(x)[:, 1]

def shap_model_explanation(

clf,

Xtrain,

independent_variable,

output_dir,

Xtest=None,

X_valid=None,

df_input=None,

has_testset=False,

single_split_mode=False,

shapSet=2,

explain_numvar=2,

explain_indices=0,

shap_catter=False,

shap_catter_feas=None,

shap_circle=False,

radar_plot=False,

shap_rose=False,

shap_sample_type="waterfall",

style="lancet",

image_format="jpeg",

dpi=600,

):

"""

SHAP 模型解释

clf:object 已训练模型

Xtrain:DataFrame 训练集特征数据

independent_variable:list 自变量名称

output_dir:str 图片保存路径

Xtest:DataFrame 测试集特征数据

X_valid:DataFrame 验证集特征数据

df_input:DataFrame 原始数据，用于按行索引进行个体解释

has_testset:bool 是否存在独立测试集

single_split_mode:bool 是否为单次验证集划分模式

shapSet:int 解释数据集，2为测试/验证集，1为训练集，0为全部可用数据

explain_numvar:int 展示的变量数量

explain_indices:int/list 需要解释的样本行索引

shap_catter:bool 是否绘制依赖图

shap_catter_feas:list 需要绘制依赖图的特征

shap_circle:bool 是否绘制交互网络图

radar_plot:bool 是否绘制雷达图

shap_rose:bool 是否绘制玫瑰图

shap_sample_type:str 个体解释图类型，可选 force、waterfall、both

style:str 配色方案

image_format:str 图片保存格式

dpi:int 图片分辨率

"""

figures_list = []

if shap_catter_feas is None:

shap_catter_feas = []

if df_input is None:

df_input = Xtrain.copy()

if explain_numvar in (None, ""):

explain_numvar = len(independent_variable)

else:

explain_numvar = min(int(explain_numvar), len(independent_variable))

str_time = datetime.datetime.now().strftime("%H%M%S") + str(random.randint(1, 100))

palette_dict = {

"lancet": ["#00468BFF", "#ED0000FF", "#42B540FF", "#0099B4FF", "#925E9FFF"],

"nejm": ["#BC3C29FF", "#0072B5FF", "#E18727FF", "#20854EFF", "#7876B1FF"],

"jama": ["#374E55FF", "#DF8F44FF", "#00A1D5FF", "#B24745FF", "#79AF97FF"],

"npg": ["#E64B35FF", "#4DBBD5FF", "#00A087FF", "#3C5488FF", "#F39B7FFF"],

}

try:

feature_names = Xtrain.columns.tolist()

predict_function = lambda x: _shap_predict_proba(x, clf, feature_names)

if shapSet == 2:

if has_testset and Xtest is not None:

df_shap_global = Xtest

elif single_split_mode and X_valid is not None:

df_shap_global = X_valid

else:

df_shap_global = Xtrain

elif shapSet == 1:

df_shap_global = Xtrain

else:

data_parts = [Xtrain]

if has_testset and Xtest is not None:

data_parts.append(Xtest)

elif single_split_mode and X_valid is not None:

data_parts.append(X_valid)

df_shap_global = pd.concat(data_parts, axis=0)

tree_model_names = [

"DecisionTreeClassifier",

"RandomForestClassifier",

"CatBoostClassifier",

"LGBMClassifier",

"GradientBoostingClassifier",

]

if clf.__class__.__name__ in tree_model_names:

explainer = shap.TreeExplainer(clf)

shap_values_global = explainer.shap_values(df_shap_global)

if isinstance(shap_values_global, list):

shap_values_global = shap_values_global[1]

else:

background = Xtrain.median().values.reshape((1, Xtrain.shape[1]))

explainer = shap.KernelExplainer(predict_function, background)

shap_values_raw = explainer.shap_values(df_shap_global)

if isinstance(shap_values_raw, list) and len(shap_values_raw) > 1:

shap_values_global = shap_values_raw[1]

else:

shap_values_global = shap_values_raw

if explain_numvar > 0:

try:

plt.clf()

shap.summary_plot(

shap_values_global,

df_shap_global,

show=False,

max_display=explain_numvar,

)

sum_jpeg = save_fig(output_dir, "shap_summary", "jpeg", plt.gcf(), str_time=str_time)

sum_fmt = save_fig(output_dir, "shap_summary", image_format, plt.gcf(), str_time=str_time)

figures_list.append({

"name": "SHAP 变量贡献度总结",

"file": {"jpeg": sum_jpeg, image_format: sum_fmt},

"description": "展示特征值对预测结果的影响方向和强度。",

})

except Exception as e:

figures_list.append({"name": "SHAP 变量贡献度总结", "file": {}, "description": f"这个图生成失败: {str(e)}"})

finally:

plt.close("all")

try:

plt.clf()

shap.summary_plot(

shap_values_global,

df_shap_global,

plot_type="bar",

show=False,

max_display=explain_numvar,

)

bar_jpeg = save_fig(output_dir, "shap_import", "jpeg", plt.gcf(), str_time=str_time)

bar_fmt = save_fig(output_dir, "shap_import", image_format, plt.gcf(), str_time=str_time)

figures_list.append({

"name": "SHAP 重要性图",

"file": {"jpeg": bar_jpeg, image_format: bar_fmt},

"description": "按平均绝对 SHAP 值排序展示变量重要性。",

})

except Exception as e:

figures_list.append({"name": "SHAP 重要性图", "file": {}, "description": f"这个图生成失败: {str(e)}"})

finally:

plt.close("all")

if shap_catter and len(shap_catter_feas) > 0:

for feature in shap_catter_feas:

try:

plt.clf()

shap.dependence_plot(

feature,

shap_values_global,

df_shap_global,

interaction_index=None,

show=False,

)

sc_jpeg = save_fig(output_dir, "shap_catter_" + feature, "jpeg", plt.gcf(), str_time=str_time)

sc_fmt = save_fig(output_dir, "shap_catter_" + feature, image_format, plt.gcf(), str_time=str_time)

figures_list.append({

"name": "SHAP 依赖图_" + feature,

"file": {"jpeg": sc_jpeg, image_format: sc_fmt},

"description": f"展示特征 {feature} 的数值变化如何影响模型预测。",

})

except Exception as e:

figures_list.append({"name": "SHAP依赖图", "file": {}, "description": f"这个图生成失败: {str(e)}"})

finally:

plt.close("all")

if radar_plot:

try:

radar_names, radar_desc = plot_shap_radar(

shap_values_global,

feature_names,

output_dir,

str_time,

image_format=image_format,

dpi=dpi,

)

if radar_names:

figures_list.append({

"name": "特征相关性雷达图",

"file": {"jpeg": radar_names[0], image_format: radar_names[1]},

"description": radar_desc,

})

except Exception as e:

figures_list.append({"name": "特征相关性雷达图", "file": {}, "description": f"这个图生成失败: {str(e)}"})

finally:

plt.close("all")

X_full = df_input[independent_variable]

target_indices = [explain_indices] if isinstance(explain_indices, int) else explain_indices

for idx in target_indices:

if idx >= len(X_full):

continue

sample_row = X_full.iloc[idx: idx + 1, :]

shap_values_sample_raw = explainer.shap_values(sample_row)

shap_values_sample = (

shap_values_sample_raw[1][0]

if isinstance(shap_values_sample_raw, list)

else shap_values_sample_raw[0]

)

base_value = explainer.expected_value

if isinstance(base_value, (list, np.ndarray)):

base_value = base_value[1] if len(base_value) > 1 else base_value[0]

current_prob = predict_function(sample_row)[0]

common_desc = f"样本(第{idx}行)分析：SHAP基准输出值 {base_value:.3f}，预测概率 {current_prob:.3f}。"

if shap_sample_type in ["force", "both"]:

try:

plt.clf()

shap.force_plot(base_value, shap_values_sample, sample_row.iloc[0, :], matplotlib=True, show=False)

sf_jpeg = save_fig(output_dir, f"shap_force_idx_{idx}", "jpeg", plt.gcf(), str_time=str_time)

sf_fmt = save_fig(output_dir, f"shap_force_idx_{idx}", image_format, plt.gcf(), str_time=str_time)

figures_list.append({

"name": f"样本{idx + 1}力图解释",

"file": {"jpeg": sf_jpeg, image_format: sf_fmt},

"description": common_desc,

})

except Exception as e:

figures_list.append({"name": "SHAP力图解释", "file": {}, "description": f"这个图生成失败: {str(e)}"})

finally:

plt.close("all")

if shap_sample_type in ["waterfall", "both"]:

try:

plt.clf()

explanation_object = shap.Explanation(

values=shap_values_sample,

base_values=base_value,

data=sample_row.iloc[0, :].values,

feature_names=feature_names,

)

shap.waterfall_plot(explanation_object, show=False)

ax = plt.gca()

ax.grid(False, which="both")

sw_jpeg = save_fig(output_dir, f"shap_waterfall_idx_{idx}", "jpeg", plt.gcf(), str_time=str_time)

sw_fmt = save_fig(output_dir, f"shap_waterfall_idx_{idx}", image_format, plt.gcf(), str_time=str_time)

figures_list.append({

"name": f"样本{idx}瀑布图解释",

"file": {"jpeg": sw_jpeg, image_format: sw_fmt},

"description": common_desc,

})

except Exception as e:

figures_list.append({"name": "SHAP瀑布图解释", "file": {}, "description": f"这个图生成失败: {str(e)}"})

finally:

plt.close("all")

if shap_circle:

try:

fig, ax = plt.subplots(figsize=(12, 10), dpi=dpi)

draw_shap_circle_plot(clf, df_shap_global, independent_variable, ax=ax, top_features=10)

circle_jpeg = save_fig(output_dir, "shap_interaction_network", "jpeg", fig, str_time=str_time)

circle_fmt = save_fig(output_dir, "shap_interaction_network", image_format, fig, str_time=str_time)

figures_list.append({

"name": "SHAP交互网络图",

"file": {"jpeg": circle_jpeg, image_format: circle_fmt},

"description": "特征间交互强度分析：连线越粗交互越强，节点大小代表特征重要性。",

})

except Exception as e:

figures_list.append({"name": "SHAP交互网络图", "file": {}, "description": f"这个图生成失败: {str(e)}"})

finally:

plt.close("all")

if shap_rose:

try:

rose_names, rose_desc = draw_shap_rose_plot_new(

shap_values_input=shap_values_global,

feature_names=independent_variable,

output_dir=output_dir,

str_time=str_time,

image_format=image_format,

dpi=dpi,

color_scheme=palette_dict.get(style, palette_dict["lancet"])[0],

title="SHAP_Bar_Rose",

)

if rose_names:

figures_list.append({

"name": "SHAP 综合玫瑰图",

"file": {"jpeg": rose_names[0], image_format: rose_names[1]},

"description": rose_desc,

})

except Exception as e:

figures_list.append({"name": "SHAP综合玫瑰图", "file": {}, "description": f"这个图生成失败: {str(e)}"})

finally:

plt.close("all")

return {

"status": "success",

"description": "SHAP 模型解释完成。",

"tables": [],

"figures": figures_list,

}

except Exception:

raise
